# Empirical assessment of sequencing errors for high throughput pyrosequencing data

**DOI:** 10.1186/1756-0500-6-25

**Published:** 2013-01-22

**Authors:** Paulo GS da Fonseca, Jorge AP Paiva, Luiz GP Almeida, Ana TR Vasconcelos, Ana T Freitas

**Affiliations:** 1 Instituto de Engenharia de Sistemas e Computadores: Investigação e Desenvolvimento (INESC-ID), R. Alves Redol 9, Lisboa 1000-029, Portugal; 2 Centro de Informática–Universidade Federal de Pernambuco, Av. Jornalista Anibal Fernandes s/n, Cidade Universitária Recife - PE 50740-560, Brasil; 3 Instituto de Investigação Científica Tropical (IICT), Centro de Florestas e dos Produtos Florestais, Tapada da Ajuda, Lisboa 1349-018, Portugal; 4 Laboratório Nacional de Computação Científica (LNCC), Laboratório de Bioinformática, Av. Getúlio Vargas, 333 Petrópolis, Rio de Janeiro, Brasil; 5 Instituto Superior Técnico–Universidade Técnica de Lisboa (IST/UTL), Av. Rovisco Pais, Lisboa 1049-001, Portugal

## Abstract

**Background:**

Sequencing-by-synthesis technologies significantly improve over the Sanger method in terms of speed and cost per base. However, they still usually fail to compete in terms of read length and quality. Current high-throughput implementations of the pyrosequencing technique yield reads whose length approach those of the capillary electrophoresis method. A less obvious question is whether their quality is affected by platform-specific sequencing errors.

**Results:**

We present an empirical study aimed at assessing the quality and characterising sequencing errors for high throughput pyrosequencing data. We have developed a procedure for extracting sequencing error data from genome assemblies and study their characteristics, in particular the length distribution of indel gaps and their relation to the sequence contexts where they occur. We used this procedure to analyse data from three prokaryotic genomes sequenced with the GS FLX technology. We also compared two models previously employed with success for peptide sequence alignment.

**Conclusions:**

We observed an overall very low error rate in the analysed data, with indel errors being much more abundant than substitutions. We also observed a dependence between the length of the gaps and that of the homopolymer context where they occur. As with protein alignments, a power-law model seems to approximate the indel errors more accurately, although the results are not so conclusive as to justify a depart from the commonly used affine gap penalty scheme. In whichever case, however, our procedure can be used to estimate more realistic error model parameters.

## Background

High throughput sequencing technology (HTST) has been changing the landscape of biomedical research, allowing for issues like genetic variation to be analysed at a much higher resolution and lower cost [1]. However, the accumulation of massive amounts of sequence data has brought back to the spotlight old bioinformatics problems of string pattern matching and fragment assembly which, although not completely solved, had been satisfactorily dealt with in the context of traditional capillary electrophoresis sequencing over the last years. The difficulties raised by HTST stem naturally from the sheer volume of data produced but also from their characteristics, in particular the relative smaller read size and the platform-specific sequencing errors.

Mainstream competing HTSTs offer different trade-offs in terms of throughput, cost and sequence characteristics, including read length and accuracy [2]. Common to them all is the use of highly parallelised *sequencing-by-synthesis* procedures that dispense with the laborious bacterial clone library preparation in favour of specialised forms of *in vitro* amplification of the single-stranded DNA library templates. In the *pyrosequencing* methodology [3] commercialised by Roche/454 Life Sciences through their GS FLX™ platform [4] the process is carried out in cycles during which a solution of each of the four distinct deoxribonucleotide triphosphates (dNTP) is, in turn, flowed into a substrate containing the immobilised single stranded DNA template and the DNA polymerase. The eventual polymerase-mediated incorporation of complementary dNTPs to the template triggers a chain of reactions involving chemiluminescent enzymes also present in the medium resulting in the emission of a burst of light of intensity proportional to the number of nucleotides added. This process is highly parallelised as the solid-phase substrate (the PicoTiterPlate) is organised as an array of over a million and a half independent spots, each corresponding to a well containing one bead to which million copies of a same template molecule are attached. The light emitted at each cycle and at each spot is captured by a coupled digital imaging device, producing a series of pictures which are processed by an image-analysis program, giving rise to the *flowgrams* (the pyrosequencing counterparts of Sanger chromatograms) from which the identity of the templates can be inferred.

The GS FLX system yields reads a few hundred bases long with an overall quality that has allowed for *de novo* as well as reference-based *resequencing* projects to be carried out successfully [5,6]. Nevertheless, like other HTSTs, it cannot prescind from error modelling and correction strategies. They remain indispensable in order to compensate for the platform-specific sequencing artefacts and to tell them apart from natural variability.

In the pyrosequencing method, the greatest source of error corresponds to under or over-estimation of the number of incorporated bases at each cycle because of the noise in the luminescence signal. Actually, the signal intensity is translated into a numerical value (the flow value) which roughly corresponds to the number of incorporated bases. However this value can sometimes be ambiguous. For instance a flow value of 3.5 may corresponds to three or four incorporated bases? It is the job of the base caller program to take care of this uncertainty and call the correct number of bases. Base callers like PyroBayes [7] use Bayesian methods to calculate the maximum a posteriori (MAP) estimate for the number of incorporated bases *n* given a flow value *f*, using prior probabilities for the homopolymer lengths *P*(*n*) and the likelihood of the observed flow value given the homopolymer lengths, *P*(*f*|*n*). These latter values are estimated from alignments of test fragments to a reference sequence. PyroNoise [8] handles the problem of flowgram noise in a similar fashion but considering a data set of flowgrams (reads) as a whole. The method is based on a ‘distance’ that reflects the probability that a flowgram was generated by a given sequence. It then considers the set of flowgrams as being generated by a mixture model in which each component corresponds to a different sequence and the mixing coefficients indicate the relative abundance of the sequences in the set. Then it uses an EM algorithm (using latent variables that indicate which sequence gave rise to each flowgram) to estimate the model parameters, thus obtaining the de-noised set of sequences that most likely originated the flowgrams.

A few studies have been published which investigate the sequencing error characteristics of GS FLX data through the analysis of the sequence data. An early study by Huse et al. [9] assessed the quality of reads of a PCR amplicon library prepared from 43 reference templates containing rRNA genes produced with the GS20 technology. The observed overall error rate was of 0.49%, of which over 60% were insertion or deletions (indels) and 16% substitutions. Errors were found to be evenly distributed within each read but not among reads, with a very small percentage of low quality reads accounting for a large fraction of the errors. This analysis was challenged on a recent study by Gilles et al. [10] which considered reads obtained from control DNA fragments over three runs of the GS FLX Titanium protocol. They analysed the effect of several variables on the error rate distribution, including read length, the position of the error within the read, homopolymer effects, and the physical position of the bead on the PicoTiterPlate. Not only the mean error rate was higher than the one reported by Huse et al., 1.07%, but the conclusions also diverged in many important ways. Gilles and collaborators observed that virtually 90% of the reads contained errors (as opposed to only 18% observed by Huse and coauthors) and that the errors were nonrandomly distributed within these reads, with a degradation towards the 3’ end. They observed a significant effect of the other variables but found that none of them alone accounted for the error rate distribution, suggesting that the variation in the error rate resulted from higher order interactions of the explanatory variables.

High-throughput pyrosequencing error characteristics have also been studied with the aim of producing more realistic simulators for this kind of data. Balzer et al. [11] analysed a data set of bacterial sequences to derive an empirical model for the error distribution. By aligning the (trimmed and quality-filtered) reads to the reference genomic sequences, they could associate flowgram values to actual homopolymers of length up to 5 and fit these values to Gaussian distributions. The noise in the flowgram values is accounted for by the variance of these distributions. This sample variance was found to increase with the homopolymer lenght but also, for the same length, with the position within the read, confirming the findings of Gilles et al. This lead to the development of a flowgram simulator using ten different empirical distributions corresponding to adjacent windows covering 200 flow cycles. A refinement of this simulator was presented in a subsequent paper [12], in which Balzer et al. simulate PCR amplification errors at a rate estimated by analysing ‘sub- peaks’ of flowgram values over the integer values adjacent to the correct value. McElroy et al. [13] propose a simulator capable of generating sequences akin to GS FLX reads. In order to do so, the software uses the result of the alignment of a set of reads to a reference genome in order to build an empirical model for the errors. This model takes into account the nucleotide type, the position in the read, and the sequence context in which the errors occur by considering the three bases preceding the error and the base following the error. The analysis of a 454 data set containing aligned plasmid control sequences revealed average error rate figures inline with previous studies, but with a very limited positional effect. Homopolymer effects were also reported based on the analysis of the most common sequence error contexts.

Despite the aforementioned efforts to understand the error distributions of GS FLX data, no model is yet established as a standard. Application-specific error models are generally employed for some flexibility in the use of the reads, whether in de novo or resequencing applications. This usually amounts to scoring local alignments between a read and some other reads in the data set or a reference sequence, allowing for mismatches, insertions and deletions according to certain penalty schemes. A read may be classified as erroneous (and hence discarded) if its best alignment score deviates significantly from an exact match score. Most read alignment algorithms use an affine error model which penalises insertion or deletion (indel) gaps by attributing a fixed cost to a gap initiation and then a fixed cost to each additional gap position. This approach is simple to interpret and benefits from efficient quadratic-time dynamic programming implementations [14] but, due to the nature of pyrosequencing, it is debatable whether this uniformity is faithful to the underlying phenomena. Indeed, most studies seem to confirm that errors in long homopolymers are more common and so should be less heavily penalised. One could argue whether a convex penalty model, in which gap costs increase, for instance, according to a logarithmic function, would me more appropriate. Such models have already been successfully shown to fit biological sequence alignment data [15] and sub-cubic implementations of the corresponding optimal alignment algorithm exist [16].

In this study, we aimed at analysing the quality of the reads produced by the GS FLX instruments. Apart from assessing the overall sequencing error rates, our specific objective was to test whether the ubiquitous affine model is suitable for modelling high-throughput pyrosequencing errors, in comparison with a convex error saturation model used, for instance, in protein sequence alignment. Likewise, we analyse whether the length of the gaps could be influenced by the length and composition of the homopolymer in which they occur, which could motivate the use of a more specific model. Our procedure uses plain sequence data and can be used by users of most read mappers to estimate sensible values for the parameters corresponding to the costs of errors of various kinds.

## Methods

The problem of modelling the errors of a specific sequencing platform can be approached at many different levels of abstraction since each step of the process is prone to introduce errors. In the GS FLX automated sequencing-by-synthesis procedure, the prevalent type of error relates to the size of homopolymers. These errors can occur, for instance, because of an insufficient supply of free dNTPs at a given cycle, leading to an incomplete extension of a homopolymer stretch. On top of those basic chemical-level issues, there is the problem of capturing and interpreting the weak and noisy luminescent signals to determine the exact number of incorporated bases, which becomes particularly hard as the length of the homopolymer grows. Taking into account errors at the lowest levels requires comprehensive and specialised knowledge and control over the process which are usually inaccessible to end users and even bioinformatics specialists. Typically the lowest level data available are the SFF files containing the flowgrams. Some methods approach the issue of sequencing errors at this level by analysing data ‘in the flowspace’ [7,8,11,12].

The GS FLX pipeline includes conservative filters to rule out ambiguous spots, which usually results in a significant decrease in the number of useable reads, from about 1.6 million to a few hundred thousands. Despite that, one can still expect some errors to make it through to the sequence level, specially in genomes containing long homopolymers. Here we propose to tackle the problem at this level, which makes the method more universally accessible, even when the flowgrams are not available. Conceptually, our approach consists in comparing the reads produced by the instrument with the actual template sequences to count the number of sequencing errors of a few distinct types, and then fitting the values to an empirical model that reflects some basic knowledge about the ‘mechanics’ of the sequencing procedure.

### Data preparation

An important step when building an empirical model for a given phenomenon is to obtain a data set of faithful and descriptive observations of a sufficiently large number of its occurrences. In our case, and at the abstraction level we work, i.e. the sequence level, the ideal data set would be composed of alignments between the template sequences and the obtained reads which could characterise each reading error event having occurred. For example, an alignment AGGCTAG−CC would tell us that the first and fourth bases of the template, respectively A and C, were correctly read, whereas the two consecutive G’s were misinterpreted as one single copy and the final T was misread as C. In practice, though, we usually have only the readouts produced by the machine (maybe at different detail levels: raw image, signal intensity, sequence). We thus need to somehow infer the most plausible alignments from the available data, using also some knowledge about the sequenced samples and the underlying sequencing technology.

**Figure 1:**
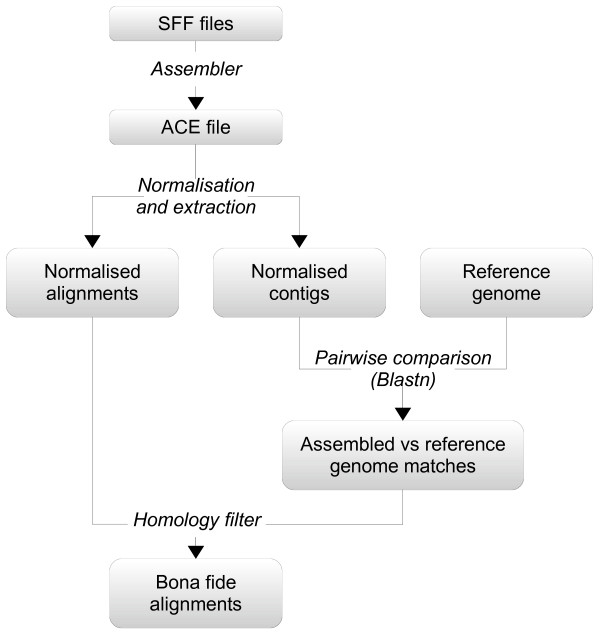
**Data set construction workflow.** Data set construction workflow.

Our data set construction strategy, depicted in Figure 1, stands upon two concepts: redundancy (coverage) and homology (conservation). Starting with the flowgram files (SFF) produced by the sequencer, we use an automatic *de novo* genome assembler to reconstruct the original genome. This results in an ACE file that describes how a certain number of contigs are formed from a patchwork of the original reads (or pieces of them). During the assembly, a minimum overlap is required between reads so that they can be stitched together. Consequently, every position of a contig is ‘covered’ by multiple overlapping reads. This redundancy is usually regarded as an indicator of the veracity of the assembled contigs, the idea being that errors eventually introduced in some reads are unlikely to be consistently reproduced over the other reads covering the same region.

At this point, we have an initial collection of matches of reads against the assembled contigs. Concretely, from the ACE file we extract a set of alignments {(*C*_*i*_,*R*_*j*_)} between pieces of contigs, *C*_*i*_, and reads, or pieces of reads, *R*_*j*_. As part of the assembly process, the reads can be padded so that they fit together, meaning that some positions of a given *R*_*j*_ or *C*_*i*_ may actually be gaps ‘-’. We then distinguish four cases concerning individual columns of an alignment as follows. An alignment column xy, for *x*,*y*∈{,,,} means that the base *x* at that position was read as *y*. If *x*=*y* then no error occurred, whereas if *x*≠*y* then we have a *substitution error*. An alignment column $\frac{x}{-}$ means that the base *x* of the contig (template) was skipped, i.e., not sequenced. We call this a *deletion error*. Similarly, an alignment column −y means that *y* was introduced by error in the read. We call this an *insertion error*. Finally, we can have an ill-defined column −−, which may happen because, as said, the template is covered by multiple reads and we can have some of those reads with an insertion error at a given position and the others without this error. Nevertheless, these latter will also have a ‘-’ padded at that position in order to comply with the former. For example, in the situation

$$\begin{array}{lll} \text{Contig}: & \;\text{AAGGCC}-\text{GTTGCGGC}\; & \;\\ \text{R1}: & \;\text{AAGGCC}-\text{GTT}\; & \;\\ \text{R2}: & \;\;\text{GGCC}-\text{GTTGCG}\; & \;\\ \text{R3}: & \;\;\;\;\text{CCCGTTGCGGC}\; & \;\\ & \; \end{array}$$

the read R3 has an extra C at its third position with respect to the other reads, and so a ‘-’ is inserted in the corresponding positions of the other reads. To correct this, we need to *normalise* the pairwise read-contig alignments, which essentially corresponds to removing these gap-only columns. After this step, we have a set of normalised alignments $\left\{(C_{i}^{\prime },R_{j}^{\prime })\right\}$.

Although a reasonable level of redundancy (coverage) confers some credibility to the obtained contigs, we take a further step to ensure that they do correspond to the actual sequences of the template molecules before taking their alignments into account. For this purpose, we look for homologous regions in the genome G of a closely related species, which we take as input. For each normalised alignment $\left(C_{i}^{\prime },R_{j}^{\prime }\right)$, we use BLASTN to find matches of $C_{i}^{\prime }$ in G, using minimum identity and maximum e-value cutoffs of *I*_min_ and *E*_max_, to determine whether the hit corresponds to a sufficiently conserved region to be trusted. If $C_{i}^{\prime }$ passes this homology filter, then we regard $\left(C_{i}^{\prime },R_{j}^{\prime }\right)$ as a *bone fide* alignment. To summarise, our contention is that, if the obtained contigs are homologous to sequences of a close, independently sequenced genome, then they must be real. Moreover, since they result from a multiple alignment of reads, then these alignments, and hence the sequencing events they represent, are also assumed to be faithful.

### Model evaluation

Two desirable (and possibly conflicting) properties of an empirical error model are first, that the model be capable of explaining well the observed errors and, second, that the model be simple enough to be useful in practice. The first property usually requires some kind of intuition and basic knowledge about the underlying process so that the model can be crafted to capture its essential characteristics. However, the more information we add to the model, the more likely it is to become too complex and to overfit the data. Ideally, we would like to keep it simple enough so as to have convenient analytical and computational properties, which may imply using known functional forms even at the cost of some accuracy.

It has already been reported that the most common sequencing errors in the GS FLX platform are insertions and deletions, with substitutions being much less frequent [9]. To assess the relative abundance of the different insertion and deletion (indel) gap lengths, we extracted the length of each observed gap from the alignment data and adjusted these values to two model distributions as follows.

In the first model, we assume that each position contributes equally to the overall probability of the gap. Put another way, the ratio between the probabilities of two consecutive gap lengths is constant. This is what is assumed by many tools (e.g. [17,18]), which use linear (or affine) functions to score the alignments. In this model, the probability for an observed gap to have length exactly *k*=1,2,… is given by

(1) $$P_{G}[X=k;\beta ]=\beta^{k-1}(1-\beta ),$$

where 0≤*β*≤1 is the contribution of each position. Since (1) is a geometric distribution, we refer to this as the *geometric model*.

To contrast with the widespread geometric model, we considered fitting the data to a Zipfian distribution, which has the form

(2) $$P_{Z}[X=k;\sigma ]=\frac{1/k^{\sigma }}{\overset{L}{\underset{j=1}{\sum }}1/j^{\sigma }}=Ck^{-\sigma },$$

where *σ*>0 is the parameter that controls the shape, *L* is set to the maximum gap length and $C={\left(\overset{L}{\underset{j=1}{\sum }}1/j^{\sigma }\right)}^{-1}$ is a normalising constant. The Zipfian distribution has already been successfully employed to model indel gap lengths in other contexts [15] and it is used to derive convex scoring functions for the alignments [19].

In order to compare the adequacy of the two models, we obtain maximum likelihood estimators for each of them with respect to the gap length data **x**=(*x*_1_,…,*x*_*N*_), that is, $\hat{\beta }(x)$ and $\hat{\sigma }(x)$. We then measure the overall discrepancy between the observed frequencies of each gap length and its maximum likelihood prediction by computing the Pearson’s *χ*^2^ statistic

(3) $$\chi^{2}(x)=\overset{L}{\underset{k=1}{\sum }}{\left(O_{k}-E_{k}\right)}^{2}/E_{k}$$

where *O*_*k*_ is the observed number of gaps of length *k* and *E*_*k*_ is its expected number of occurrences, given by $N\cdot P[k;\hat{\beta }]$ and $N\cdot P[k;\hat{\sigma }],$ for the geometric and Zipfian models respectively. The bigger the value of *χ*^2^(**x**) the poorer the fit, since this value is a sum of squared residuals.

The indel errors in the massively parallel pyrosequencing technique arise mainly due to the lack of precision in the conversion of the luminescence signal. It has been reported that this tends to be aggravated as the length of the homopolymer corresponding to the signal grows since a linear correspondence between signal intensity and sequence length cannot be assured beyond a certain number of bases [5]. To check to what extent this is indeed a problem in practice, we separate the gaps by homopolymer *context*. We define the context of a gap as follows. Let us consider the general form of a homopolymer deletion gap (the insertion case is analogous), that is

$$\begin{array}{l} \cdots x_{i}\cdots x_{j}\;x_{j+1}\cdots x_{k}\;x_{k+1}\cdots x_{s}\cdots \\ \cdots y_{i}\cdots y_{j}\;-\;\cdots \;-\;y_{k+1}\cdots y_{s}\cdots \end{array},$$

where the sequence of identical non-gap symbols *x*_*j*+1_=⋯=*x*_*k*_=*α* have been deleted. The left flank of the context of this deletion is defined as the *maximal* sequence of matches with *x*_*i*_=*y*_*i*_=⋯=*x*_*j*_=*y*_*j*_=*α*. Similarly, the right flank of the context is defined as the maximal sequence of matches with *x*_*k*+1_=*y*_*k*+1_=⋯=*x*_*s*_=*y*_*s*_=*α*. Notice that either part may be empty. Then, the overall context of this gap of lengh *k*−*j* is the homopolymer *x*_*i*_⋯*x*_*s*_. Hence, for example, in the alignment AACCCCCAATG−TAT−AC−−CC−−−GGTG−, the gap contexts, from left to right, will be AA, CCCCC, AA, T, GG and T.

We perform two-sample Mann-Whitney tests for each pair of contexts to determine if their gap lengths come from the same distribution. Incidentally, these tests also serve to determine if there are significant differences in the gap lengths according to the composition of the contexts.

## Results and discussion

We illustrate this discussion with the results of the analyses performed on three data sets, summarised in Table 1. To avoid an eventual instrument-specific bias, we used data obtained from different sites. The MH sequencing was carried out at the LNCC, in Brazil. The SA and SP reads were obtained from the SRA, as indicated in Table 1, the sequencing data being provided by the WUGSC and the JCVI, respectively, both in the USA. Since we were interested in assessing the error characteristics of the GS FLX platform, our analyses considered the assemblies produced by the GS *De Novo* Assembler [4], commonly referred to as Newbler, which is provided as the ‘official’ platform software. Because of the alignment data construction strategy discussed above and illustrated in Figure 1, the analysis will be, in principle, assembler-specific. The fact that we use only assembled fragments that match a reference sequence obtained independently will hopefully mitigate this influence of the assembler tool. However, in order to check the robustness of our conclusions, we also performed the analyses with the Celera WGS assembler, which has been successfully adapted to work with GS FLX data [20]. All assemblies were produced with Newbler version 2.3 and Celera WGS assembler version 7.0 with default parameters.

**Table 1:** Data sets

**Data id.**	**Sequenced organism**	**Reference organism**	**Nb. of reads**	**Avg. read length**
	**(Data source)**	**(Data source)**		
MH	*M.hyopneumoniae* 7422	*M.hyopneumoniae* 7448	83,084	247nt
	(LNCC - Additional file 1)	(GenBank: NC_007332)		
SA	*S.aureus* USA300	*S.aureus* MSHR1132	267,970	269nt
	(SRA: SRR000892)	(Genbank: NC_016941.1)		
SP	*S.pneumoniae* CDC1873-00	*S.pneumoniae* G54	169,176	262nt
	(SRA: SRR001327)	(Genbank: NC_011072.1)		

Data sets used in this study. We use the shorthand ids MH for *Mycoplasma hyopneumoniae*, SA for *Staphylococcus aureus* and SP for *Streptococcus pneumoniae*.

The results of the assembly step are summarised in Table 2. Although the overall assembly sizes are very similar, we see that Newbler consistently produced fewer contigs. Not only it yielded less contigs, but the N50/N80 statistics also favour Newbler over the WGS-assembler. That said, we note that those global properties of the assemblies are only of relative importance to our analysis because we care only about the local alignment information. The ACE files containing the automatic assemblies were processed according to the pipeline described in ‘Methods’, with *I*_min_ and *E*_max_ set to 0.8 and 0.01 respectively. The number and average length of the extracted alignments, both the intermediate candidate alignments, obtained after normalisation, and the final *bona fide* ones, are shown in Table 3. For the analyses, we pooled together all these data, resulting in an a global data set of 332,189 alignments spanning 82,837,778 positions for Newbler, and 259,384 alignments spanning 70,835,968 positions for the WGS assembler.

**Table 2:** Assembly data

**Data id.**	**Assembler**	**Nb. of**	**Assembly**	**N50 / N80**
		**contigs**	**size**	
MH	Newbler	102	981,106	27,323 / 10,189
	Celera	282	1,011,888	10,963 / 4,287
SA	Newbler	66	2,971,290	143,335 / 56,013
	Celera	691	3,162,318	52,376 / 17,400
SP	Newbler	178	2,223,061	30,556 / 18,166
	Celera	671	2,331,216	15,133 / 6,442

Genome assemblies data summary. ‘Assembly size’ corresponds to the overall sum of the lengths of the contigs. The N50 (respectively N80) value corresponds to the largest contig length *L* such that the contigs of length ≥*L* contain at least 50% (resp. 80%) of the bases in the assembly.

**Table 3:** Alignment data summary

**Data id**	**MH**		**SA**		**SP**	
**Assembler**	**Newbler**	**Celera**	**Newbler**	**Celera**	**Newbler**	**Celera**
Nb. of candidate alignments	84,376	77,058	269,693	209,626	172,436	119,367
Cand. alignments avg. length	242	299	257	266	242	259
Nb. of bona fide alignments	79,135	73,535	108,718	84,692	144,336	101,157
Bona fide alignments avg. length	245	300	258	266	245	259

Alignment data characteristics for *I*_min_ = 0.8, *E*_max_ = 0.01.

First, we examine the overall characteristics of the extracted errors. Table 4 summarizes the exact match occurrences. We group these occurrences by homopolymers since the actual sequencing occurs one homopolymer at a time. We include here only exact matches between homopolymers that are not in the context of a gap (see Methods). We notice that the matches concentrate on homopolymers of length up to 7-8, not only because they are easier to sequence but mainly because they are much more abundant. For Newbler, the exact match alignments cover 97.78% of the total aligned positions, and for the Celera assembler, 93.52%, which indicates a very low sequencing error rate in general, and in particular when the official assembler is used, a testimony of the very conservative filters applied by this platform.

**Table 4:** Homopolymer exact match occurrences

**Newbler**												
	**1**	**2**	**3**	**4**	**5**	**6**	**7**	**8**	**9**	**10**	**11**	**12**
A	10112299	3780060	1394445	591882	250066	94484	27007	4569	498	61	10	9
C	9275676	1932907	270496	54226	8510	1239	175	6	0	0	0	0
G	9325002	1918715	273077	51062	7796	1026	115	10	0	0	0	0
T	10114600	3747686	1369516	584450	260939	101704	33958	6960	802	88	37	18
	**13**	**14**	**15**	**16**	**17**	**18**	**19**	**20**	**21**	**22**	**23**	**24**
A	10	4	3	7	1	2	0	1	0	0	0	1
C	0	0	0	0	0	0	0	0	0	0	0	0
G	0	0	0	0	0	0	0	0	0	0	0	0
T	21	11	4	2	4	1	0	2	0	0	0	0
*Total match positions: 80,998,946*												
**Celera**												
	**1**	**2**	**3**	**4**	**5**	**6**	**7**	**8**	**9**	**10**	**11**	**12**
A	8266460	3097760	1128372	492925	215277	82702	26304	4552	651	79	38	24
C	7527051	1547142	217848	43078	6786	876	113	0	0	0	0	0
G	7443144	1495949	212552	41896	6191	882	147	20	0	0	0	0
T	8322275	3084911	1146793	502731	224735	89882	29349	5634	423	55	32	25
	**13**	**14**	**15**	**16**	**17**	**18**	**19**	**20**	**21**	**22**		
A	15	12	16	5	1	1	3	0	0	1		
C	0	0	0	0	0	0	0	0	0	0		
G	0	0	0	0	0	0	0	0	0	0		
T	48	12	11	8	6	2	2	1	0	0		
*Total match positions: 66,248,219*												

Homopolymer exact match occurrences. The row indicates the base of the homopolymer and the column indicates its length. Thus for example, there were 7796 exact match alignments of GGGGG in the Newbler data set, and only 111 of TTTTTTTTTTTTTTT in the Celera assembler data set.

The substitution errors are summarised in Table 5. We have observed 665,969 substitutions for Newbler which account for only 0.08% of the total positions. For the Celera assembler, this absolute proportion was significantly higher, at about 4.6%. This, combined with the observation of the alignment lengths of Table 3 indicates that the WGS assembler privileges longer reads and is more permissive as for substitutions, which is not surprising given its Sanger sequencing origins. With Newbler, although the substitution error rate is very low in absolute terms, if we regard the number of substitutions relative to the overall number of errors (including indels), we have that they account for about 50% of the total number of errors in this data set (665969/(665969+389063+287991)), which is quite significant, although in comparison this is a lower proportion than what is observed in other technologies [21]. With the Celera assembler, substitutions account for over 77% of the errors, again possibly due to its excessive tolerance towards mismatches. We observe a bias towards C/G substitutions relative to A/T (∼22*%* vs. ∼12*%* in Newbler, and ∼27*%* vs. ∼9*%* in Celera).

**Table 5:** Base substitutions

**Newbler**				
	**A**	**C**	**G**	**T**
A	0 (0%)	74588 (11.2%)	63823 (9.58%)	92787 (13.93%)
C	26505 (3.98%)	0 (0%)	49970 (7.5%)	41064 (6.17%)
G	23442 (3.52%)	48670 (7.31%)	0 (0%)	51326 (7.71%)
T	53587 (8.05%)	72148 (10.83%)	68059 (10.22%)	0 (%)
*Total substitutions: 665,969*				
**Celera**				
	**A**	**C**	**G**	**T**
A	0 (0%)	314368 (9.48%)	298435 (9%)	454647 (13.71%)
C	215248 (6.49%)	0 (0%)	155494 (4.69%)	220471 (6.65%)
G	214762 (6.48%)	155015 (4.67%)	0 (0%)	214063 (6.46%)
T	449989 (13.57%)	310634 (9.37%)	312819 (9.43%)	0 (0%)
*Total substitutions: 3,315,945*				

The cell at row *x* and column *y* indicates the number of occurrences of the alignment column xy in the data set, that is, the number of times the base *x* was substituted by *y*. Close to this number, in parentheses, is the percentage of the value relative to the total number of substitutions.

**Figure 2:**
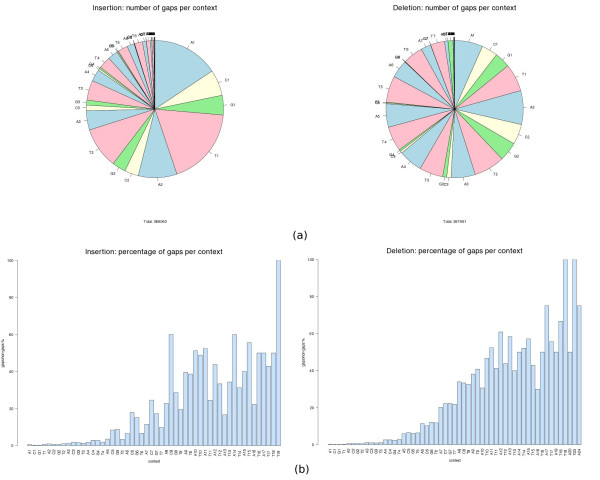
**Indel gaps per context. (a)** Absolute number of gaps per context (left: insertions, right: deletions). Contexts are represented in the form *αℓ*, where *α* indicates the base of the homopolymer and *ℓ* indicates its length (e.g. T5=TTTTT). **(b)** Ratio between the number occurrences of an homopolymer as a context of a gap and its number of exact match alignments (ln the *x*-axis, the contexts are listed in the order A1, C1, G1, T1, A2, C2, G2, T2,...).

Next, we examine the number of indel errors per context, which are illustrated in Figure 2(a). We notice that the absolute number of insertion gaps decreases with the size of the context, which is not surprising, given the relatively lower number of large contexts themselves. Indeed, if we compute the ratio between the number of occurrences of a given homopolymer as an indel context and the number of its exact match alignments, we observe a clear tendency for the chance of having a gap in a homopolymer to increase with its length, as can be seen in Figure 2(b). Data shown in Figure 2 concern the Newbler assembler. The equivalent graphics for the WGS assembler reveal qualitatively similar patterns and can be found in the Additional file 2.

**Figure 3:**
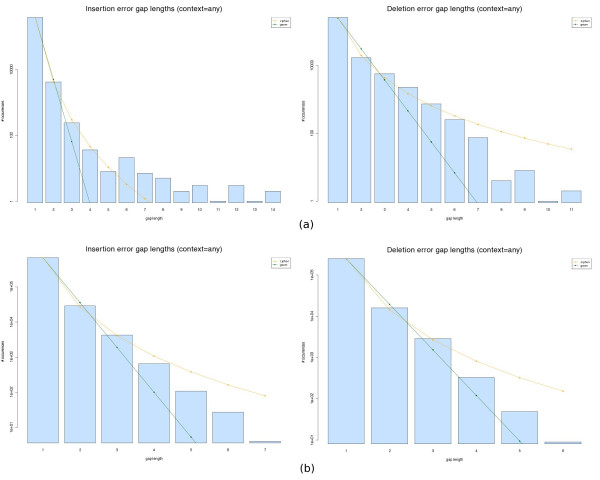
**Gap lengths distribution.** Histograms of gap lengths with any context (insertion gaps on the left, deletion gaps on the right) for the Newbler **(a)**, and Celera WGS assembler **(b)**. Also shown are the best fit approximations of these histograms using the geometric (green line) and Zipfian (orange line) models.

The distribution of gap lengths is illustrated in Figure 3, which shows the histograms of the lengths of insertion (left) and deletion (right) gaps for both Newbler (top) and Celera assembler (bottom). As it can be seen, there is a dominant abundance of gaps of size one and the histograms are somewhat evocative of a power-law distribution. We notice that the Celera gaps tend to be smaller in average, again in line with its Sanger sequencing origins.

**Figure 4:**
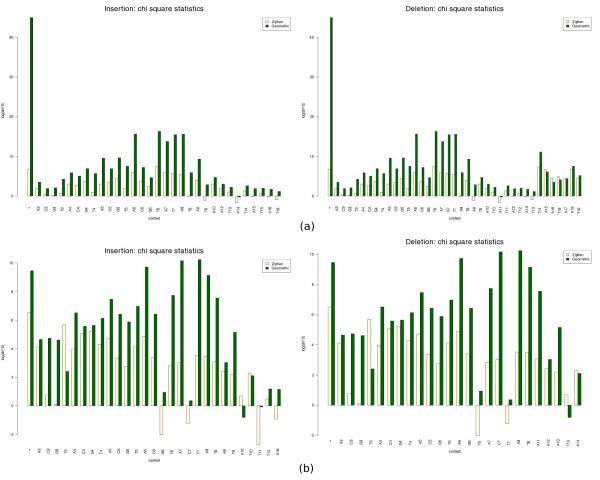
**Goodness of fit assessment.***χ*^2^ statistics of maximum likelihood fits per context (insertion gaps on the left, deletion gaps on the right) for the Newbler **(a)**, and WGS assembler data **(b)**. The first pair of bars in each graph correspond to any context (context=*). The remaining pairs of bars correspond to the contexts listed in the usual order A1, C1, G1, T1, A2, C2, G2, T2,...

We wanted to compare the approximations of the gap length data with Geometric and Zipfian models, as explained in Methods. In order to visually compare the fits of these models, we plotted the residuals in the two scenarios using both the Newbler and WGS assembler data. This information is summarised in Figure 4 which shows the value of the Pearson’s *χ*^2^ statistic for the maximum likelihood fits of the insertion and deletion gap lengths for each context. The graphics show that the Zipfian sums of squared residuals (yellow bars) are up to a few orders of magnitude smaller than the Geometric (green bars) ones (notice the log scale of the graph), thus evidencing the fact that the Zipfian approximation is much closer to the actual distribution of gap lengths for any context (which is also suggested by the best fit curves in Figure 3). However, despite this difference, the *χ*^2^ goodness-of-fit tests do not allow us to claim that the Zipfian is the right approximation, with P-values varying haphazardly depending on the context, and often being not significant enough to support the null hypothesis (see Additional files 2 and 3 for the full results).

**Figure 5:**
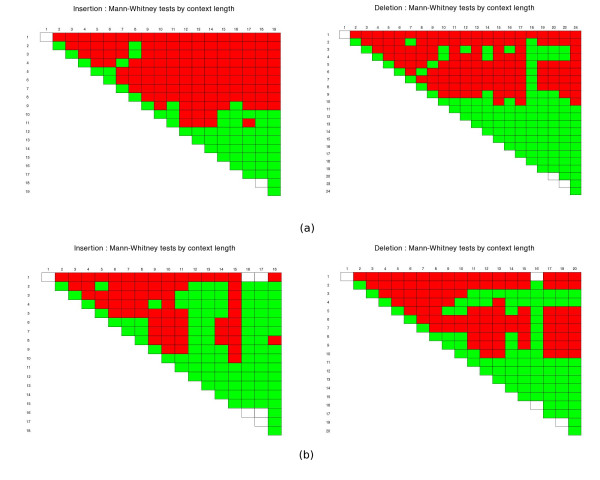
**Mann-Whitney tests for the gap length data.** Mann-Whitney tests for the gap length data per context length using Newbler **(a)** and WGS assembler data **(b)**. In each graphic, the cell (*i*,*j*) contains the result of the test of the null hypothesis *H*_0_: the gaps from contexts of lengths *i* and *j* come from the same distribution, against the alternative hypothesis *H*_1_: they do not come from the same distribution. The cell is shown in red if the null hypothesis is rejected at the significance level *α*=1*%*, or in green otherwise.

The next question to be addressed is whether there are significant changes in the gap length distributions according to the context. We tested whether the insertion and deletion gap lengths come from the same distribution for each pair of contexts using both the Newbler and WGS assembler data. To help us establish a pattern and to circumvent the lack of data which may render the test approximations imprecise, we summarised this information by performing the same kind of test, this time grouping the contexts by length. The graphics in Figure 5 display the results of these tests (for the full results, see Additional files 2 and 3) for every pairwise combination of insertion (left) or deletion(right) context length. In the cell with coordinates (*i*,*j*), we have the test of the null hypothesis that the insertion/deletion gap lengths with contexts of length *i* and *j* come from the same population, against the alternative hypothesis that they do not come at the significance level *α*=0.01 (two-tailed test). If the null hypothesis is rejected (indicating different distributions) the cell is filled in red. Otherwise, it is filled in green. A blank cell indicates that the test could not be performed, typically because of the lack of data (the main diagonals are, of course expected to be green). The results show a majority of red cells, particularly in the upper left parts of the triangles, which correspond to smaller contexts, for which the abundance of data makes the test more meaningful. In fact, if we consider only contexts of length up to 8 (longer contexts have very few occurrences), we have almost exclusively red cells. The lower parts of the triangles correspond to the cases in which the contexts are longer and hence the difference between their lengths relatively smaller, and for which there is much less data. These factors make the tests in those cases less accurate. Overall, these tests seem to confirm an influence of the context, in particular of its length to its corresponding gap lengths.

## Conclusion

We have presented an empirical study aimed at characterising the sequencing errors occurring in massively parallel pyrosequencing technology. We have devised a procedure to generate data sets of *bona fide* alignments in which these sequencing errors are represented as mismatches and gaps. We evaluated general patterns of incidence of the substitutions and indel errors, and more specifically the distribution of the indel lengths and the influence of the sequence context to this length. Inspired by previous work on biological sequence alignments [15], we have compared a geometric model of gap length distributions to a Zipfian model. The geometric model is assumed by many state-of-the-art read mapping tools, e.g. [17,18], which score gaps according to an affine function of their lengths. In these schemes, each additional gap position is penalised with a fixed amount. Our results suggest that this affine model is not the most realistic when it comes to pyrosequencing errors because it fails to approximate the observed gap lengths distribution and it disregards the sequence context. However, the Zipfian approximation, albeit more accurate, also failed to show a significantly better fit as to justify that we abandon the efficient dynamic programming local alignment procedures of the affine model in favour of more specialised and hence more computationally intensive gap penalty schemes. Our experiments have shown that the conservative filters applied by the GS FLX platform do enforce a high sequence quality, as demonstrated by the very low rates of error per base. We postulate, however that, for 454 pyrosequencing data, the procedure that we have implemented to carry out this study can still offer a viable alternative for the estimation of realistic parameters for alignment scoring functions in a principled manner, in contrast to arbitrary values often used by many tools in an *ad hoc* fashion.

## Availability of supporting data

Additional files, including supporting data and source code for the software used in this study (Additional file 1), as well as the complete results (Additional files 2 and 3), are available from our supporting website http://kdbio.inesc-id.pt/~pgsf/pyroseq\_err.

## Competing interests

The authors declare that they have no competing interests.

## Authors’ contributions

PF and AF conceived the study. JP, LA and AV produced the sequence data and genome assemblies. PF implemented the software and carried out the experiments. All authors have contributed to the analysis of the results. PF wrote the manuscript. All authors have revised, read and approved the manuscript.

## Supplementary Material

Additional file 1 Compressed archive containing the source code (in Shell script, Python and R) and the analysed data sets (ACE and FASTA files). Click here for file

Additional file 2 Full results of the experiments for the Celera WGS assembler. Click here for file

Additional file 3 Full results of the experiments for the Newbler assembler. Click here for file
