# Supplementary material for: Empirical assessment of sequencing errors for high throughput pyrosequencing data
Source: BMC Res Notes. 2013 Jan 22;6:25. doi: 10.1186/1756-0500-6-25 (PMC3852801; doi:10.1186/1756-0500-6-25)

celera Matches

HP base \ HP length	1	2	3	4	5	6	7	8	9	10	11	12	13	14	15	16	17	18	19	20	21	22
A	8266460	3097760	1128372	492925	215277	82702	26304	4552	651	79	38	24	15	12	16	5	1	1	3	0	0	1
C	7527051	1547142	217848	43078	6786	876	113	0	0	0	0	0	0	0	0	0	0	0	0	0	0	NA
G	7443144	1495949	212552	41896	6191	882	147	20	0	0	0	0	0	0	0	0	0	0	0	0	0	NA
T	8322275	3084911	1146793	502731	224735	89882	29349	5634	423	55	32	25	48	12	11	8	6	2	2	1	0	NA

Total HP matches: 45265873

Total match positions: 66248219

celera Substitutions

	A	C	G	T
A	0 (0 %)	314368 (9.48 %)	298435 (9 %)	454647 (13.71 %)
C	215248 (6.49 %)	0 (0 %)	155494 (4.69 %)	220471 (6.65 %)
G	214762 (6.48 %)	155015 (4.67 %)	0 (0 %)	214063 (6.46 %)
T	449989 (13.57 %)	310634 (9.37 %)	312819 (9.43 %)	0 (0 %)

Total substitutions: 3315945

celera Insertion

Insertion per context

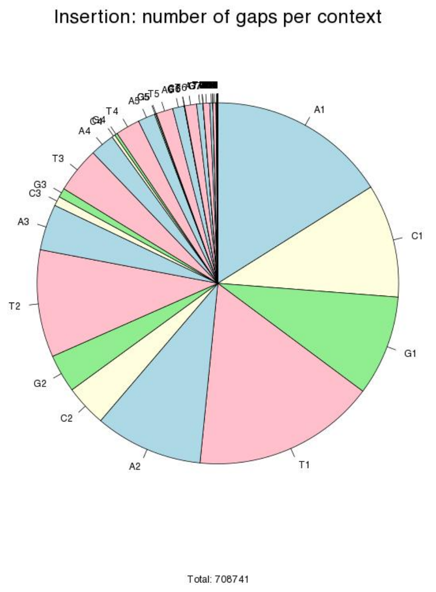

Insertion per context

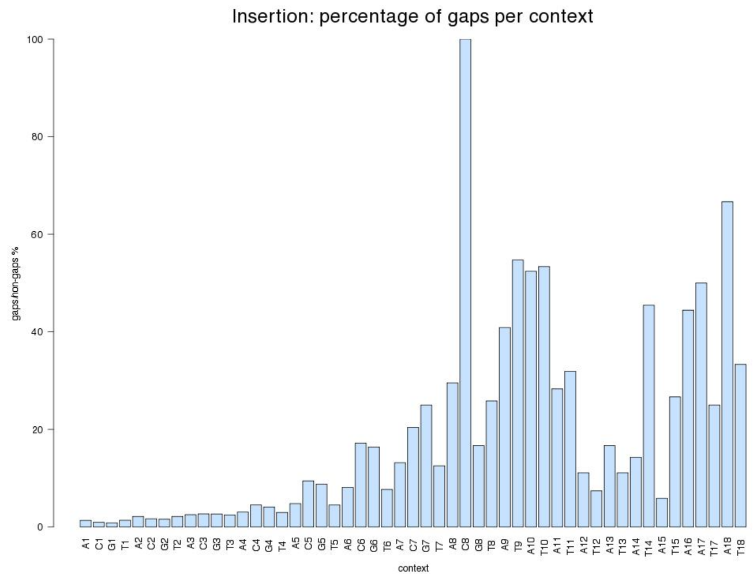

Context	#Data	Gap lengths	Zipfian fit	Geom fit
*	708741	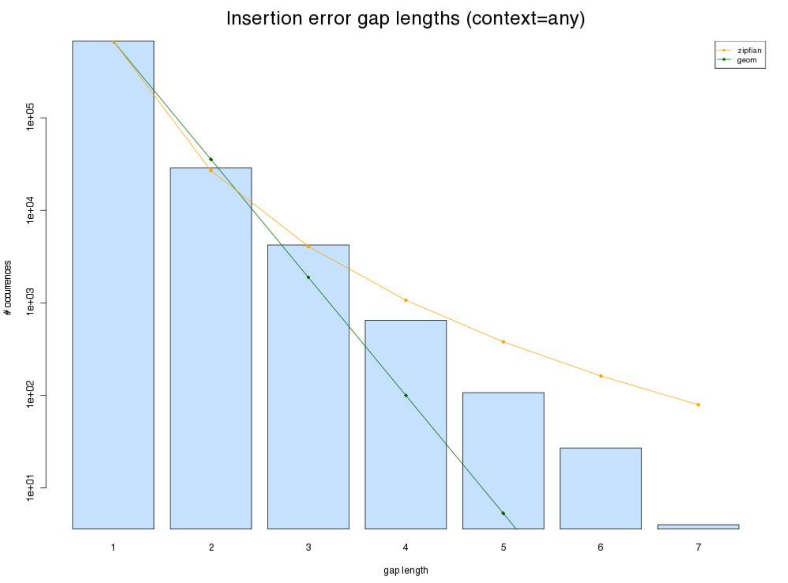	s = 4.65175134225846 X^2 = 674.385812543933 p-value = 1.69470235413899e-143	beta = 0.0530208184409083 X^2 = 12804.3549351305 p-value = 0
A1	113772	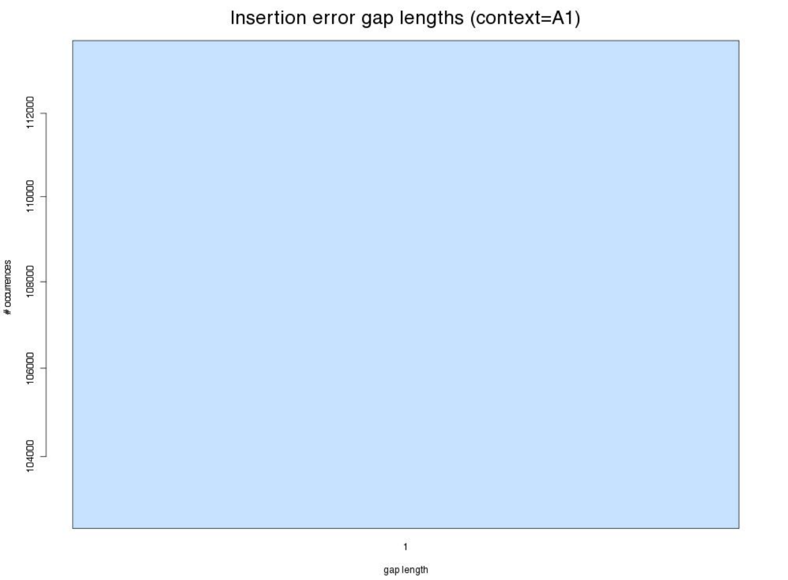	INVALID/ERROR	INVALID/ERROR

C1	72069	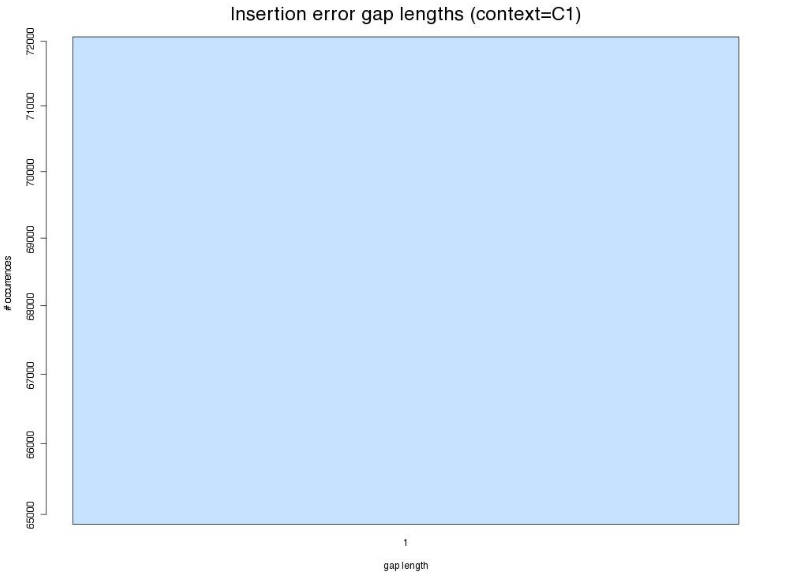	INVALID/ERROR	INVALID/ERROR
G1	63614	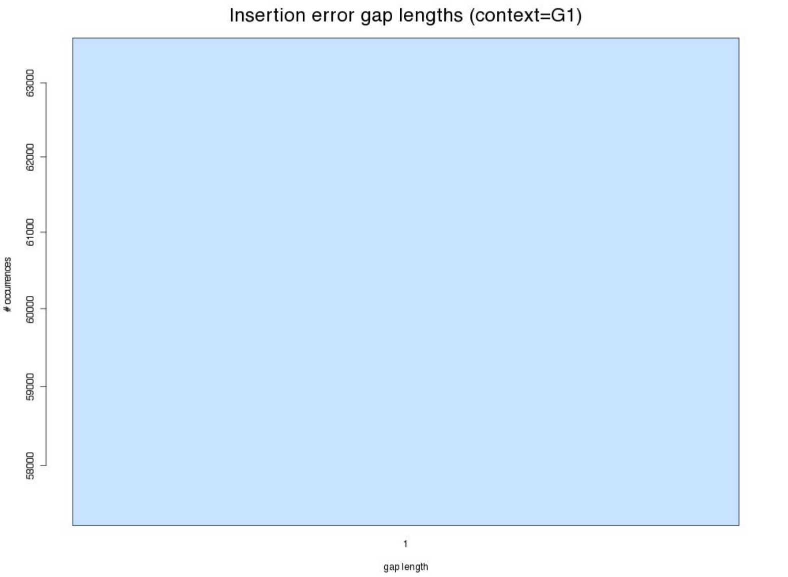	INVALID/ERROR	INVALID/ERROR
T1	116096		INVALID/ERROR	INVALID/ERROR

		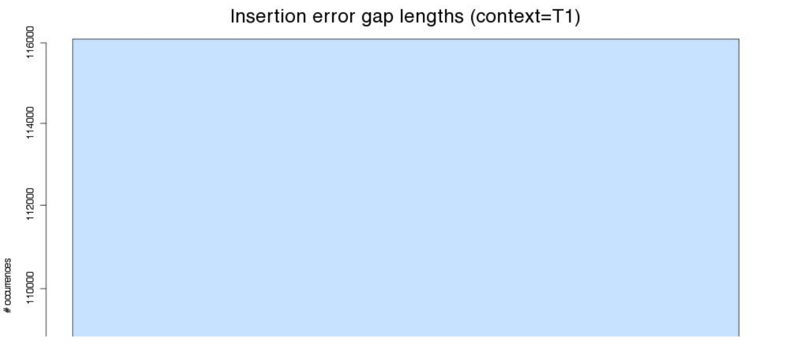		
A2	68446	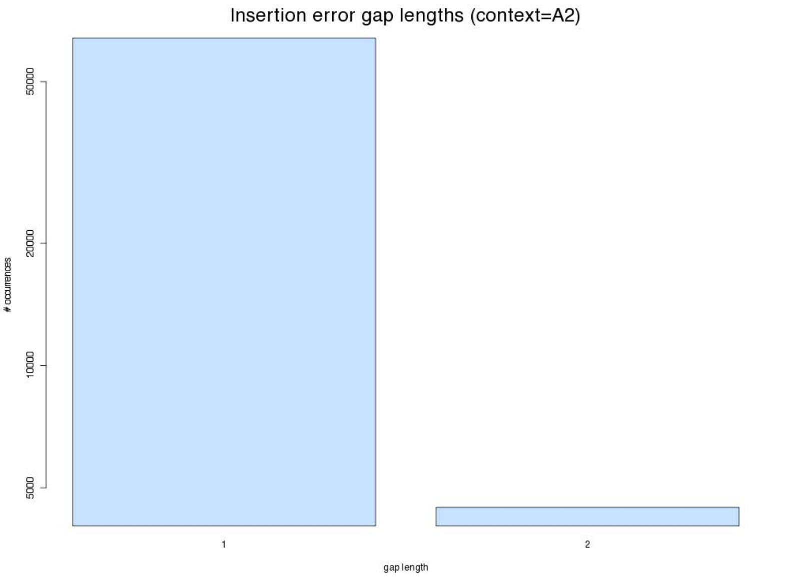	INVALID/ERROR	INVALID/ERROR
C2	26389	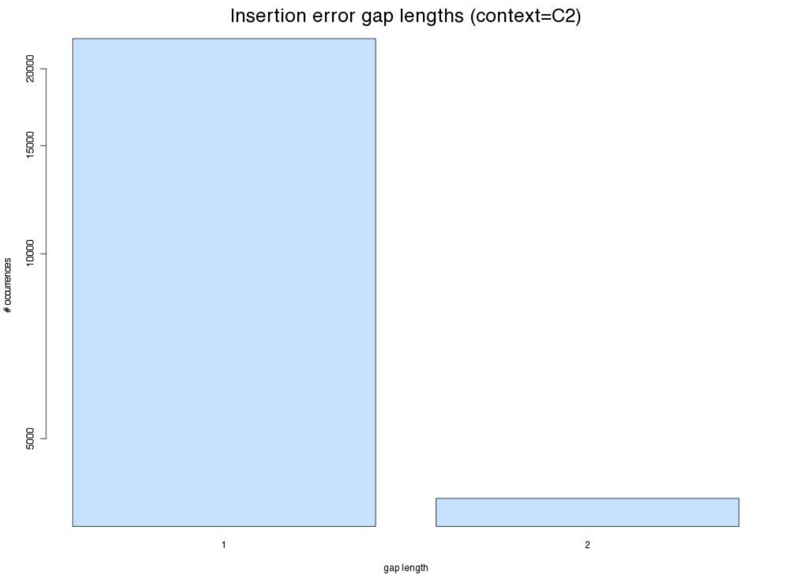	INVALID/ERROR	INVALID/ERROR

G2	24184	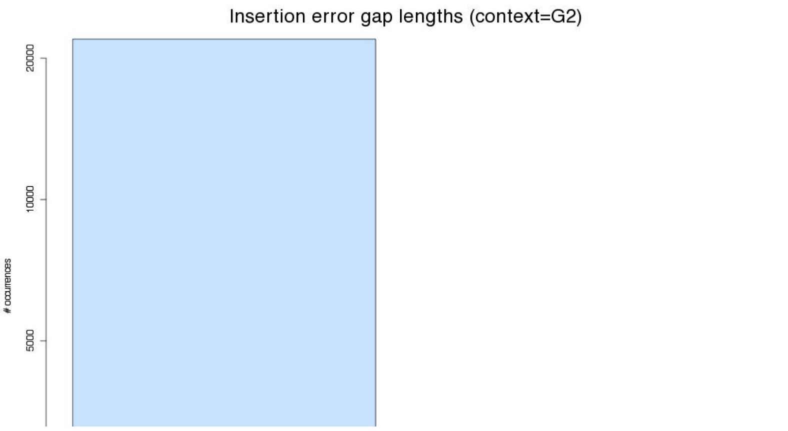	INVALID/ERROR	INVALID/ERROR
T2	68146	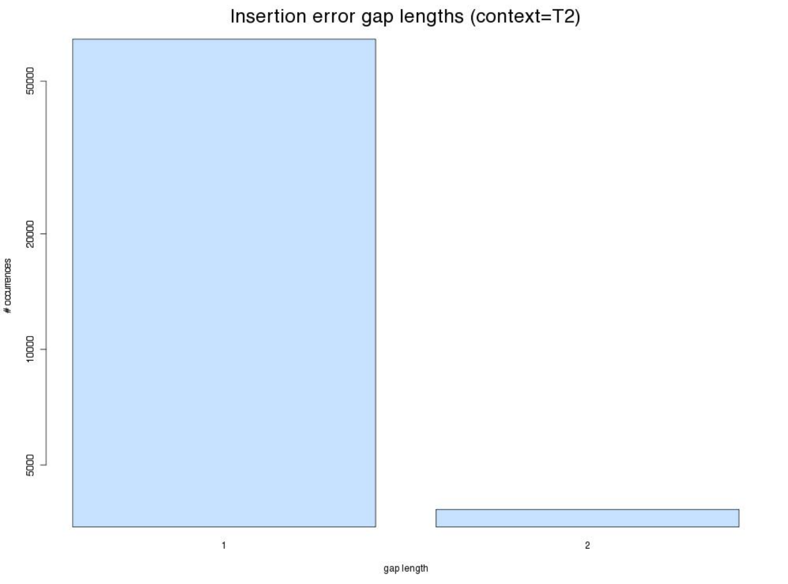	INVALID/ERROR	INVALID/ERROR
A3	29213	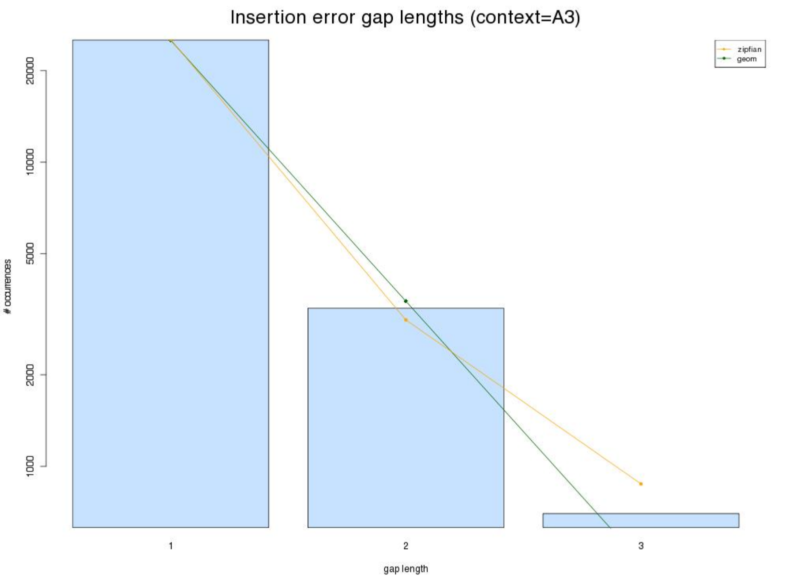	s = 3.06237840832514<br>X^2 =<br>61.671294560715<br>p-value =<br>4.05854092363824e-15	beta =<br>0.138767688679245<br>X^2 =<br>104.589672335044<br>p-value =<br>1.50237857823962e-24

C3	6007	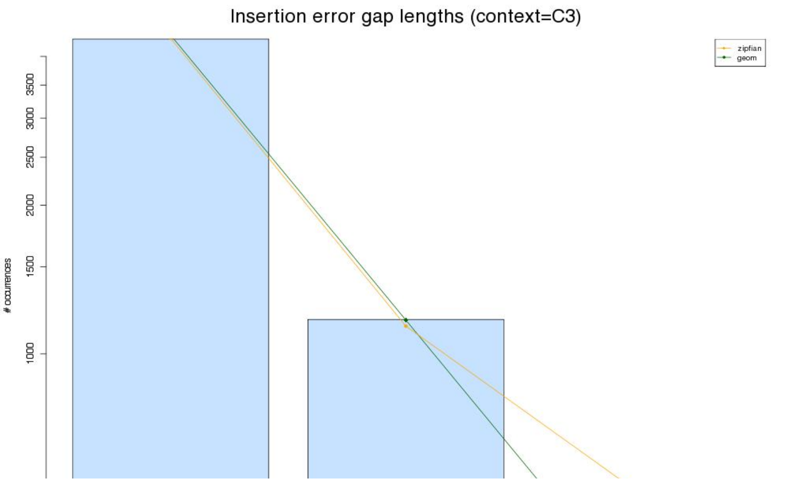	s = 1.93399959970195<br>X^2 =<br>2.1480204135818<br>p-value =<br>0.142753845472073	beta =<br>0.26519877675841<br>X^2 =<br>113.508507577056<br>p-value =<br>1.66963420626503e-26
G3	5805	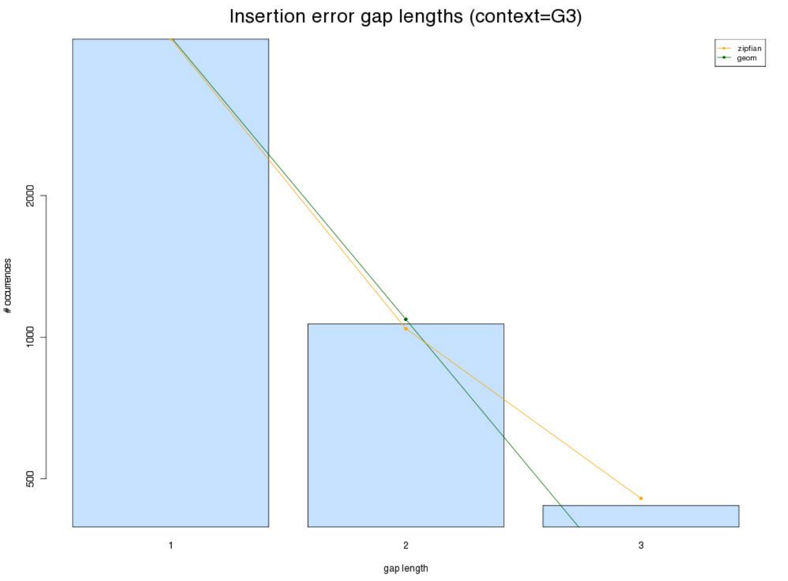	s = 2.04713324252887<br>X^2 =<br>1.12952914792824<br>p-value =<br>0.28787579475601	beta =<br>0.250967741935484<br>X^2 =<br>100.664699511723<br>p-value =<br>1.08950108883066e-23
T3	28987	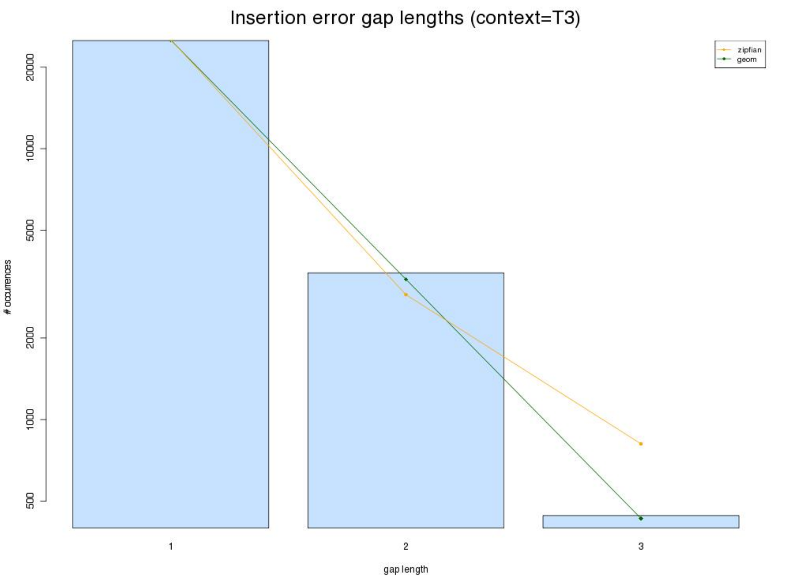	s = 3.12791393067888<br>X^2 =<br>291.525619548521<br>p-value =<br>2.31281332334304e-65	beta =<br>0.130876709042936<br>X^2 =<br>11.1617662576153<br>p-value =<br>0.000835003877040266

A4	15518	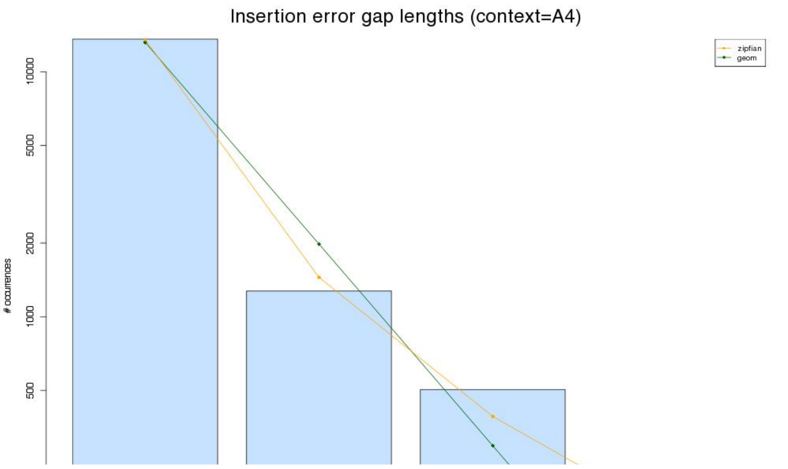	s = 3.2224395792105 X^2 = 53.2356871606835 p-value = 2.75433913544143e-12	beta = 0.150257365020261 X^2 = 674.475514779563 p-value = 3.46340178298326e-147
C4	2035	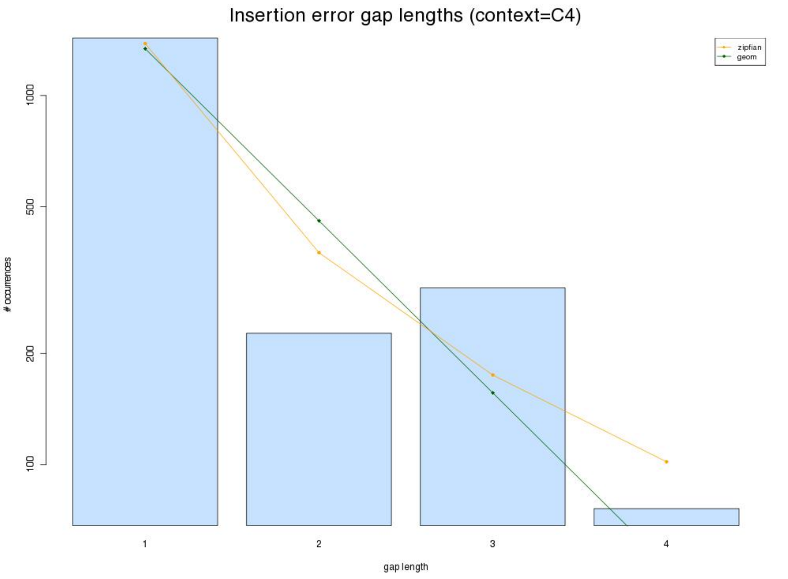	s = 1.88205039535727 X^2 = 157.599674325211 p-value = 5.99329348865111e-35	beta = 0.341849935316947 X^2 = 265.503174860792 p-value = 2.22186728843846e-58
G4	1790	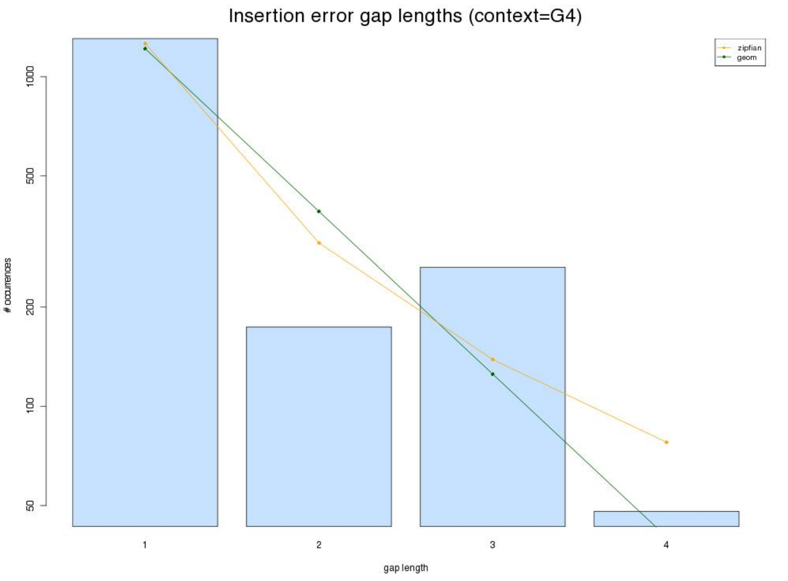	s = 2.00917566951851 X^2 = 188.053213499704 p-value = 1.46138126866772e-41	beta = 0.320940819423369 X^2 = 281.551127402893 p-value = 7.27695178872066e-62

T4	15430	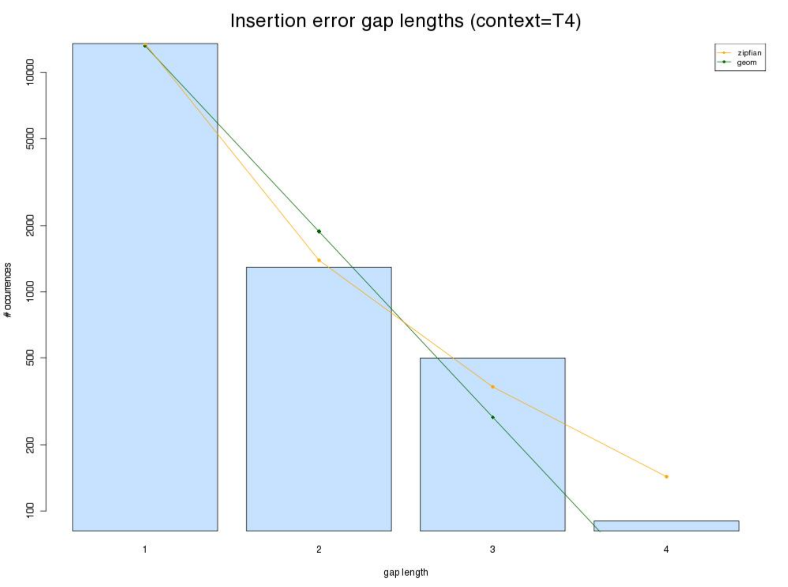	s = 3.28034822018251 X^2 = 72.6997252667011 p-value = 1.63476465902417e-16	beta = 0.142253599421869 X^2 = 460.690303241429 p-value = 9.17005030140118e-101
A5	10842	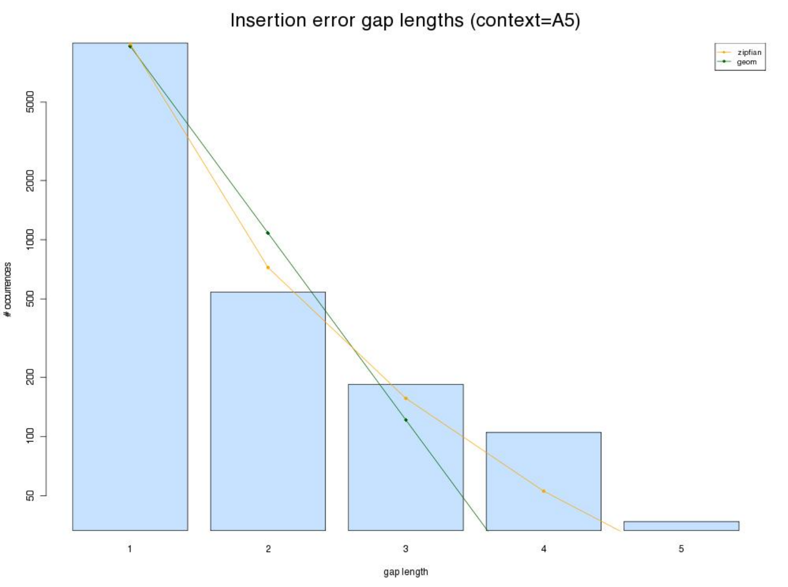	s = 3.77476284629817 X^2 = 111.837818667721 p-value = 4.41360141639958e-24	beta = 0.112330112985099 X^2 = 1747.64936377591 p-value = 0
C5	708		s = 4.45616172991951 X^2 = 28.5552989337661 p-value = 2.77689349096759e-06	beta = 0.072083879423329 X^2 = 616.653919890878 p-value = 2.4715309546673e-133

		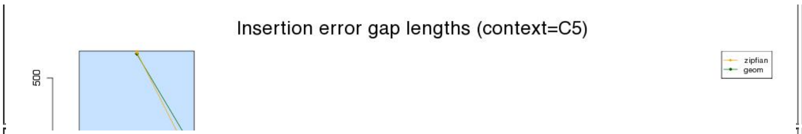		
G5	597	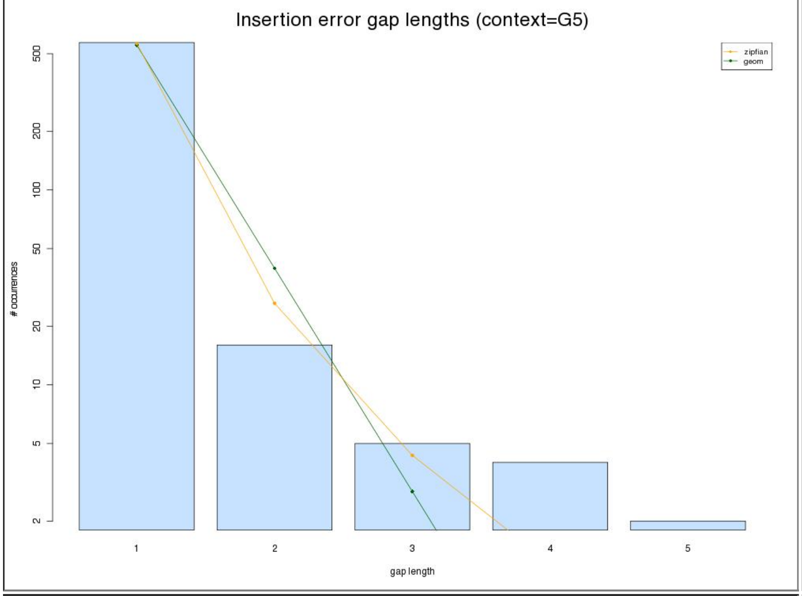	s = 4.430144643528<br>X^2 =<br>15.7950257056116<br>p-value =<br>0.00124915403237366	beta =<br>0.0715396578538103<br>X^2 =<br>358.769367985303<br>p-value =<br>1.88267273876526e-77
T5	10593	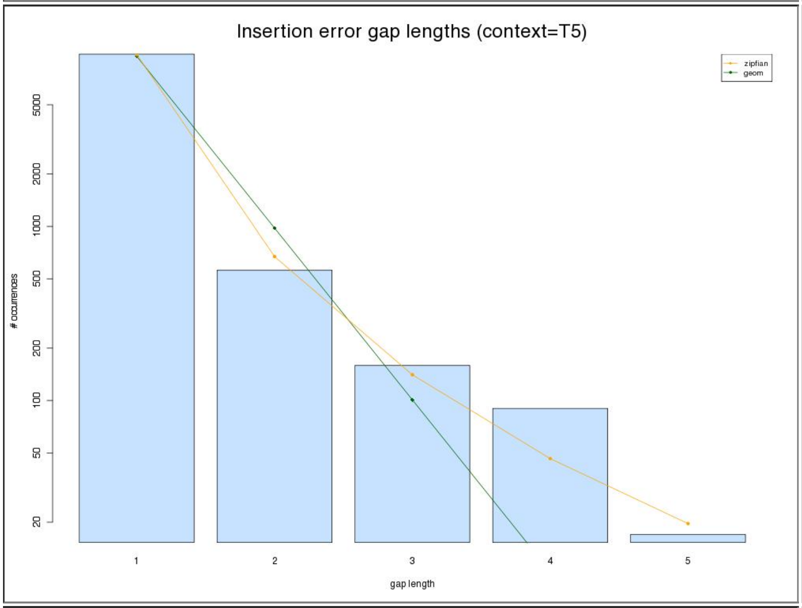	s = 3.85523658278546<br>X^2 =<br>62.1534232073567<br>p-value =<br>2.03729708512252e-<br>13	beta =<br>0.103048264182896<br>X^2 =<br>1065.64902268110<br>p-value =<br>1.03135701472478e-<br>230
A6	7311	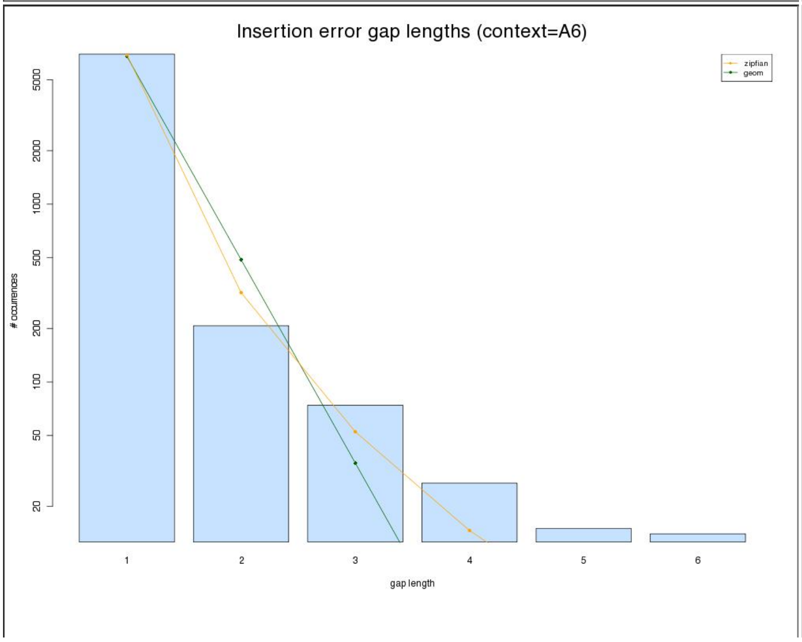	s = 4.44310277230933<br>X^2 =<br>131.050922936449<br>p-value =<br>2.32082052151719e-<br>27	beta =<br>0.0718547670432905<br>X^2 =<br>16713.6120500416<br>p-value = 0

C6	187	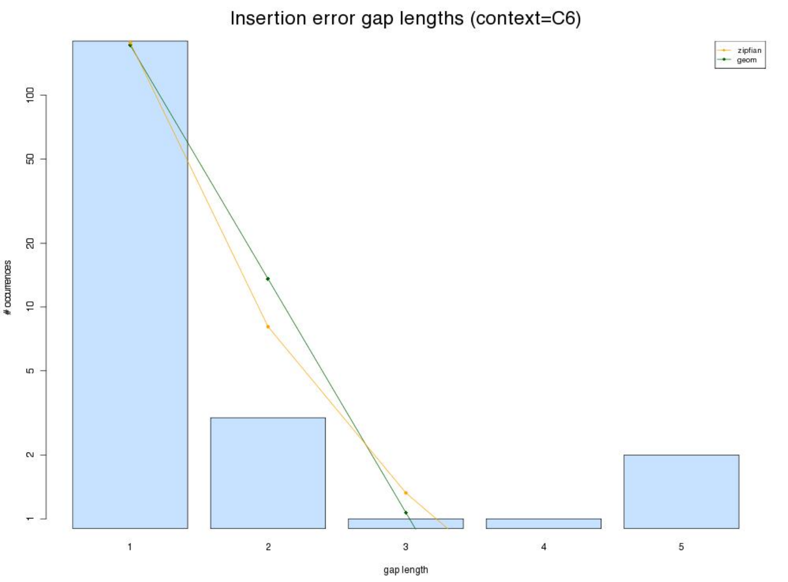 	s = 4.45512825633852<br>X^2 =<br>29.9039486651916<br>p-value =<br>1.44577384723127e-<br>06	beta =<br>0.0788177339901477<br>X^2 =<br>616.235321544838<br>p-value =<br>3.04590638807809e-<br>133
G6	173	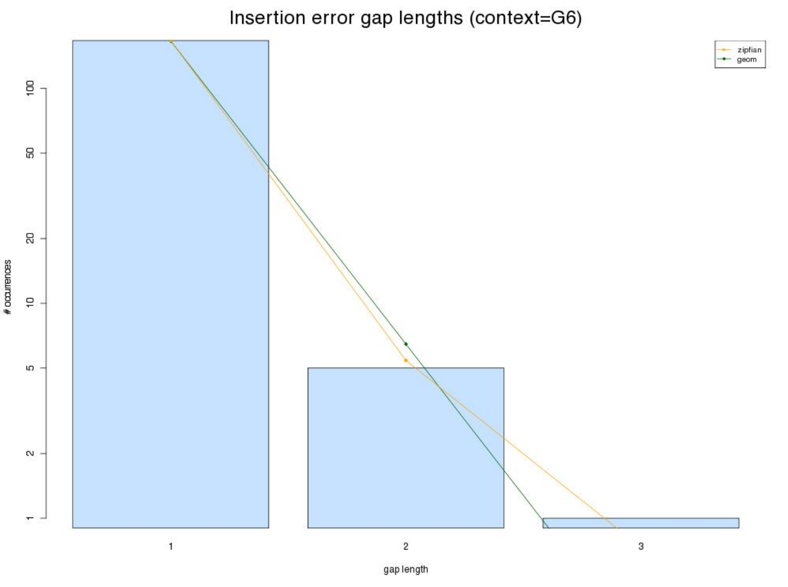 	s = 4.94251324927962<br>X^2 =<br>0.132239959010237<br>p-value =<br>0.716120784842232	beta =<br>0.0388888888888889<br>X^2 =<br>2.56384023901651<br>p-value =<br>0.109332711894606

T6	7491	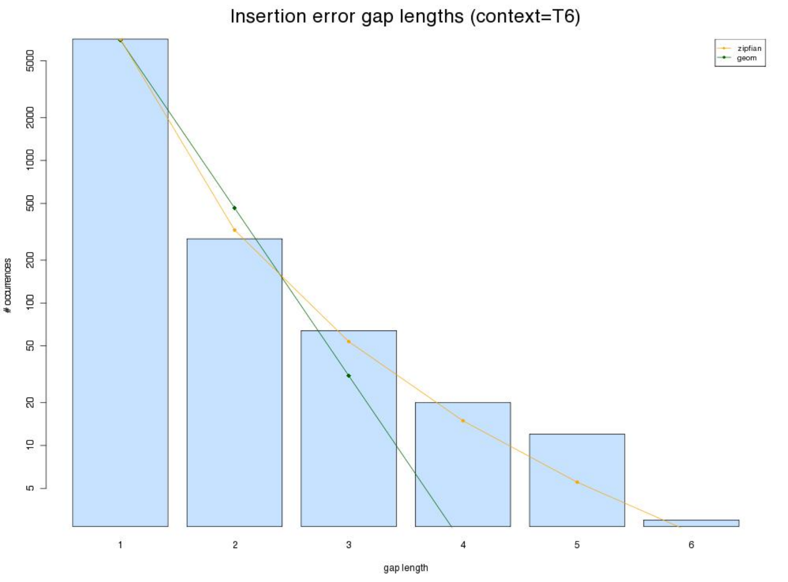	s = 4.44661966316207<br>X^2 =<br>17.2388587721820<br>p-value =<br>0.00173691550252098	beta =<br>0.0664257228315055<br>X^2 =<br>2289.34317502254<br>p-value = 0
A7	3992	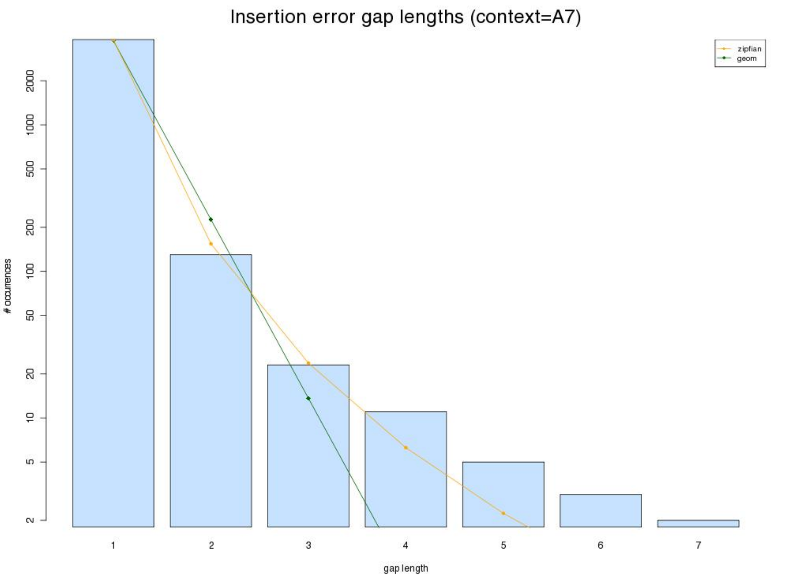	s = 4.6230496813618<br>X^2 =<br>20.2102571511940<br>p-value =<br>0.00114112090348972	beta =<br>0.0602636534839925<br>X^2 =<br>25938.605550418<br>p-value = 0
C7	29		s = 3.34180521073645<br>X^2 =<br>0.298006762708835<br>p-value =<br>0.585134708143864	beta =<br>0.121212121212121<br>X^2 =<br>1.43951042553098<br>p-value =<br>0.230218580655573

		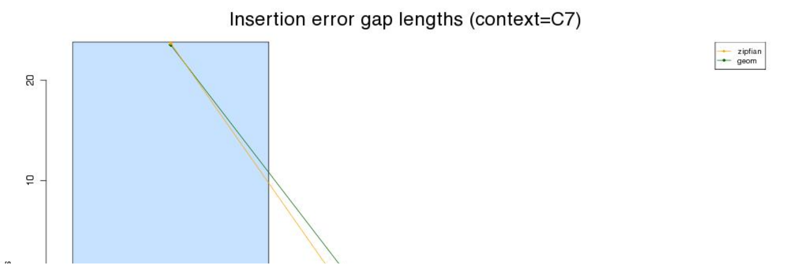		
G7	49	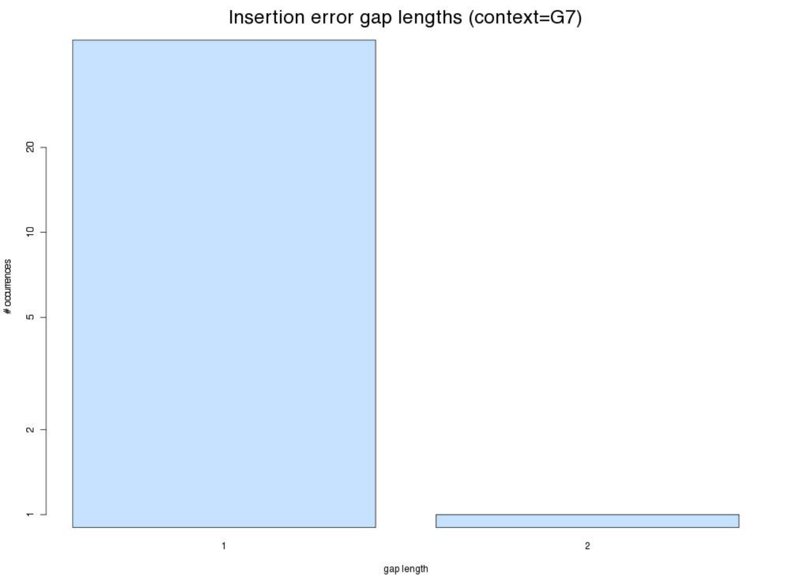	INVALID/ERROR	INVALID/ERROR
T7	4207	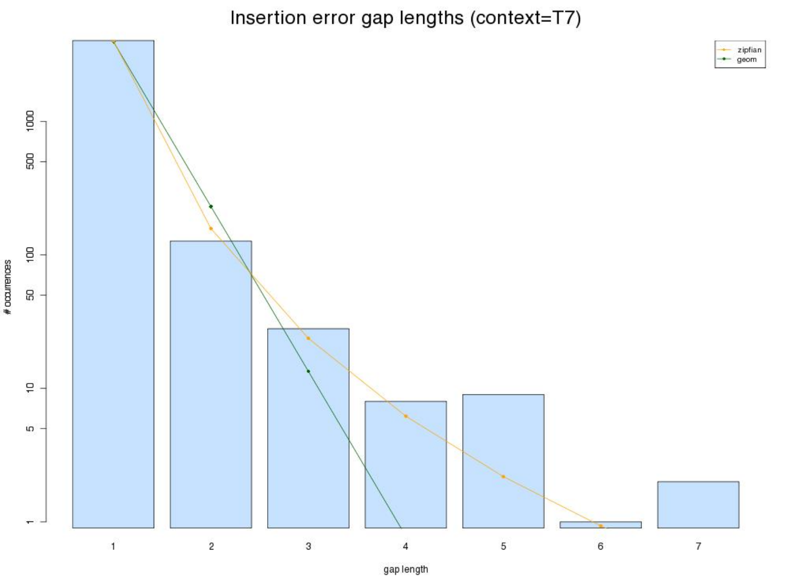	s = 4.67034879362842 X^2 = 33.8752150944833 p-value = 2.52114795902883e-06	beta = 0.0582046115961496 X^2 = 28230.3327213344 p-value = 0
A8	1915		s = 4.44753422931875 X^2 = 32.4318849806104 p-value =	beta = 0.068579766536965 X^2 = 9308.99831740176

		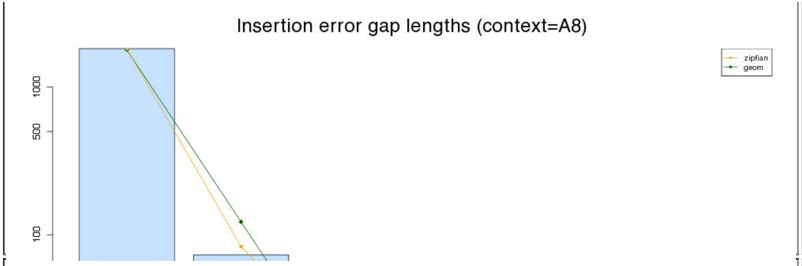	1.56112062253713e-06	p-value = 0
C8	8	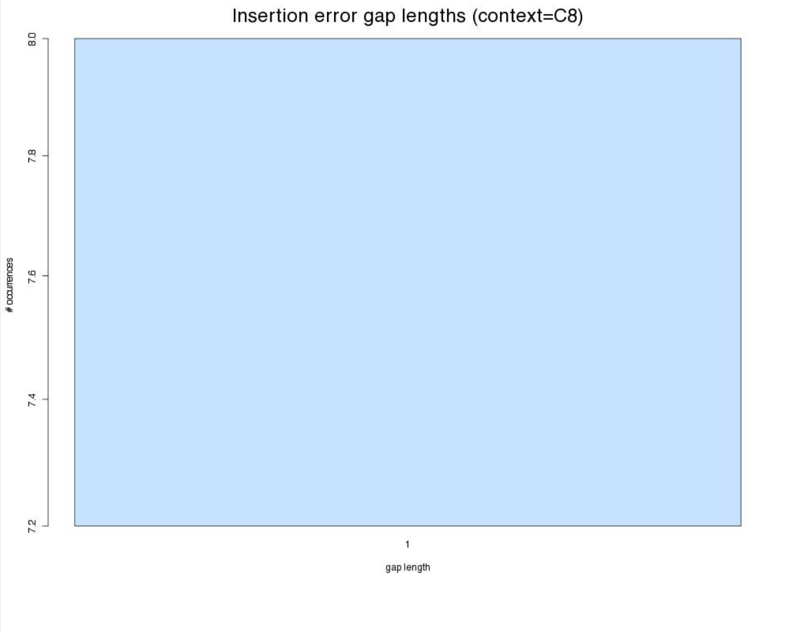	INVALID/ERROR	INVALID/ERROR
G8	4	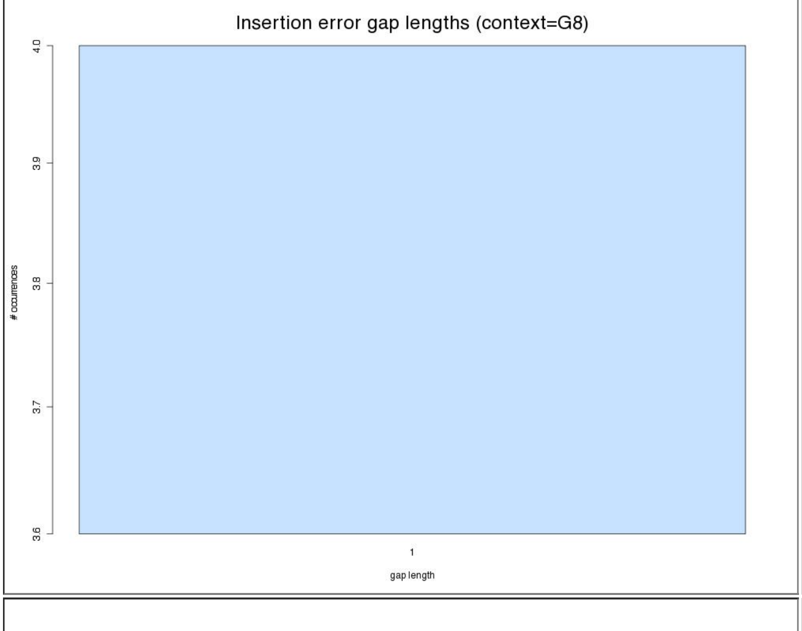	INVALID/ERROR	INVALID/ERROR
T8	1963		s = 4.92716876436796 X^2 = 21.6197380242169 p-value = 7.82615666992692e-05	beta = 0.0466245750364255 X^2 = 1904.36463633256 p-value = 0

		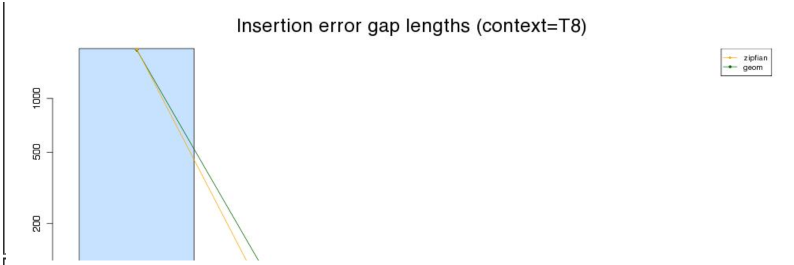		
A9	455	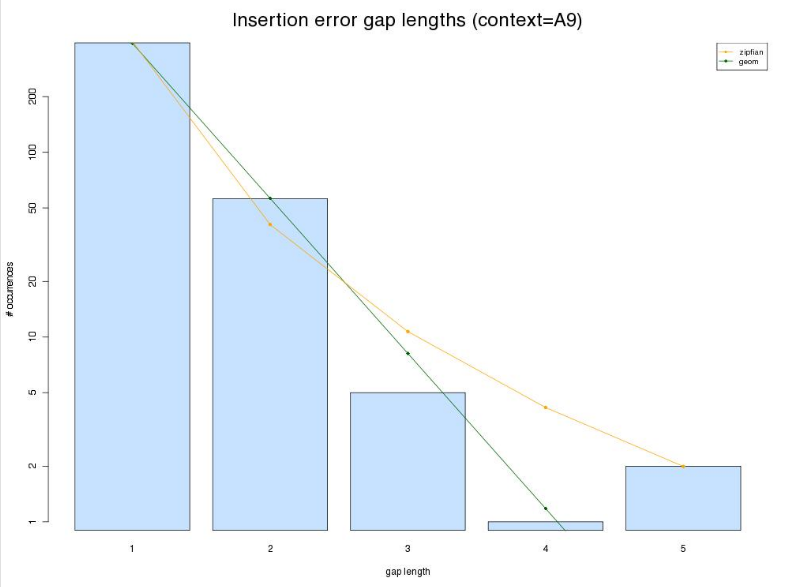	s = 3.28965023224829 X^2 = 11.3449805940114 p-value = 0.00999947376720258	beta = 0.144736842105263 X^2 = 20.8501623746965 p-value = 0.000113094844877843
T9	511	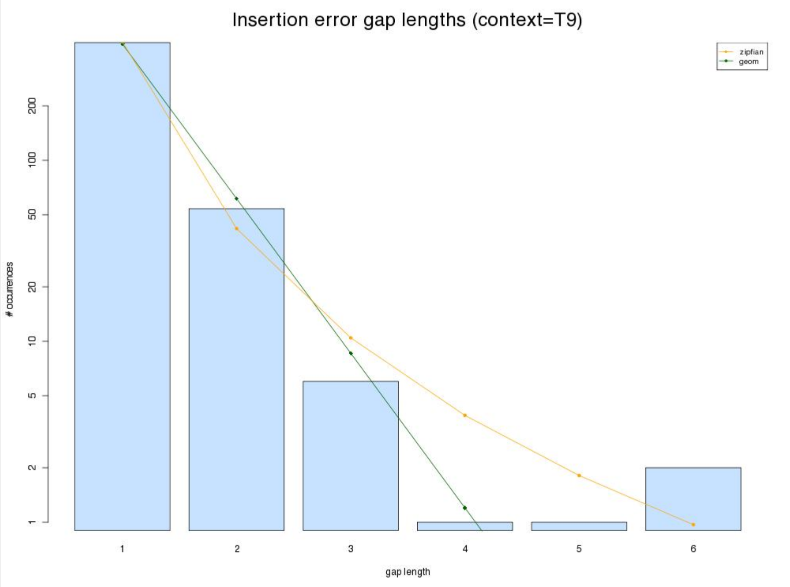	s = 3.42958185392897 X^2 = 9.01779567543008 p-value = 0.0606562096394217	beta = 0.139730639730640 X^2 = 172.814008955156 p-value = 2.60292104286128e-36
			s = 2.60414587794029	beta = 0.179245283018868

A10	87	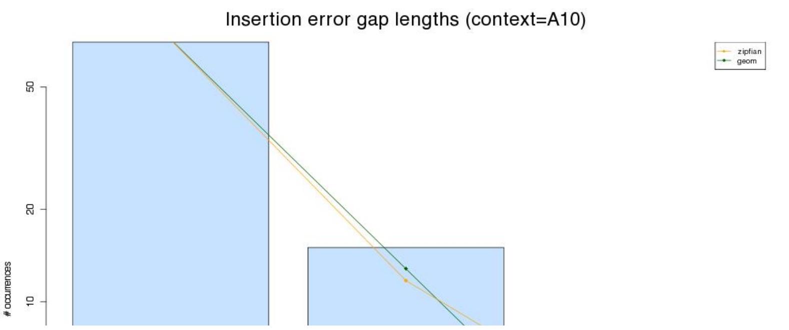	X^2 = 1.99983304510768 p-value = 0.157316534191975	X^2 = 0.443845019349308 p-value = 0.50527244279888
T10	63	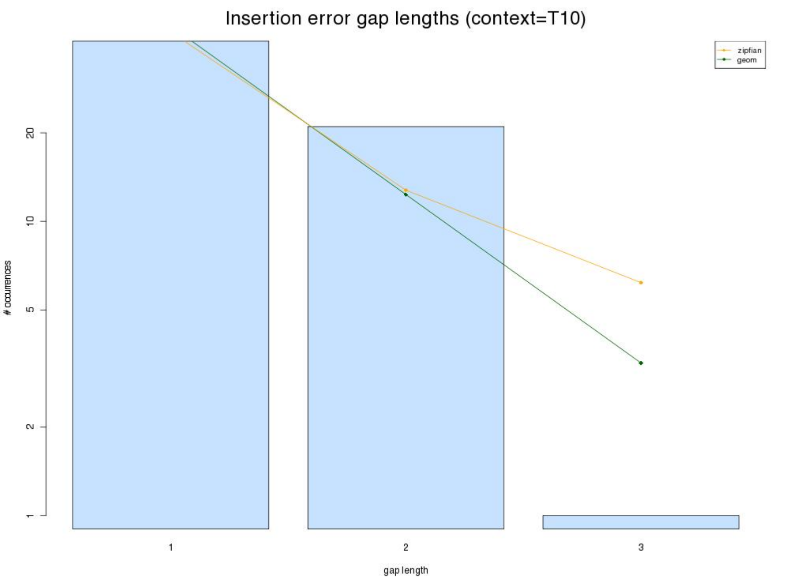	s = 1.78575676709675 X^2 = 9.8635245654481 p-value = 0.00168587821988241	beta = 0.267441860465116 X^2 = 8.25107870865707 p-value = 0.00407277911147701
A11	15	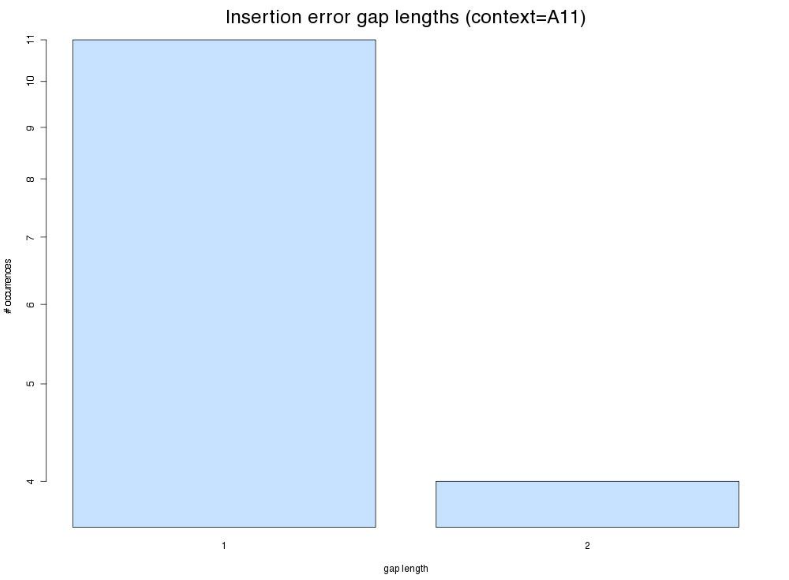	INVALID/ERROR	INVALID/ERROR
T11	15		s = 1.55940140162786 X^2 = 0.0653636910769776	beta = 0.318181818181818 X^2 = 0.923522686810764

		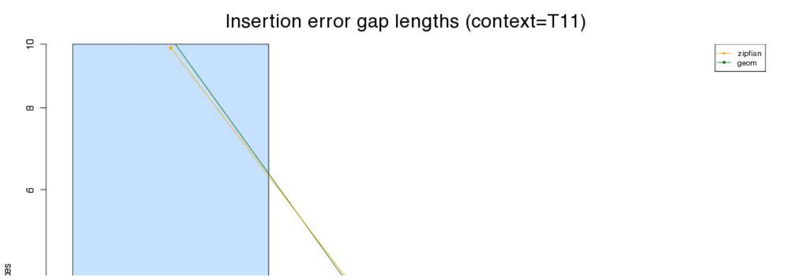	p-value = 0.798210884026135	p-value = 0.336551726558229
A12	3	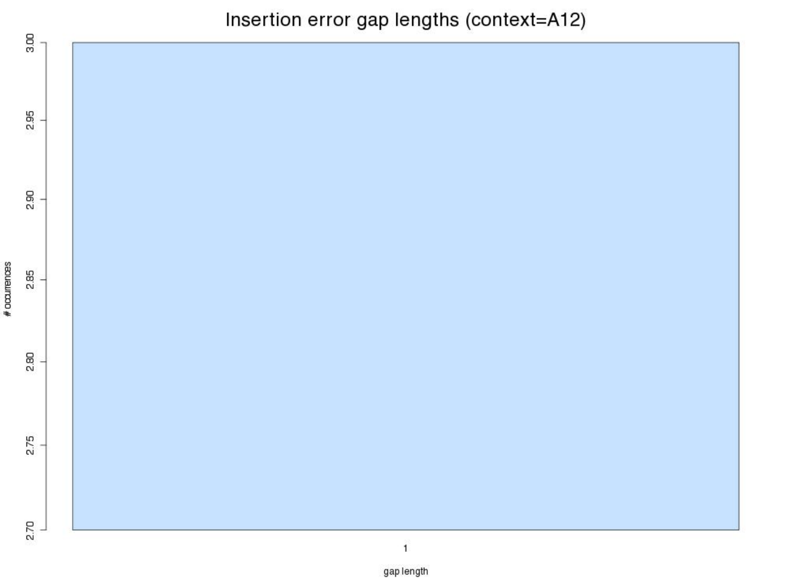	INVALID/ERROR	INVALID/ERROR
T12	2	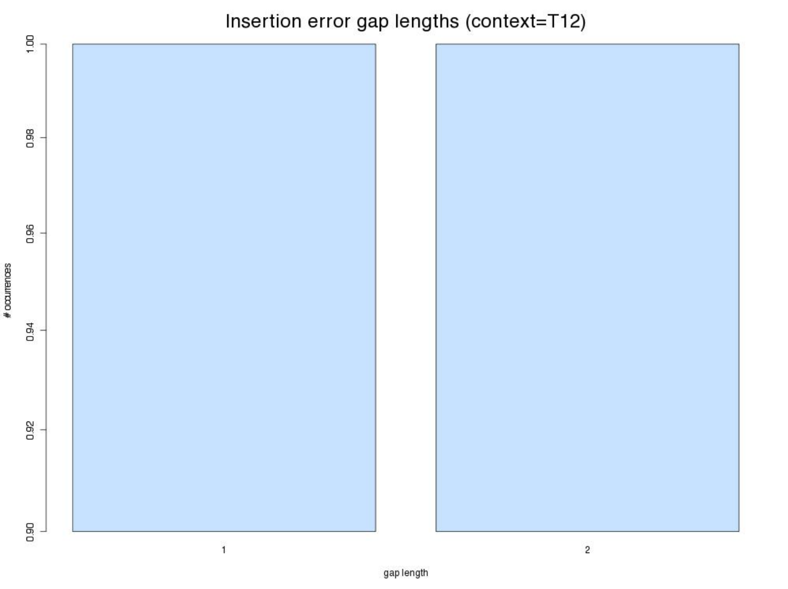	INVALID/ERROR	INVALID/ERROR
A13	3		INVALID/ERROR	INVALID/ERROR

		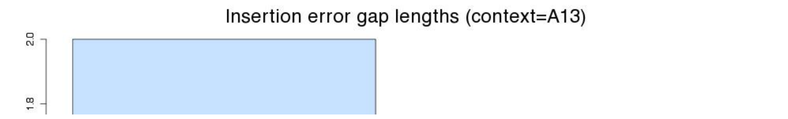		
T13	6	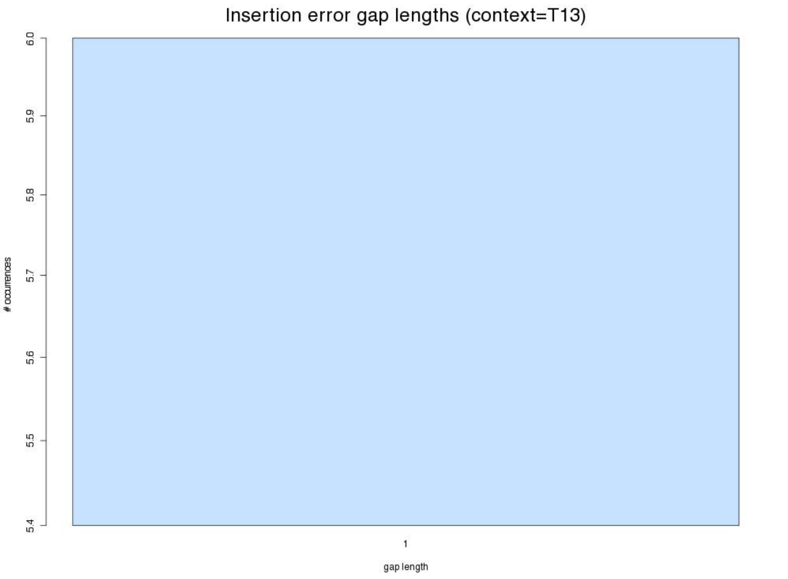	INVALID/ERROR	INVALID/ERROR
A14	4	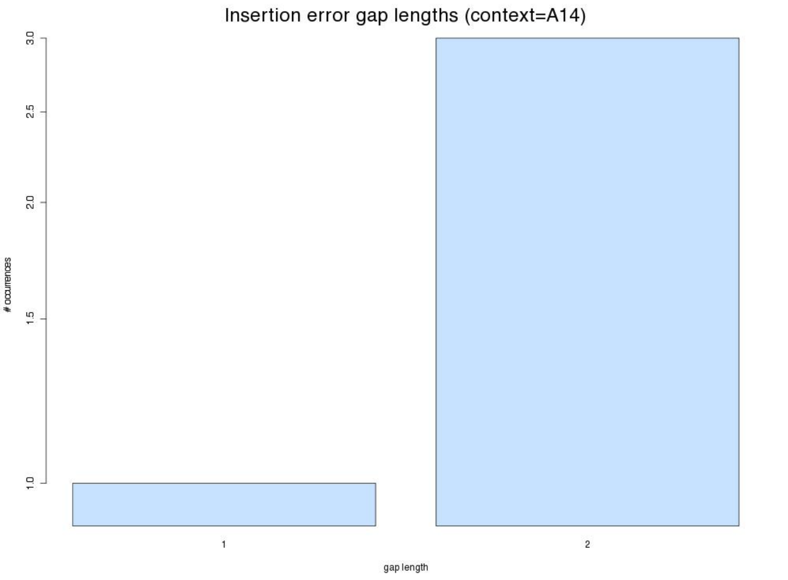	INVALID/ERROR	INVALID/ERROR

T14	13	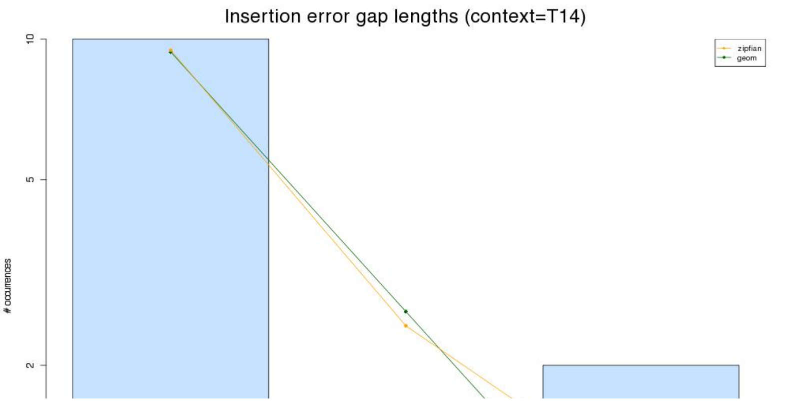	s = 1.96211565844555 X^2 = 1.61472193705244 p-value = 0.203829311490968	beta = 0.277777777777778 X^2 = 3.27710452837233 p-value = 0.0702527686666703
A15	1	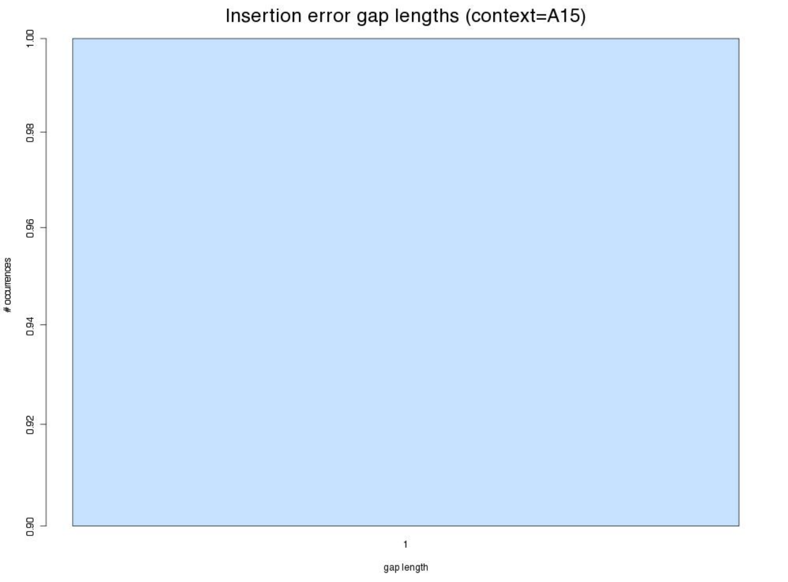	INVALID/ERROR	INVALID/ERROR
T15	7	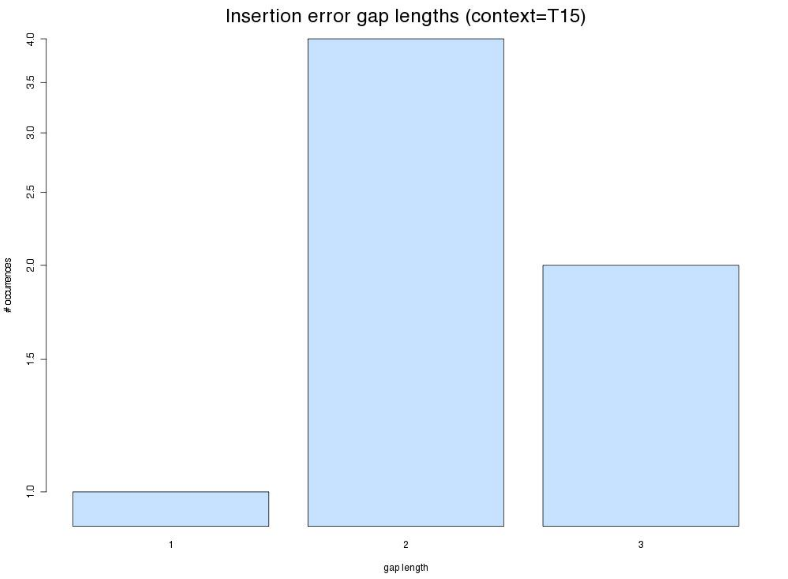	INVALID/ERROR	INVALID/ERROR

A16	4	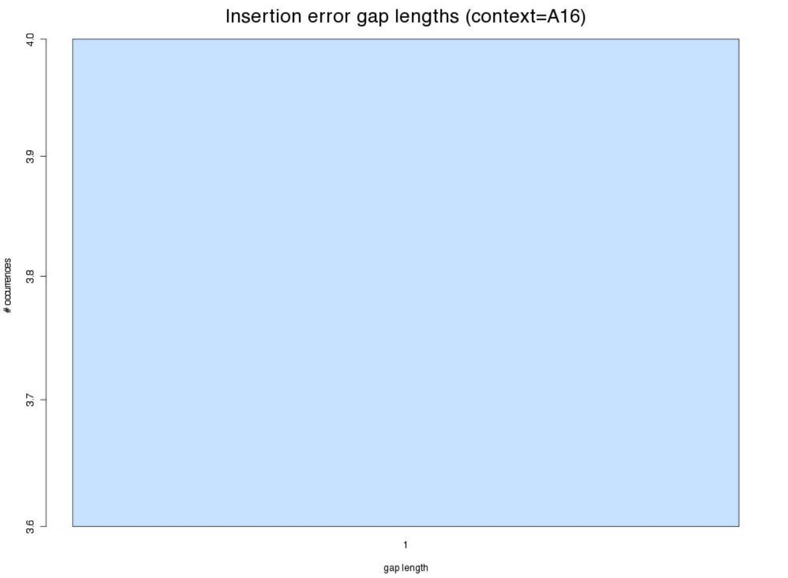	INVALID/ERROR	INVALID/ERROR
A17	1	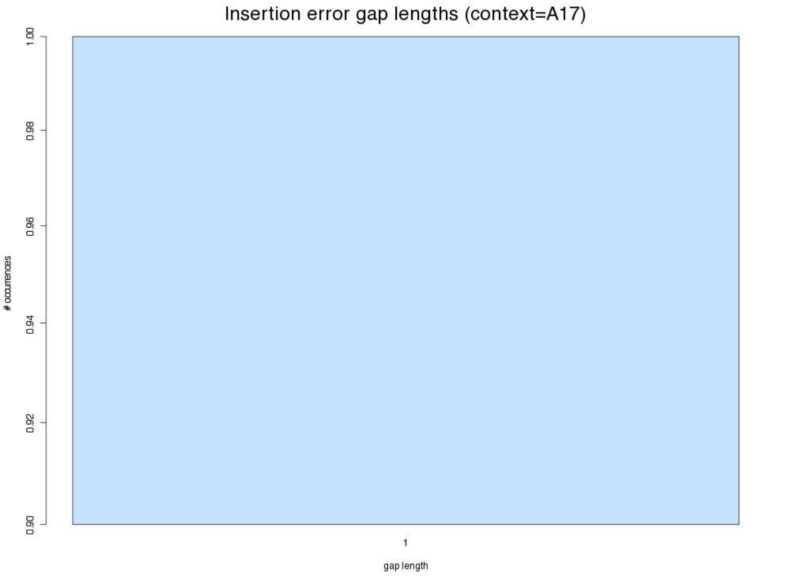	INVALID/ERROR	INVALID/ERROR
T17	2	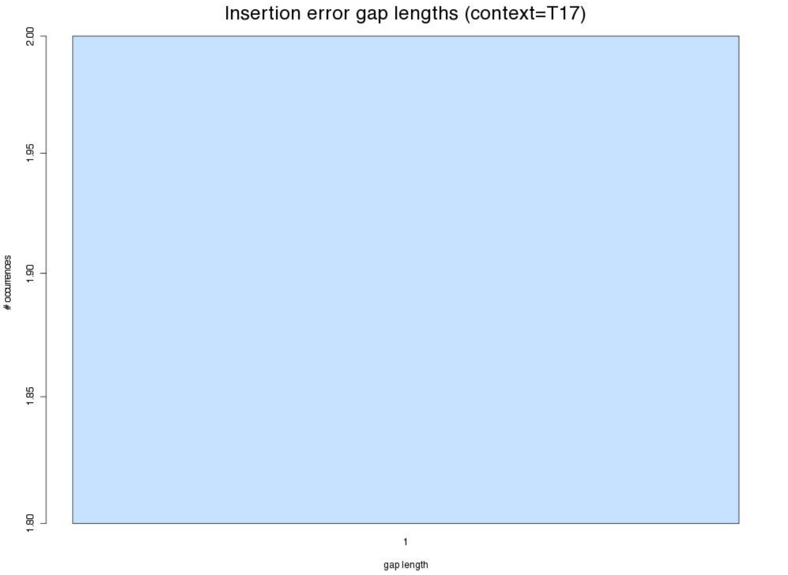	INVALID/ERROR	INVALID/ERROR

A18	5	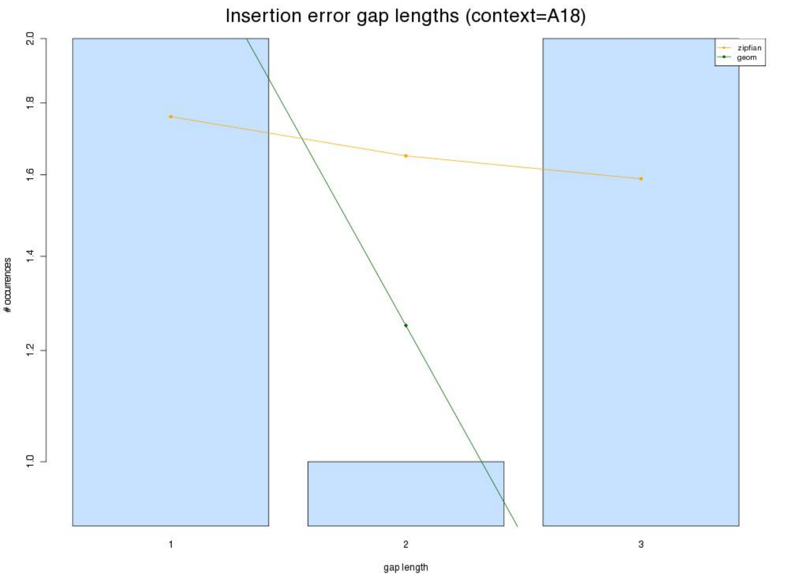	s = 0.0926542887493549 X^2 = 0.395050902693468 p-value = 0.52965630969129	beta = 0.5 X^2 = 3.175 p-value = 0.0747732118999497
T18	1	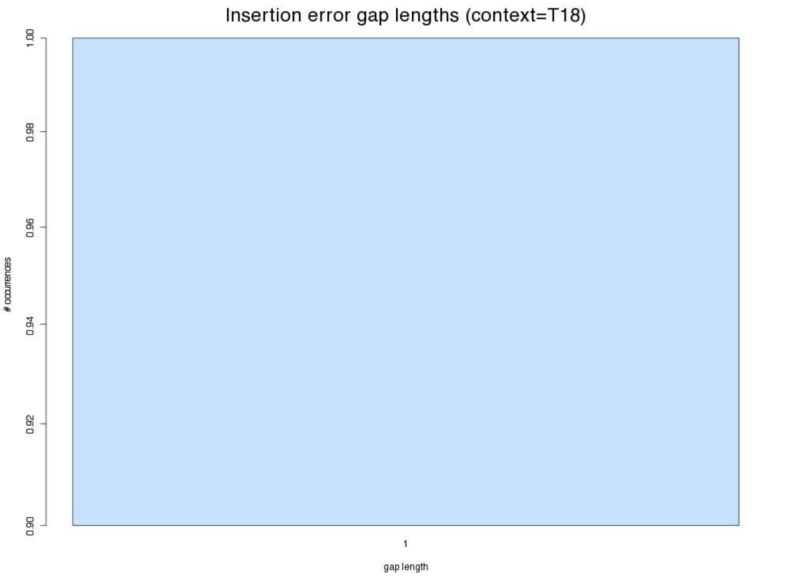	INVALID/ERROR	INVALID/ERROR

Chi square statistics

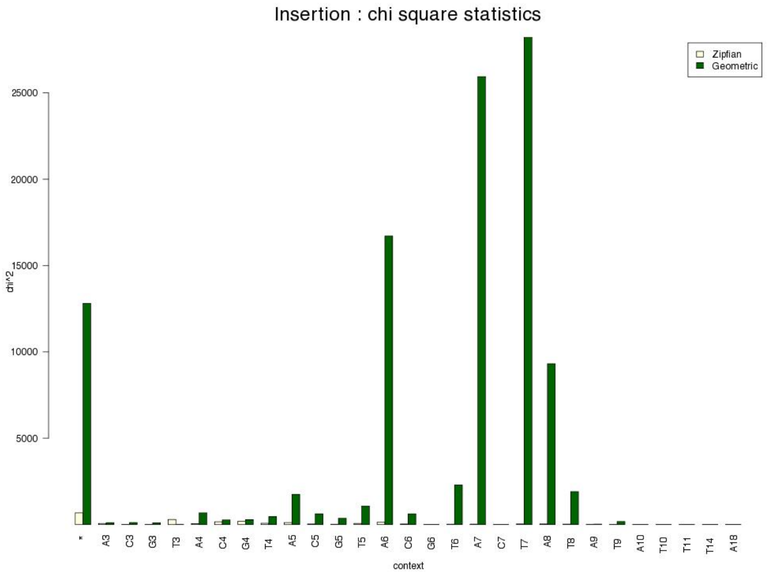

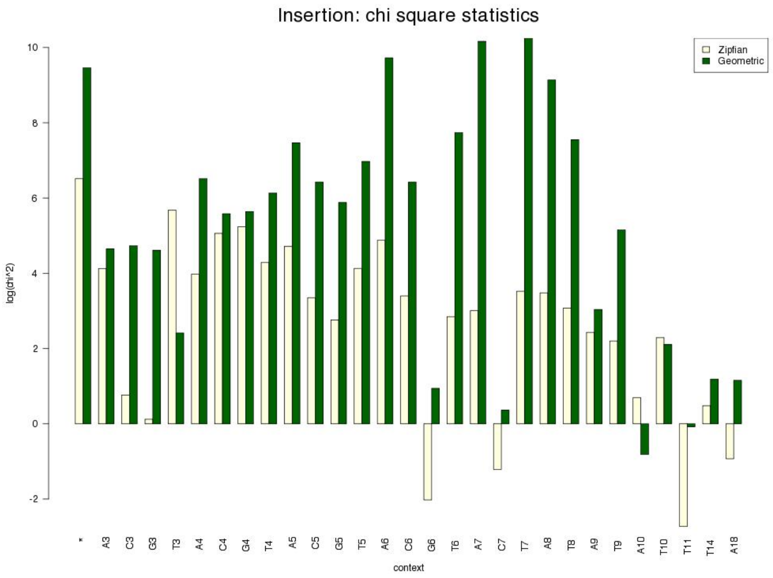

Average Pearson's residuals

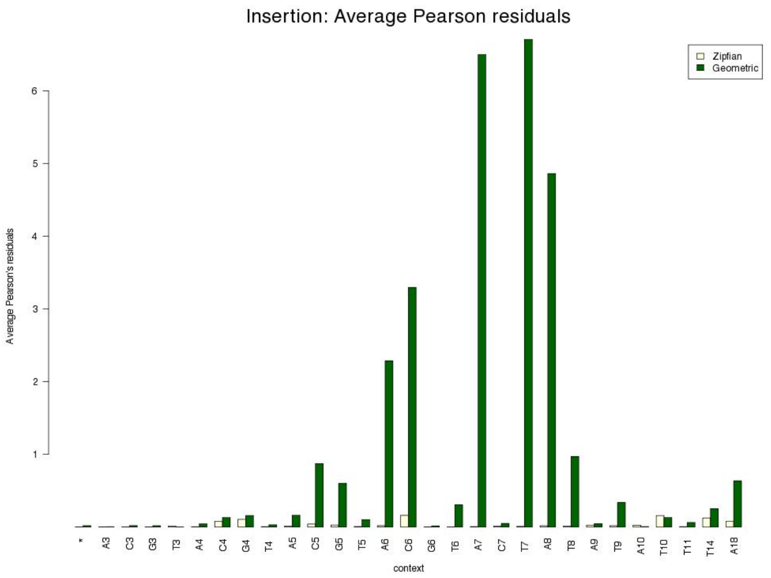
Insertion: Average Pearson residuals
Zipfian
Geometric
Average Pearson's residuals
context

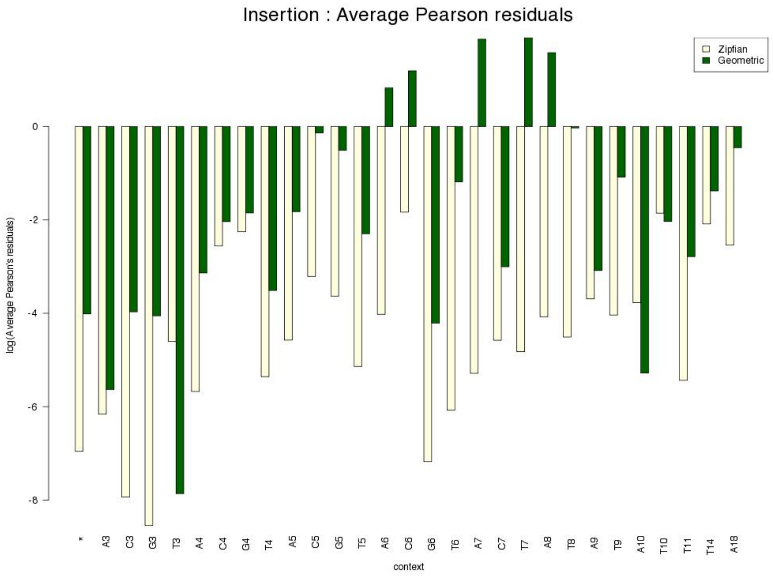
Insertion : Average Pearson residuals
Zipfian
Geometric
log(Average Pearson's residuals)
context

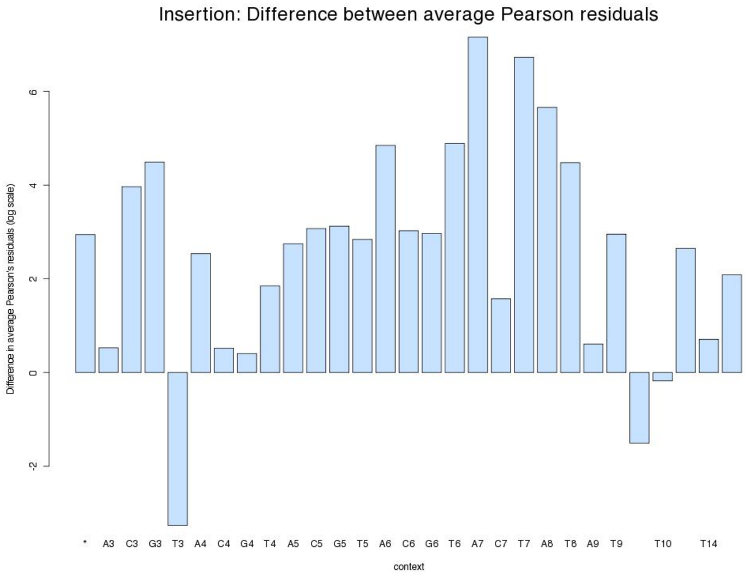
Insertion: Difference between average Pearson residuals
Difference in average Pearson's residuals (log scale)
context

Mann U Tests

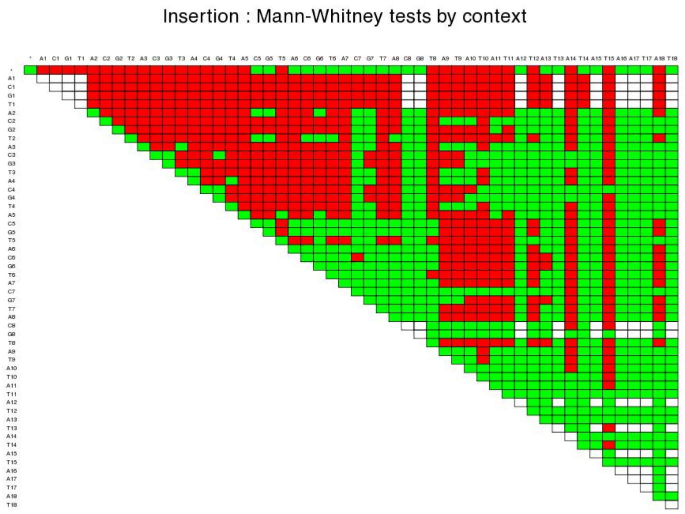

Mann U Tests by context length

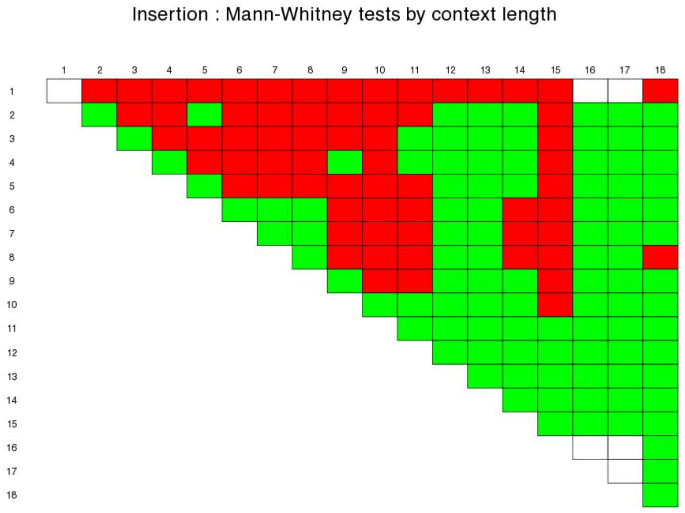

celera Deletion

Deletion per context

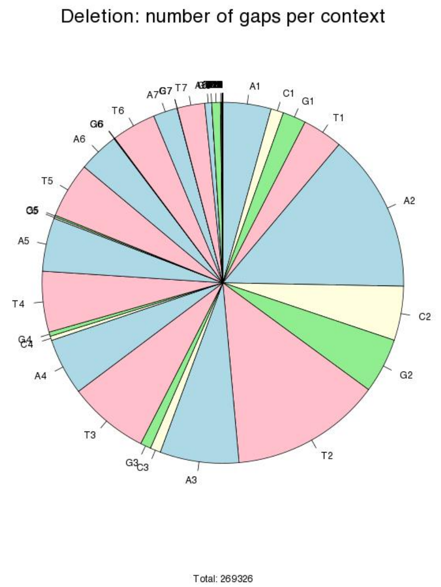

Deletion per context

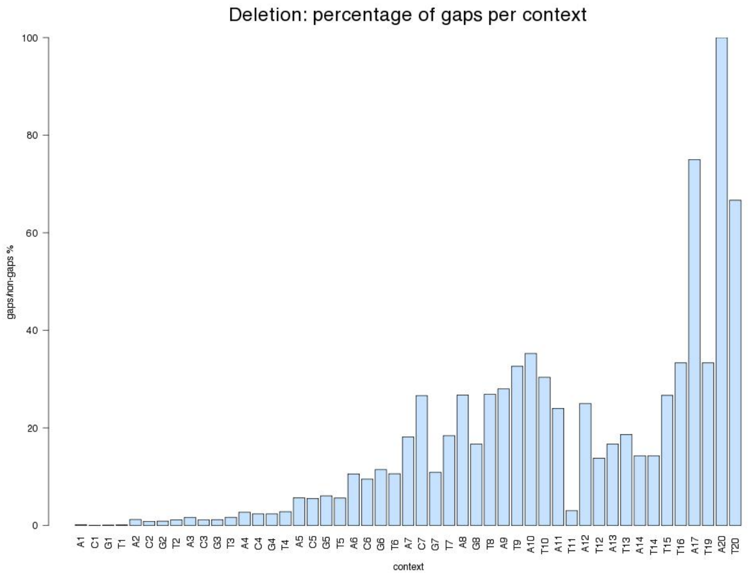

Context	#Data	Gap lengths	Zipfian fit	Geom fit
*	269326	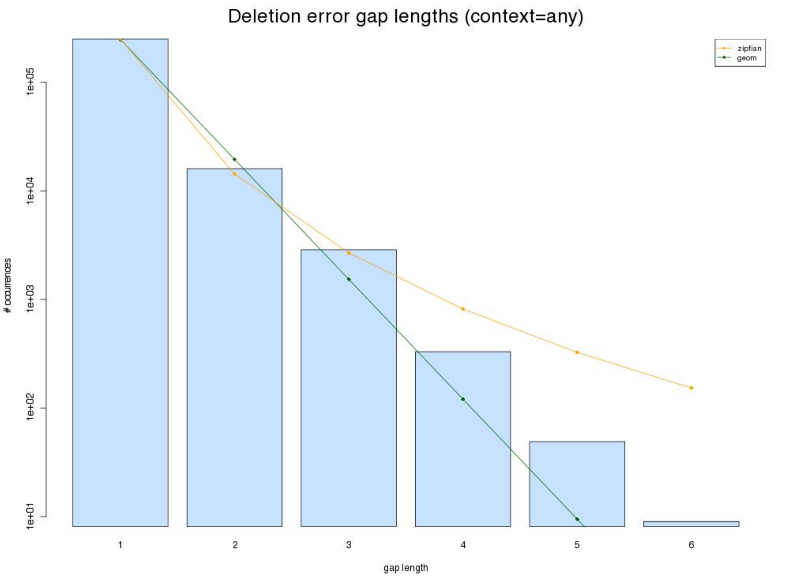	s = 4.13157135099768<br>X^2 =<br>876.48171340316<br>p-value =<br>2.07547219665432e-<br>188	beta =<br>0.0786225488183696<br>X^2 =<br>2425.60061768410<br>p-value = 0

A1	11606	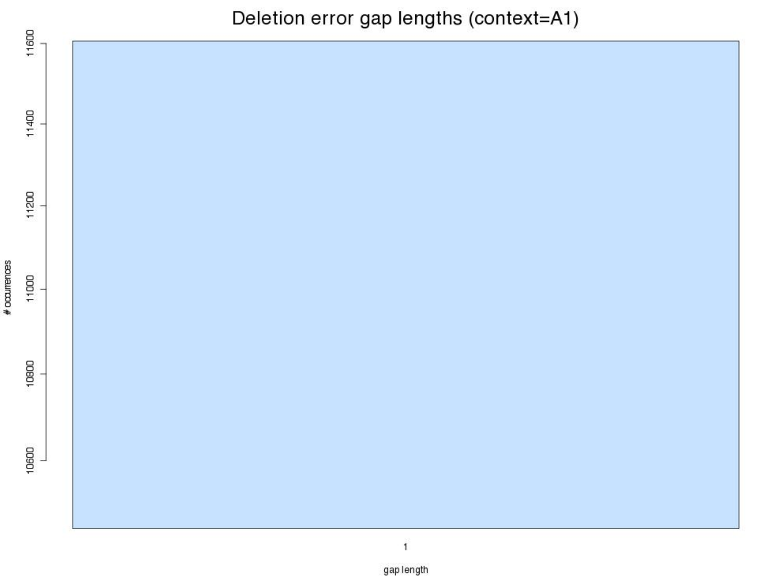	INVALID/ERROR	INVALID/ERROR
C1	3041	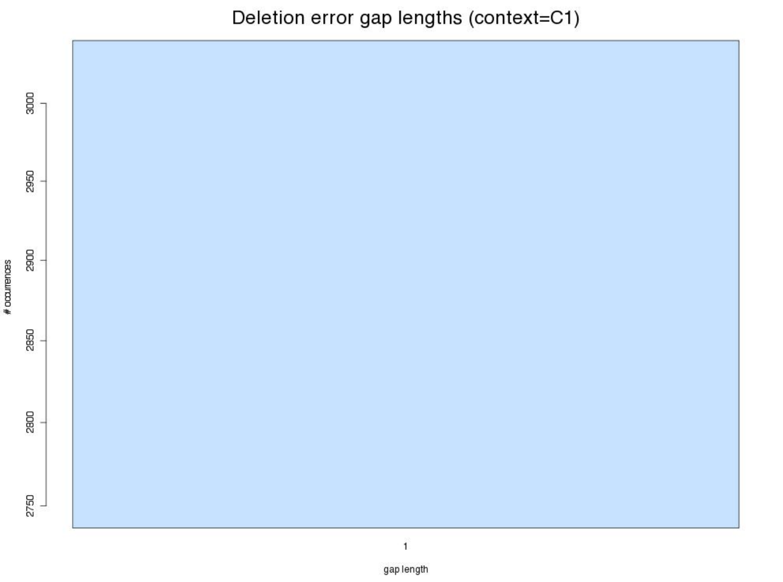	INVALID/ERROR	INVALID/ERROR
G1	5604		INVALID/ERROR	INVALID/ERROR

		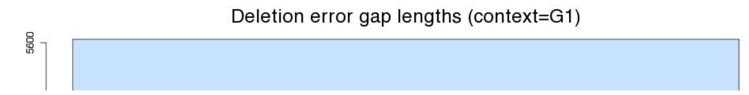		
T1	9675	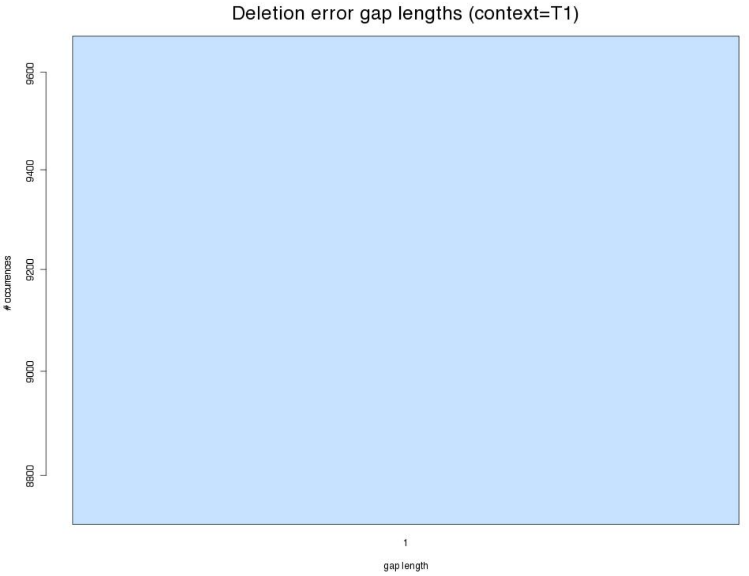	INVALID/ERROR	INVALID/ERROR
A2	38216	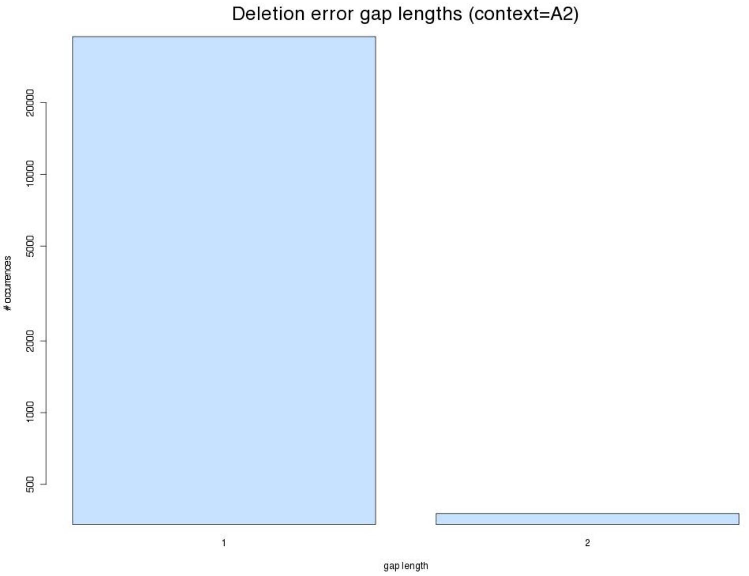	INVALID/ERROR	INVALID/ERROR
C2	13068		INVALID/ERROR	INVALID/ERROR

		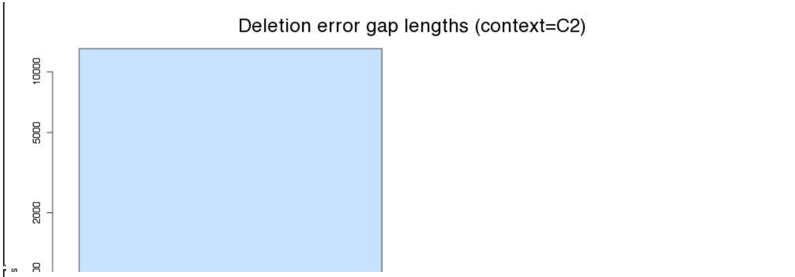		
G2	13276	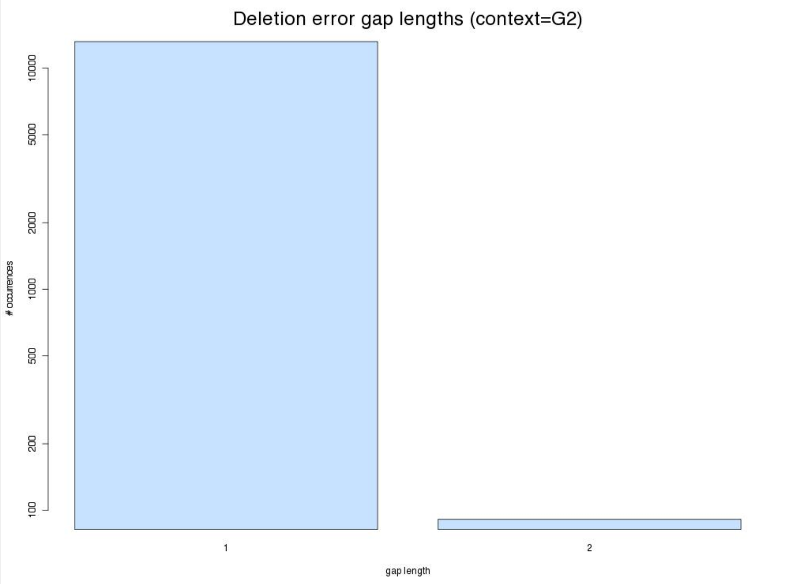	INVALID/ERROR	INVALID/ERROR
T2	36333	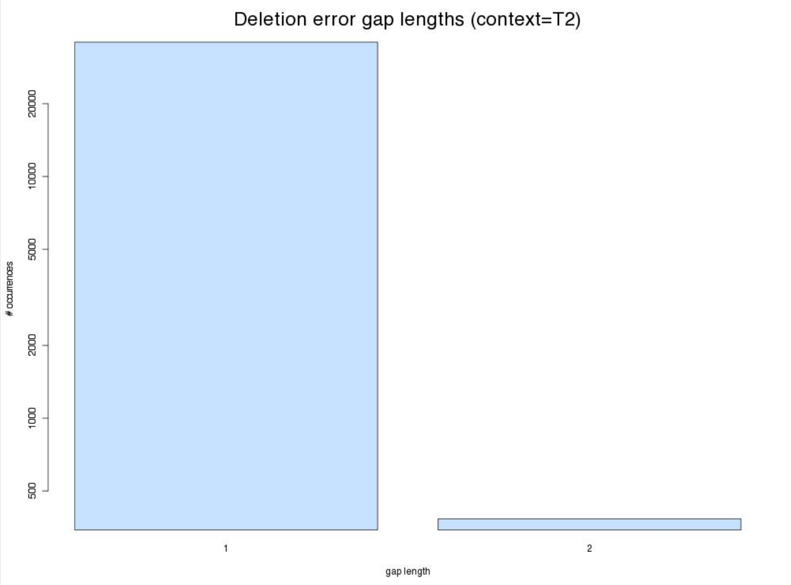	INVALID/ERROR	INVALID/ERROR
A3	19009		s = 2.24413574843526<br>X^2 =<br>2323.32907621064<br>p-value = 0	beta =<br>0.211342986350247<br>X^2 =<br>1674.19447134317<br>p-value = 0

		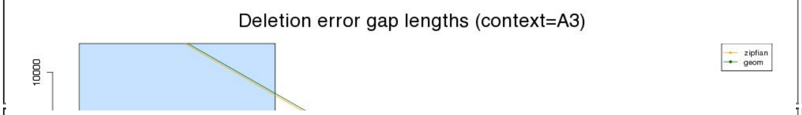		
C3	2519	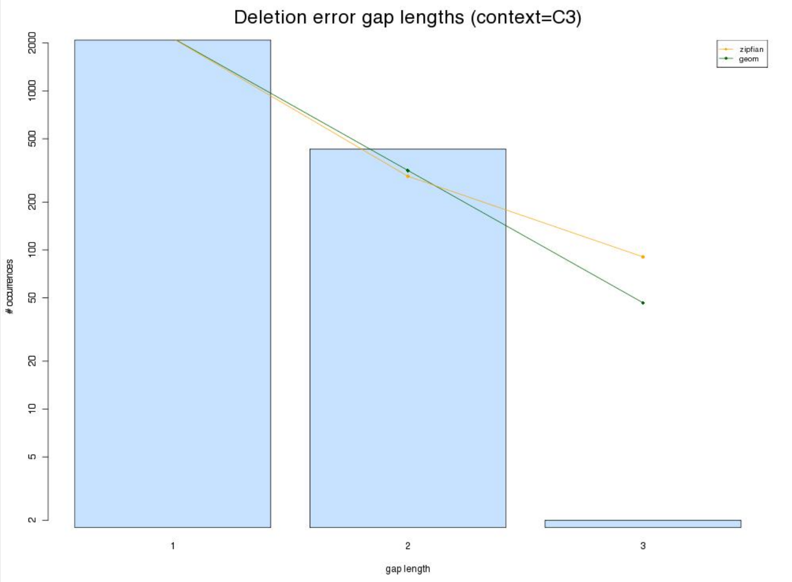	s = 2.87834920639417 X^2 = 155.471383421793 p-value = 1.10454400621024e-35	beta = 0.147257955314827 X^2 = 86.0369680562996 p-value = 1.76603135663117e-20
G3	2545	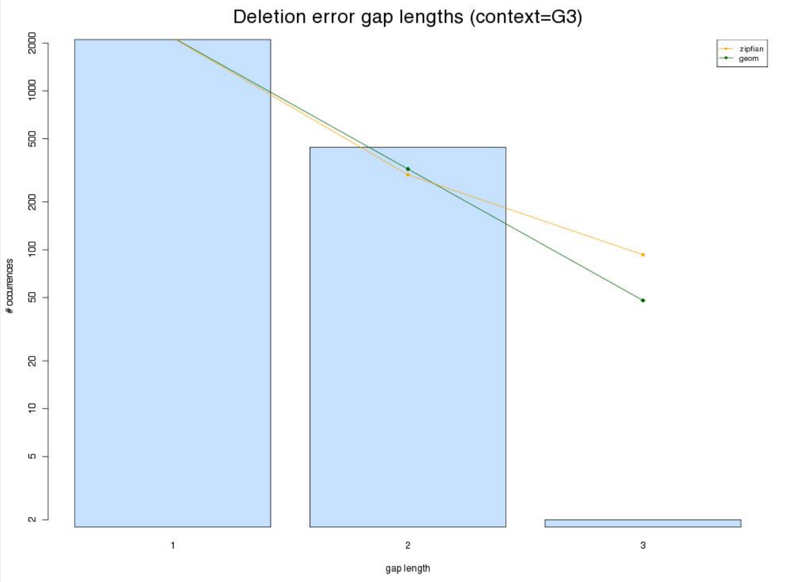	s = 2.86066989641126 X^2 = 160.539419581068 p-value = 8.6256703808739e-37	beta = 0.148829431438127 X^2 = 89.6002642080527 p-value = 2.91485238963088e-21

T3	19445	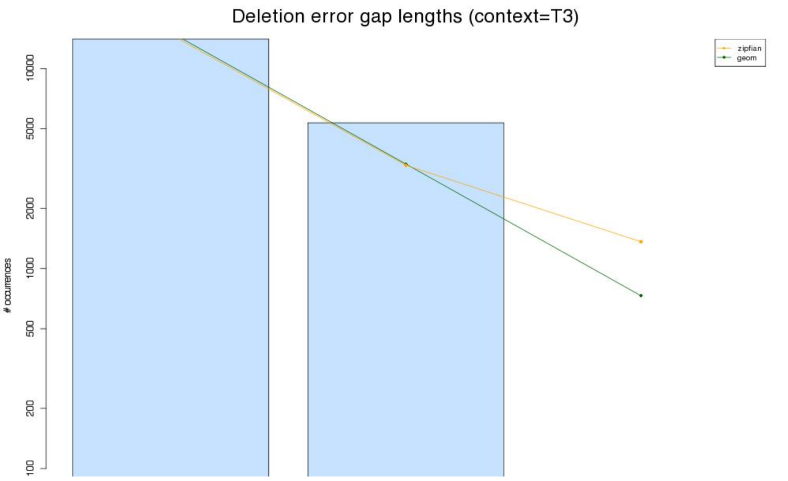	s = 2.1715068127237<br>X^2 =<br>2591.6692420927<br>p-value = 0	beta =<br>0.219452472703918<br>X^2 =<br>1935.16470101859<br>p-value = 0
A4	13733	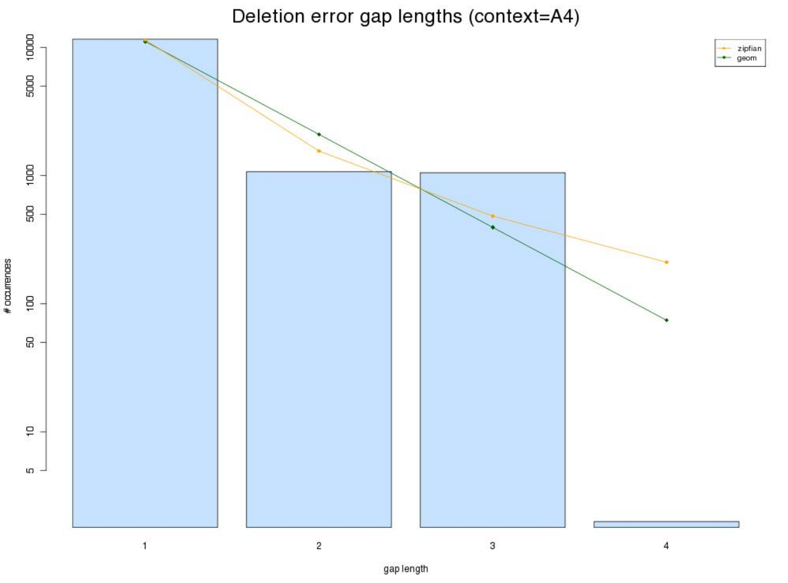	s = 2.88299141271325<br>X^2 =<br>1029.62636915895<br>p-value =<br>2.62708863775782e-<br>224	beta =<br>0.18821304013714<br>X^2 =<br>1687.72528790234<br>p-value = 0
C4	1045	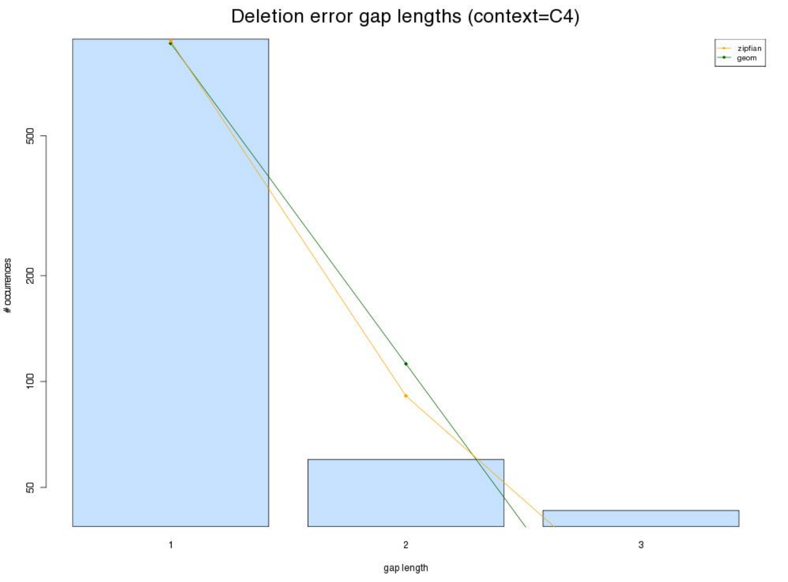	s = 3.35282259148414<br>X^2 =<br>27.1891937145432<br>p-value =<br>1.84486960908434e-07	beta =<br>0.122586062132662<br>X^2 =<br>87.0876823621803<br>p-value =<br>1.03815101999855e-<br>20

G4	1015	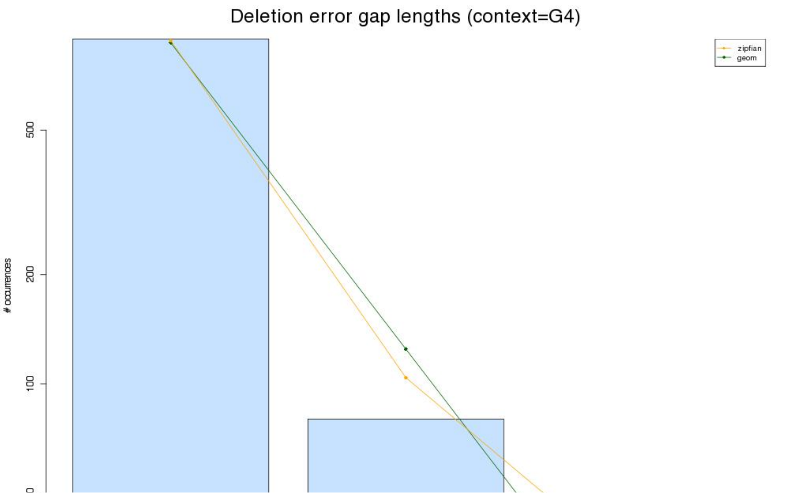	s = 3.08199505097195 X^2 = 13.3740690593095 p-value = 0.000255127233213948	beta = 0.143459915611814 X^2 = 57.59257234837 p-value = 3.22440911336048e-14
T4	14640	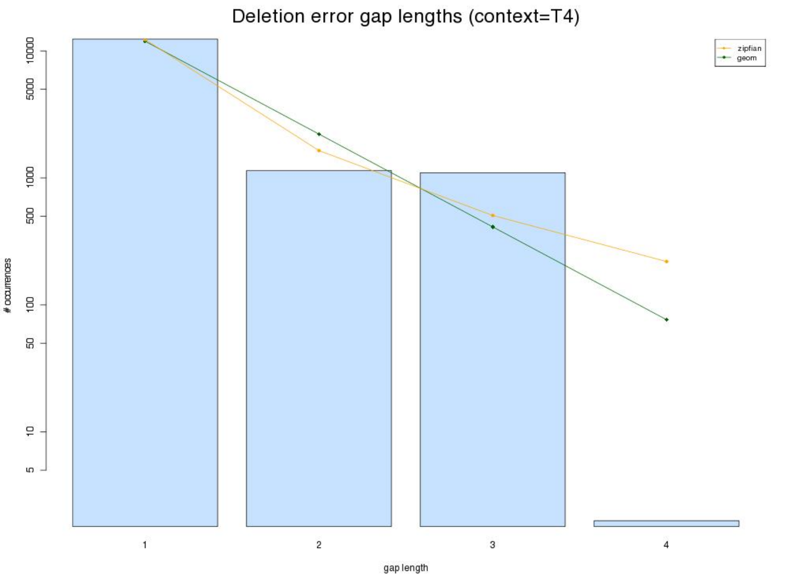	s = 2.90145274416573 X^2 = 1058.15945797622 p-value = 1.67337475802733e-230	beta = 0.185852519185853 X^2 = 1752.54435536755 p-value = 0
A5	12889	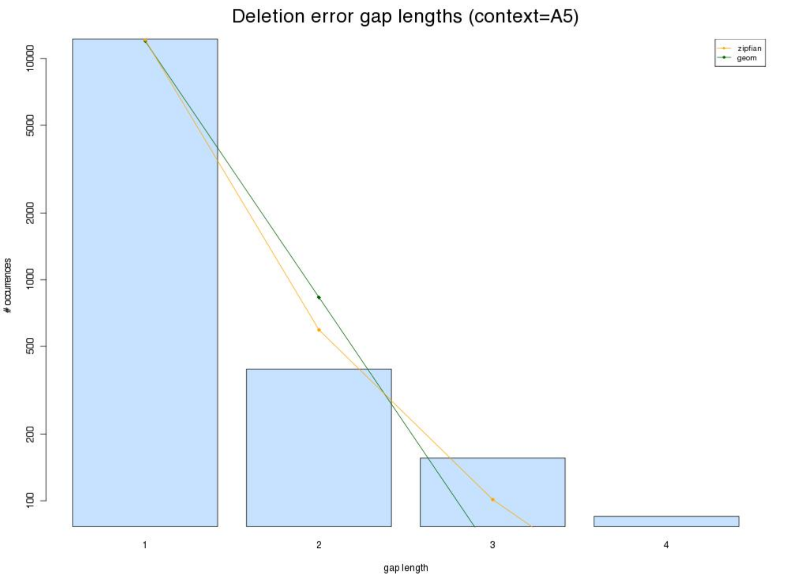	s = 4.35875597991466 X^2 = 205.867336482474 p-value = 1.97913710368347e-45	beta = 0.0693862815884476 X^2 = 2040.7030351961 p-value = 0

C5	398	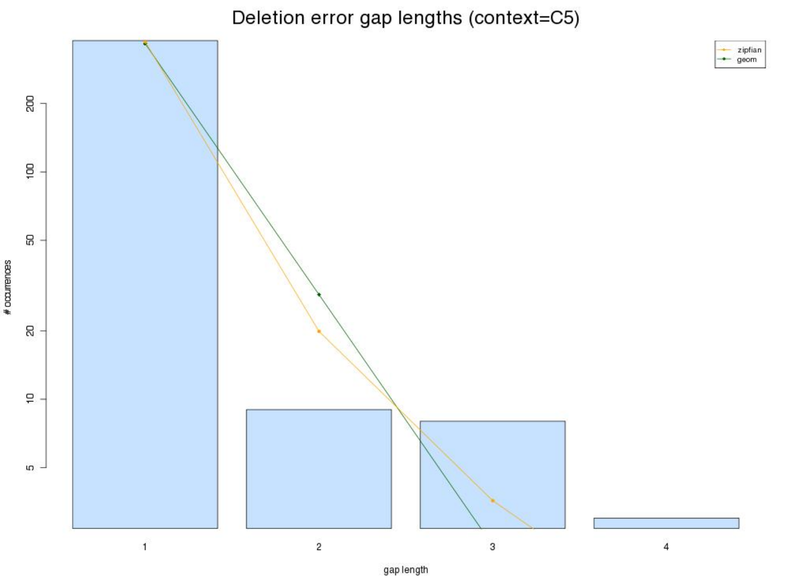	s = 4.23086502491977<br>X^2 =<br>15.0385702155633<br>p-value =<br>0.000542520270765359	beta =<br>0.0787037037037037<br>X^2 =<br>72.9902256491336<br>p-value =<br>1.41375412093137e-<br>16
G5	401	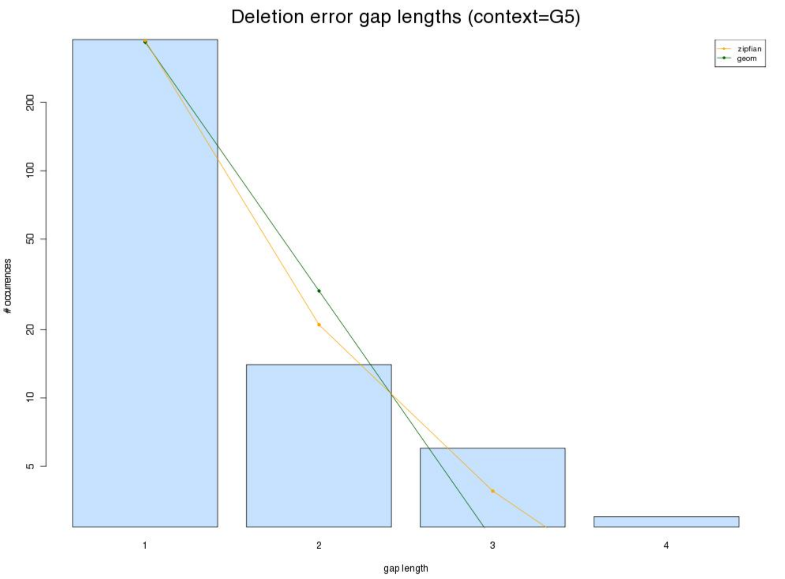	s = 4.15850895201487<br>X^2 =<br>6.33235852506228<br>p-value =<br>0.0421643895996077	beta =<br>0.0802752293577982<br>X^2 =<br>55.3435408481379<br>p-value =<br>9.60069781460717e-<br>13

T5	13408	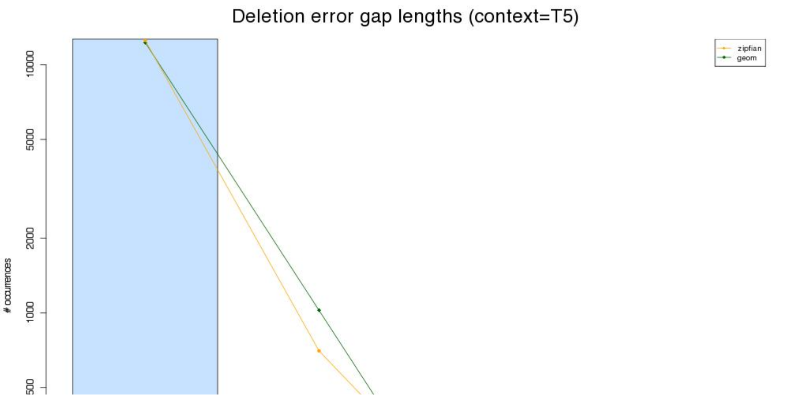	s = 4.15637340105945<br>X^2 =<br>496.502775669279<br>p-value =<br>1.533882662343e-108	beta =<br>0.0833390305599234<br>X^2 =<br>3522.86368954971<br>p-value = 0
A6	9772	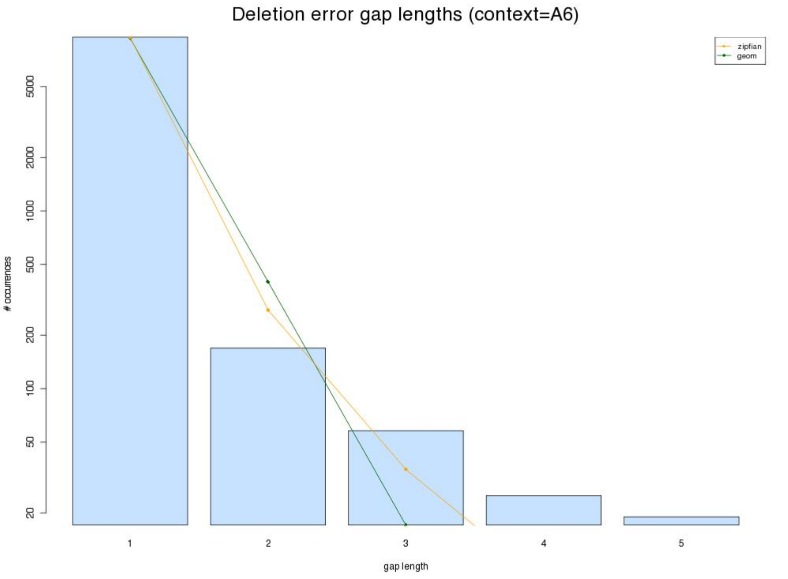	s = 5.0920133118168<br>X^2 =<br>195.401106363212<br>p-value =<br>4.15717406593749e-42	beta =<br>0.0427115987460814<br>X^2 =<br>12599.4554246289<br>p-value = 0
C6	92	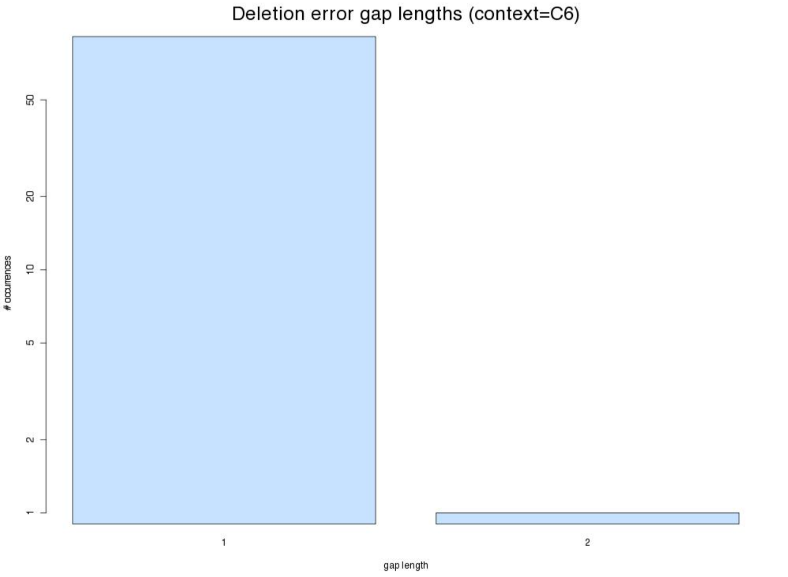	INVALID/ERROR	INVALID/ERROR

G6	119	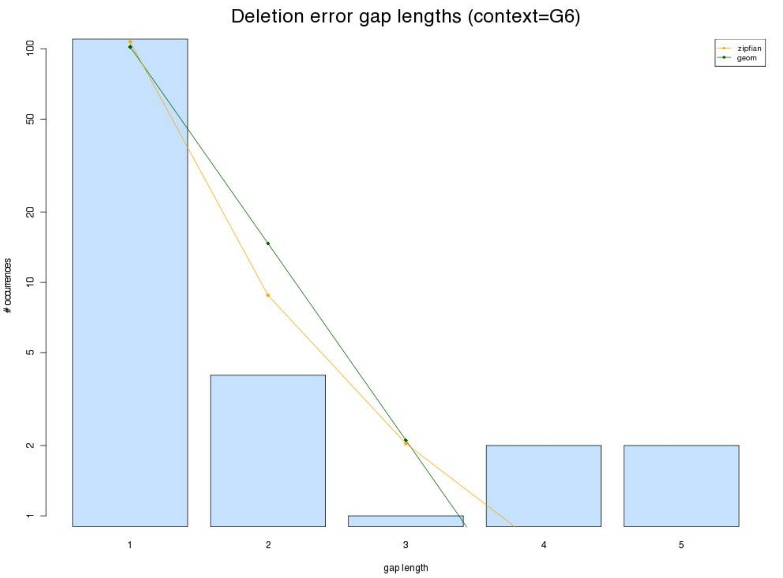	s = 3.60558117814859 X^2 = 14.1760177094006 p-value = 0.00267509414132328	beta = 0.143884892086331 X^2 = 106.113944017656 p-value = 7.52510381474963e-23
T6	10658	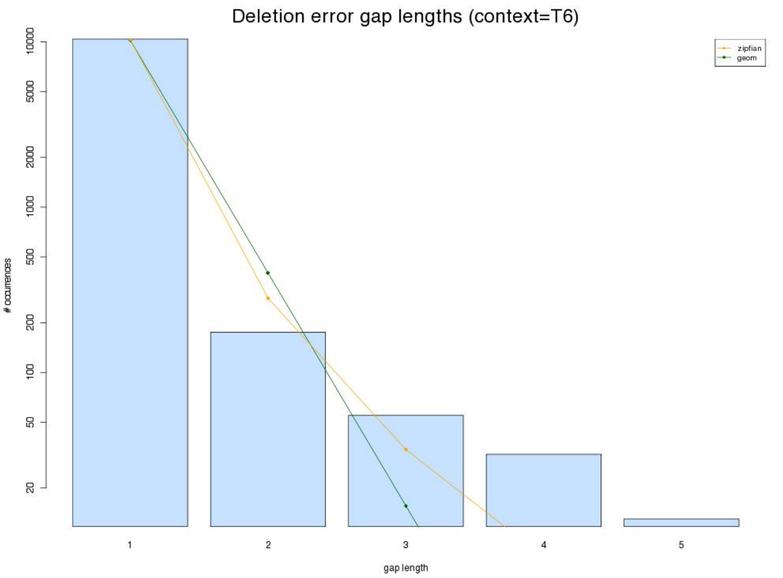	s = 5.19894811245341 X^2 = 177.283716591884 p-value = 3.40434745364146e-38	beta = 0.039040663601118 X^2 = 8921.56044567832 p-value = 0

A7	5824	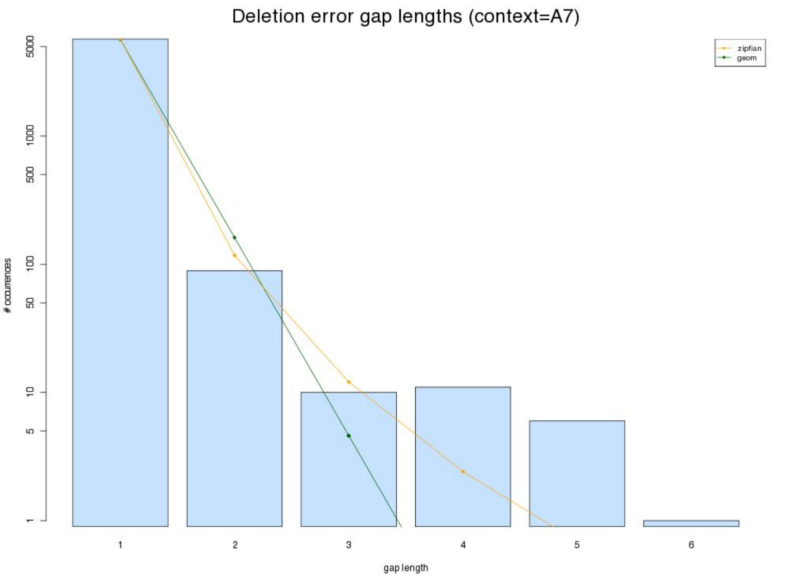	s = 5.6024155163407<br>X^2 =<br>80.838019148863<br>p-value =<br>1.15730248994697e-16	beta =<br>0.0285237698081734<br>X^2 =<br>19897.8293578799<br>p-value = 0
C7	41	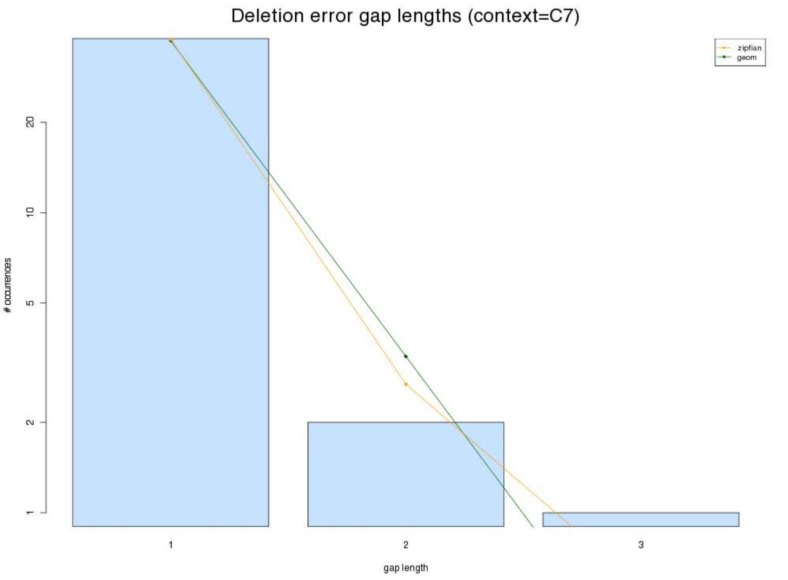	s = 3.8157874138771<br>X^2 =<br>0.497688405074037<br>p-value =<br>0.48051758162324	beta =<br>0.0888888888888889<br>X^2 =<br>2.21945691783175<br>p-value =<br>0.136280990779991
G7	18	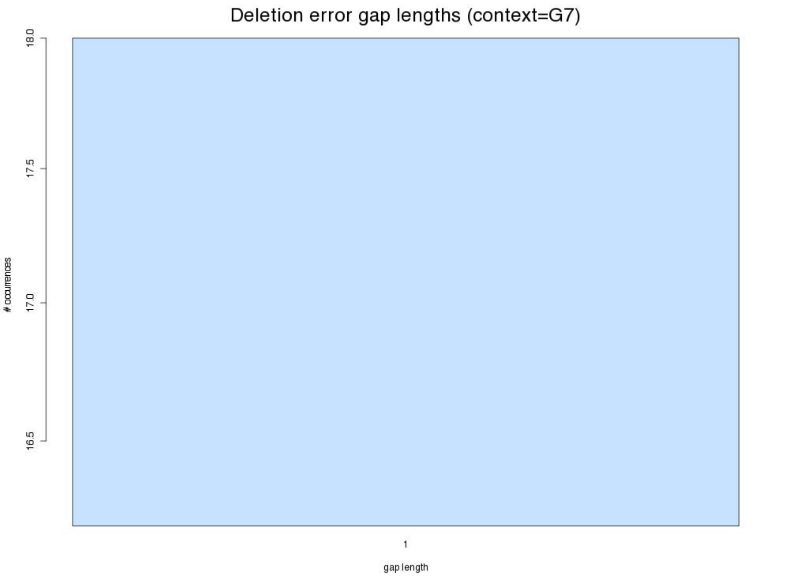	INVALID/ERROR	INVALID/ERROR

T7	6622	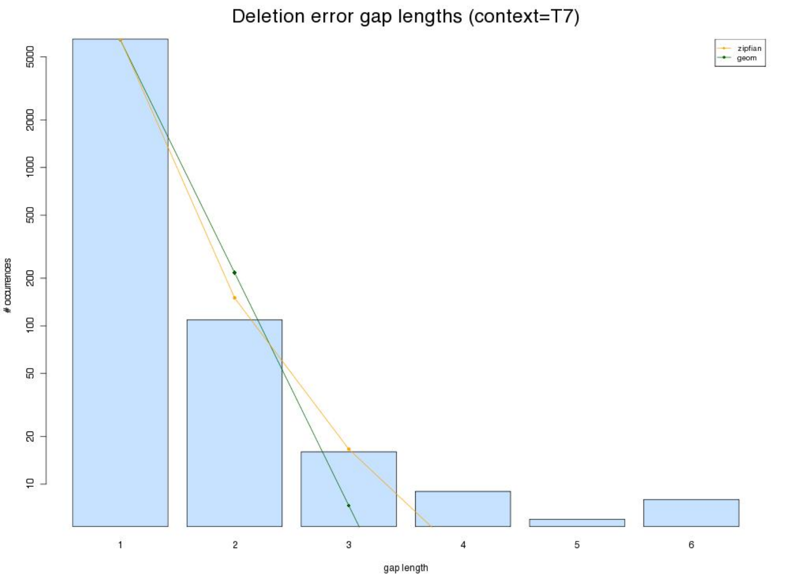	s = 5.42474147793132<br>X^2 =<br>193.190455410606<br>p-value =<br>1.0930856956806e-40	beta =<br>0.0338488473883863<br>X^2 =<br>229758.024192909<br>p-value = 0
A8	1660	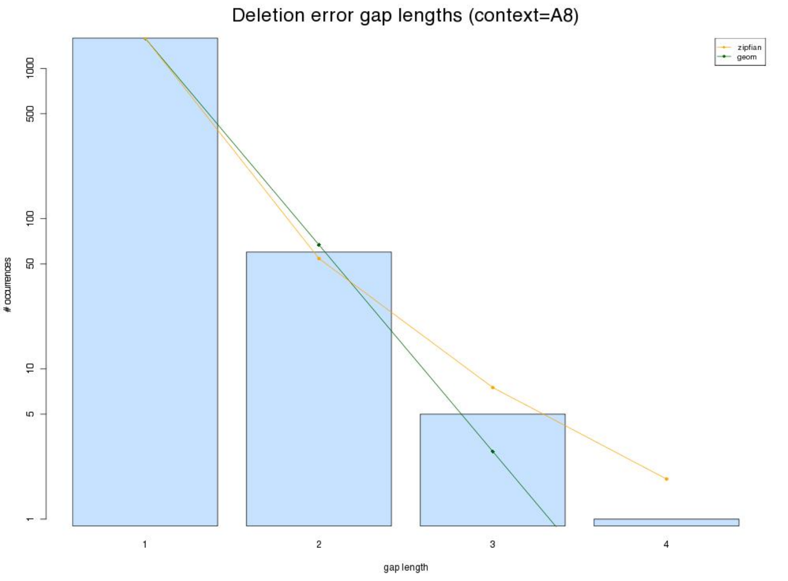	s = 4.87703260154272<br>X^2 =<br>1.83029513787374<br>p-value =<br>0.400457526638837	beta =<br>0.0421234852856318<br>X^2 =<br>8.95218804597476<br>p-value =<br>0.0113777677735931
G8	4	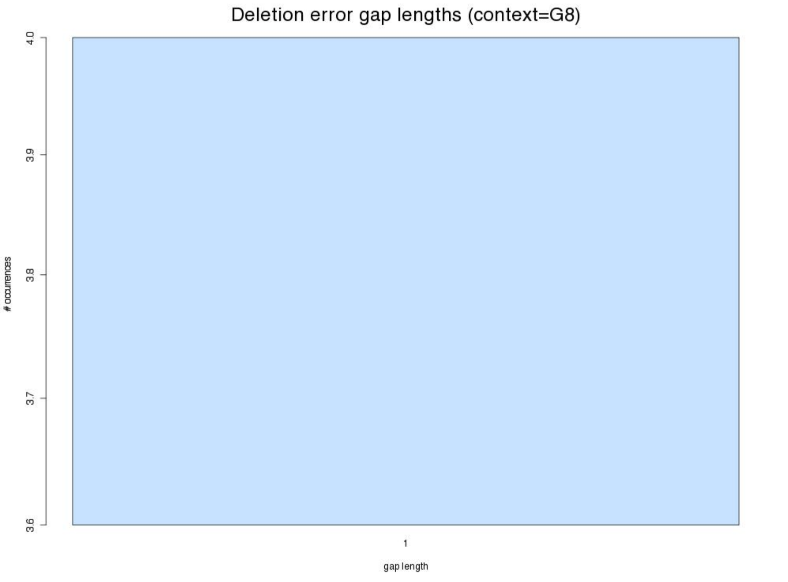	INVALID/ERROR	INVALID/ERROR

T8	2072	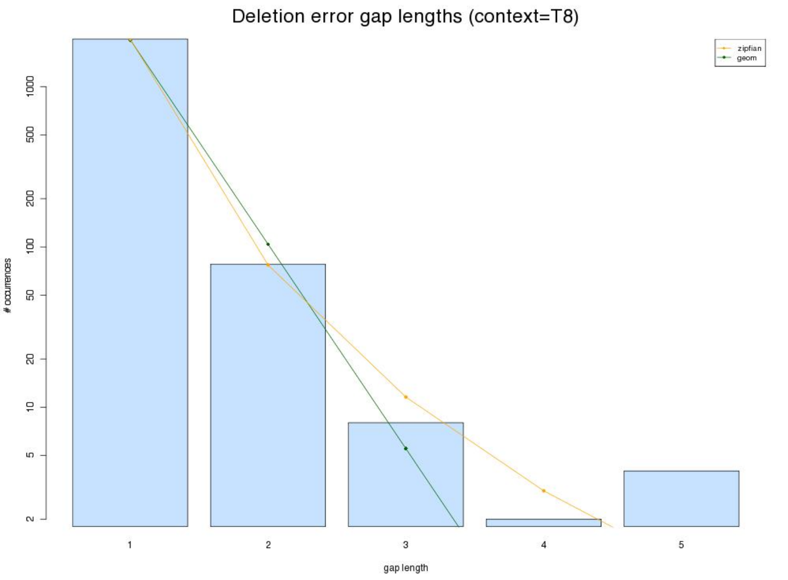	s = 4.68087274969306 X^2 = 9.6202236050093 p-value = 0.0220861854440816	beta = 0.053016453382084 X^2 = 1041.93711952551 p-value = 1.43716384369925e-225
A9	253	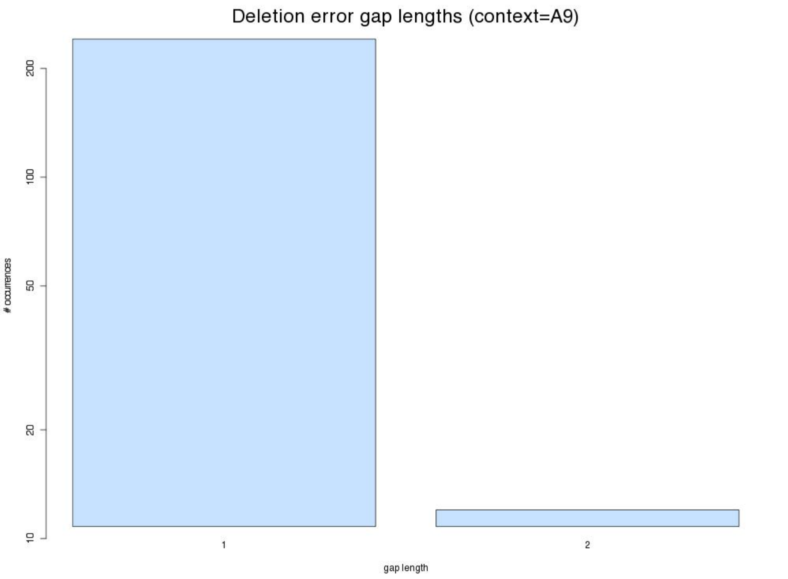	INVALID/ERROR	INVALID/ERROR
T9	205		INVALID/ERROR	INVALID/ERROR

<table>
<tr><td></td><td></td><td>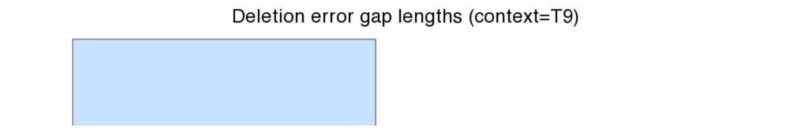</td><td></td><td></td></tr>
<tr><td>A10</td><td>43</td><td>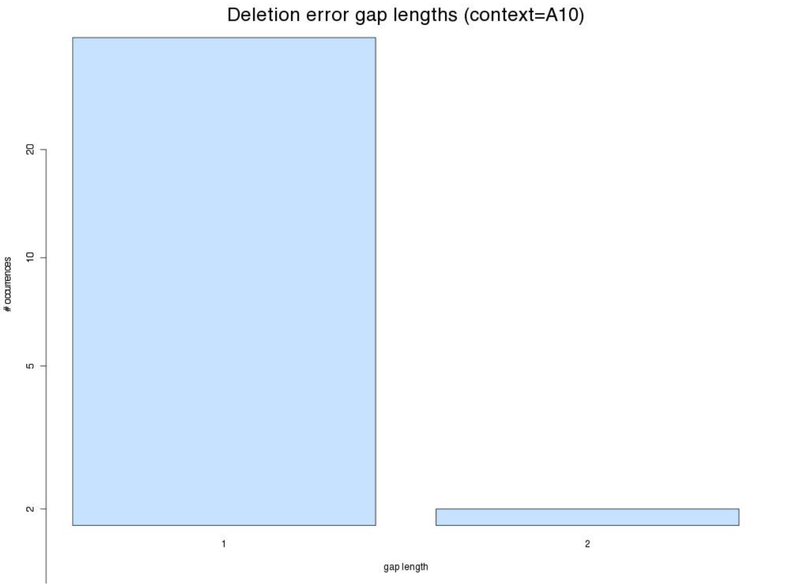</td><td>INVALID/ERROR</td><td>INVALID/ERROR</td></tr>
<tr><td>T10</td><td>24</td><td>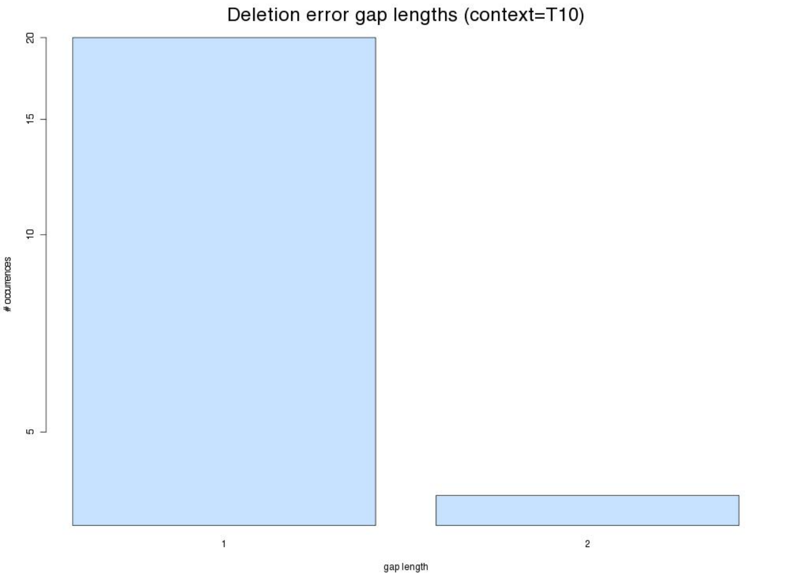</td><td>INVALID/ERROR</td><td>INVALID/ERROR</td></tr>
<tr><td></td><td></td><td></td><td></td><td></td></tr>
</table>

A11	12	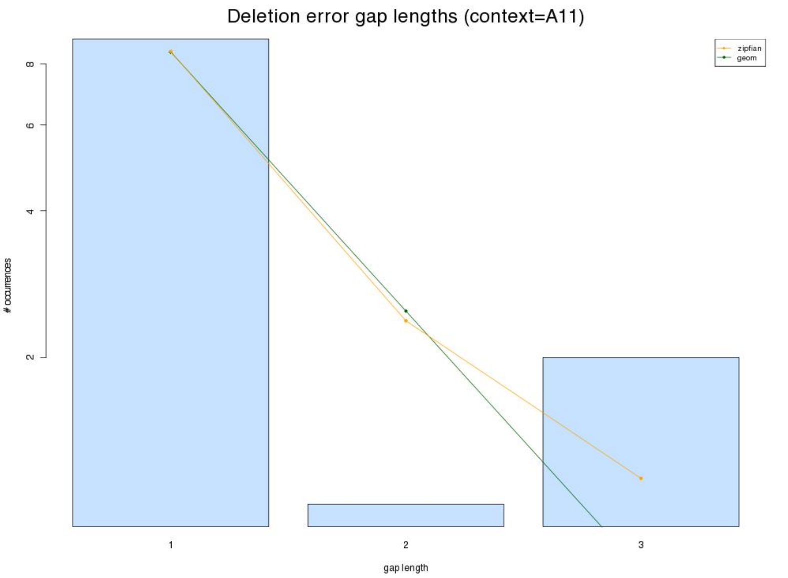	s = 1.83572058717731 X^2 = 1.49935934270401 p-value = 0.220769963822161	beta = 0.294117647058824 X^2 = 3.11746534138453 p-value = 0.0774571807894458
T11	3	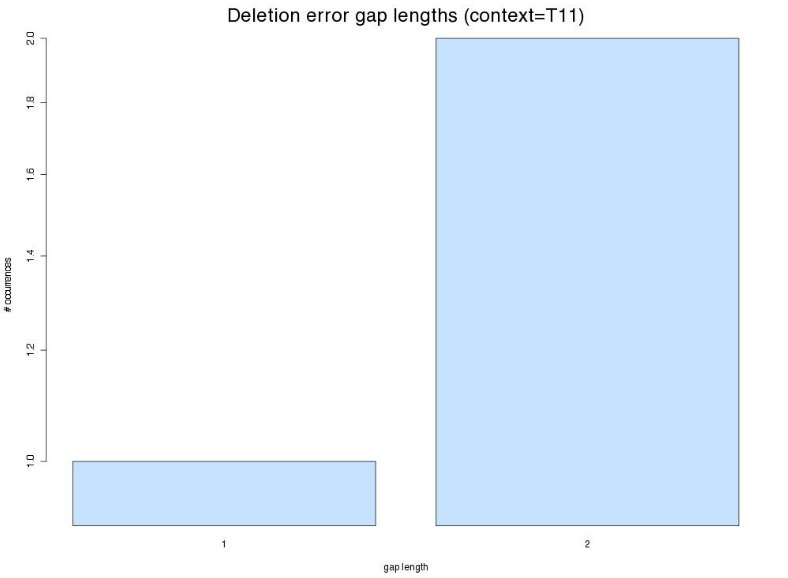	INVALID/ERROR	INVALID/ERROR
A12	8	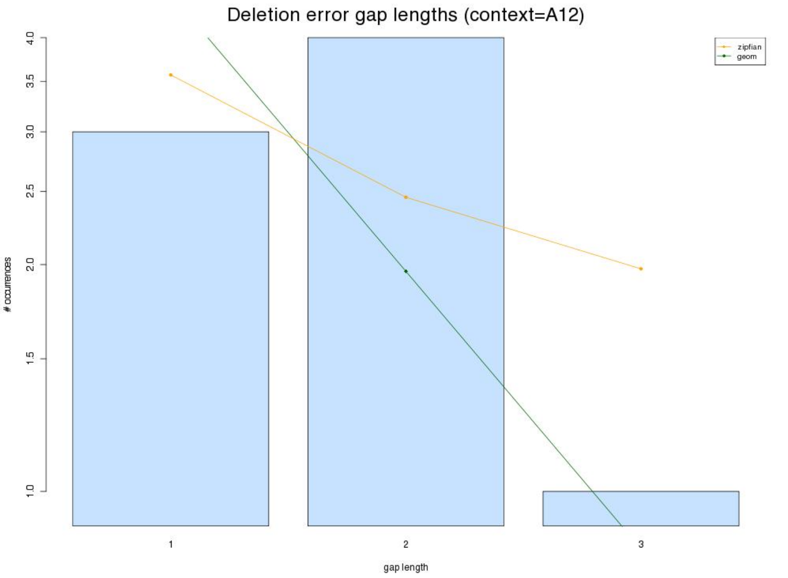	s = 0.539275867770467 X^2 = 1.54150279443063 p-value = 0.214394255775441	beta = 0.428571428571429 X^2 = 2.69665127955944 p-value = 0.100559258834669

T12	4	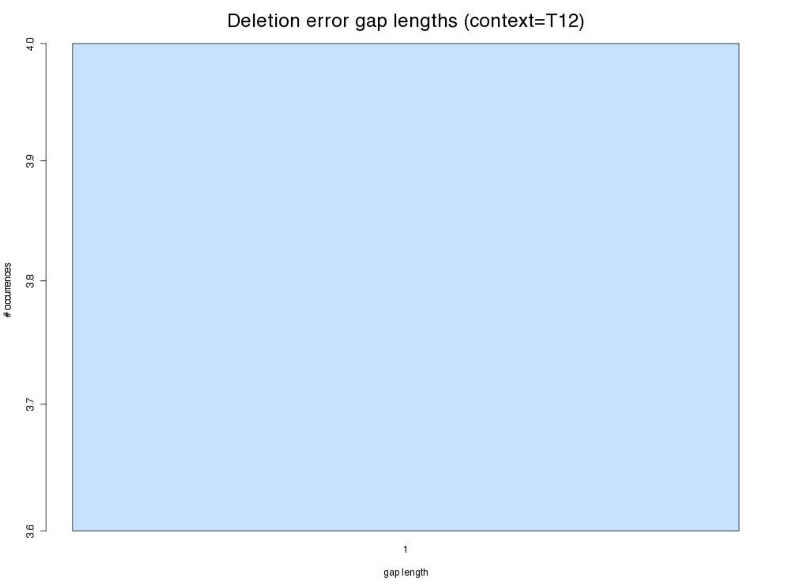	INVALID/ERROR	INVALID/ERROR
A13	7	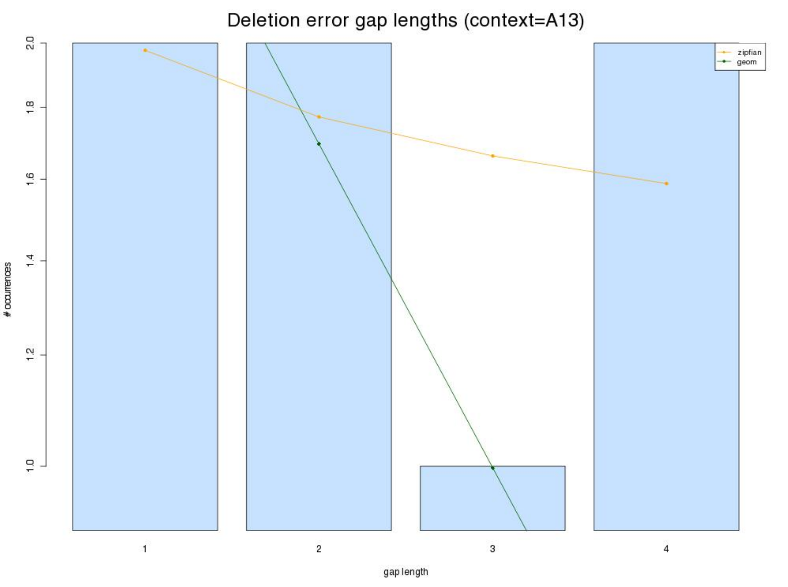	s = 0.157507175229639 X^2 = 0.399977628058061 p-value = 0.81873991142764	beta = 0.588235294117647 X^2 = 3.72952008256997 p-value = 0.154933383040224
T13	11	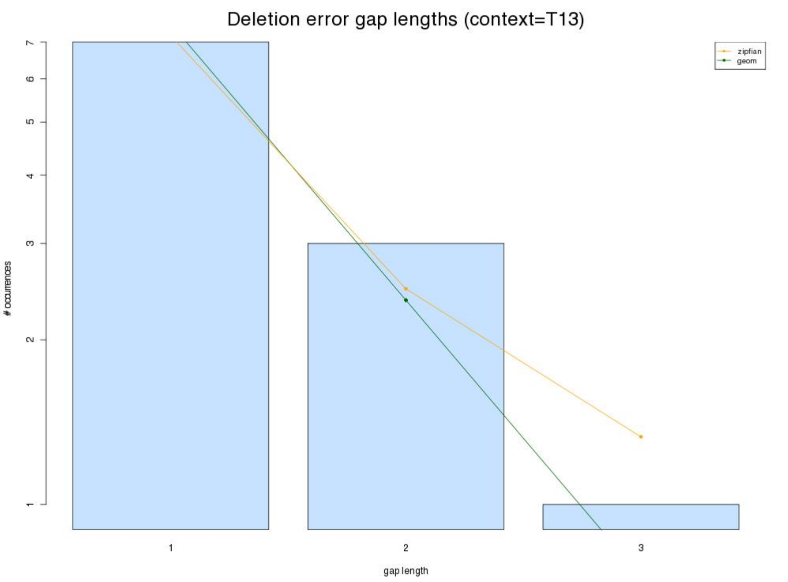	s = 1.53701796251543 X^2 = 0.196334998617163 p-value = 0.657695536506828	beta = 0.3125 X^2 = 0.305959533186983 p-value = 0.580170226064407

A14	5	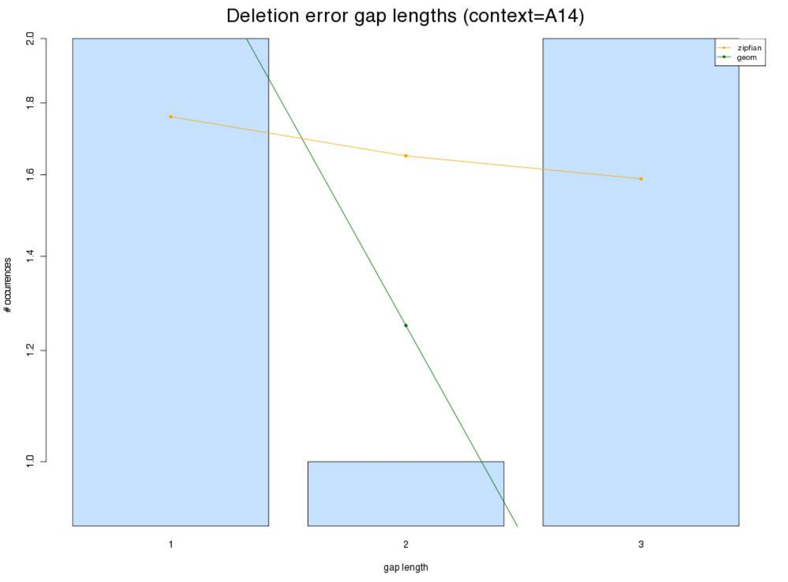	s = 0.0926542887493549 X^2 = 0.395050902693468 p-value = 0.52965630969129	beta = 0.5 X^2 = 3.175 p-value = 0.0747732118999497
T14	2	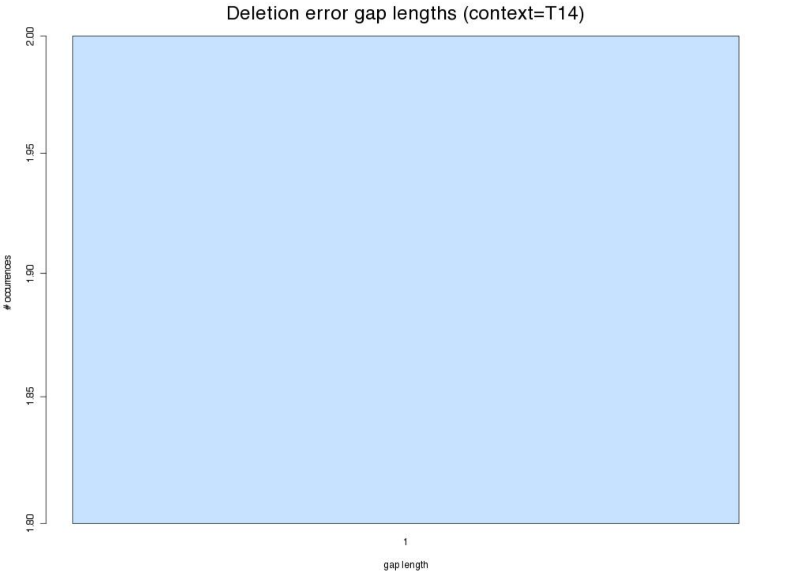	INVALID/ERROR	INVALID/ERROR

T15	4	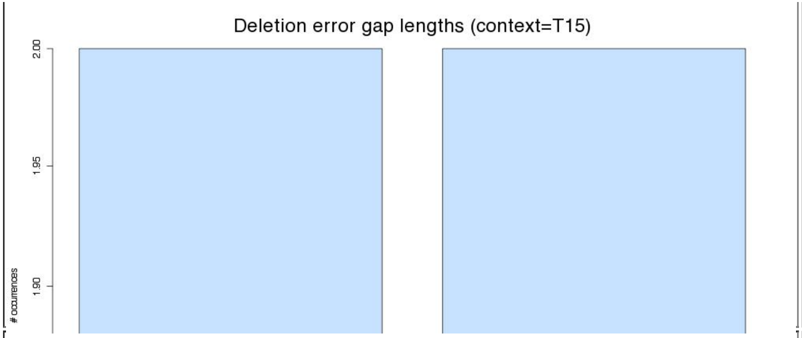	INVALID/ERROR	INVALID/ERROR
T16	4	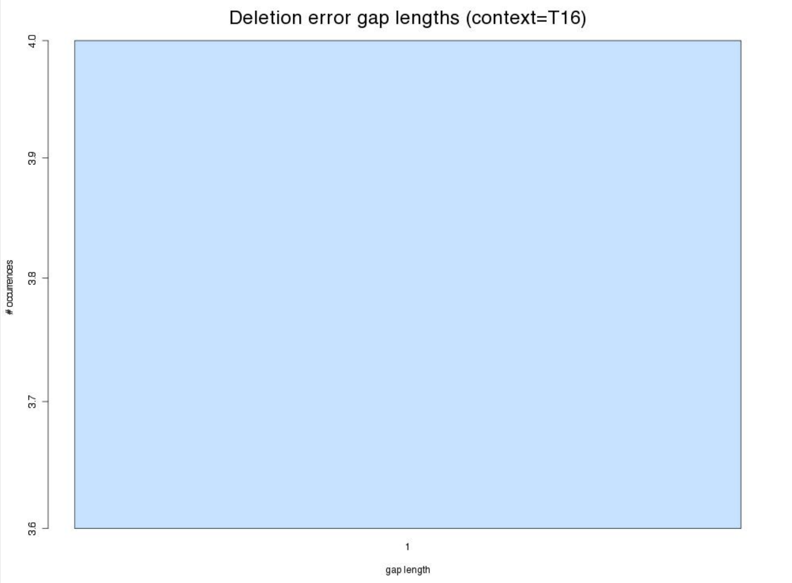	INVALID/ERROR	INVALID/ERROR
A17	3	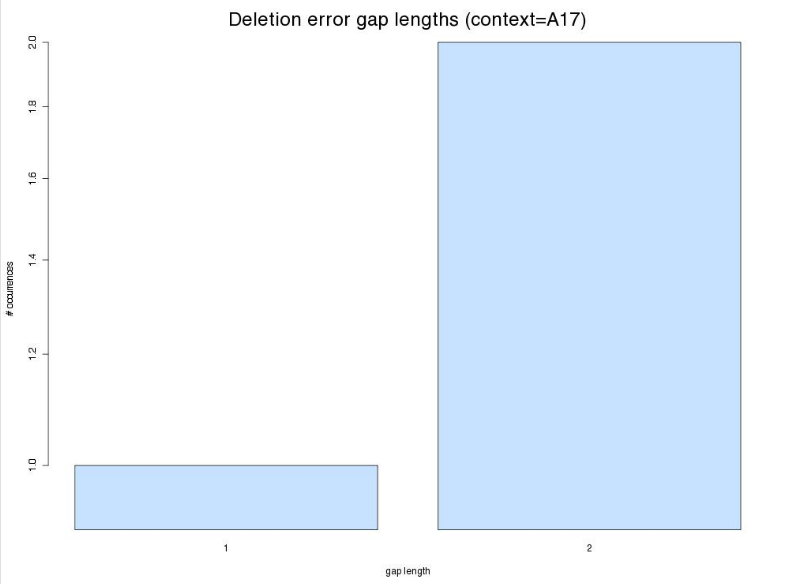	INVALID/ERROR	INVALID/ERROR
T19	3		INVALID/ERROR	INVALID/ERROR

		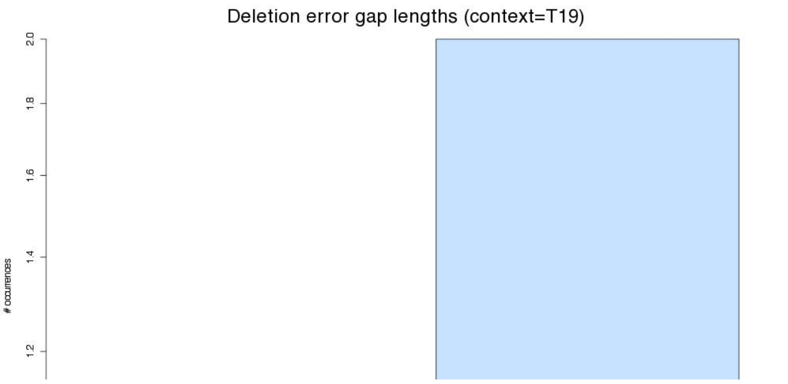		
A20	1	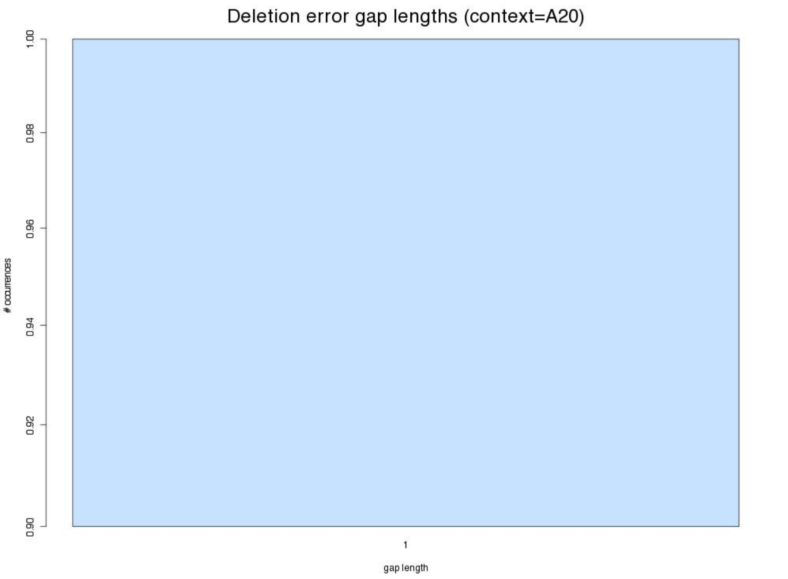	INVALID/ERROR	INVALID/ERROR
T20	5	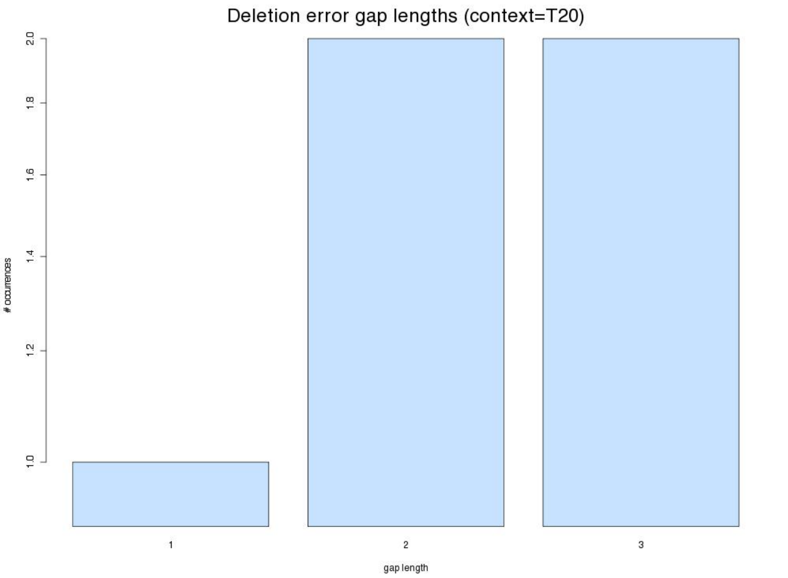	INVALID/ERROR	INVALID/ERROR

Chi square statistics

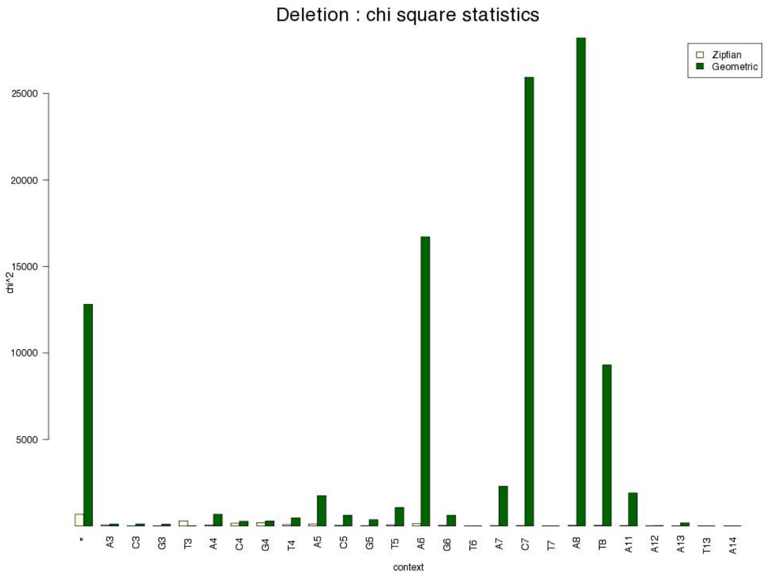

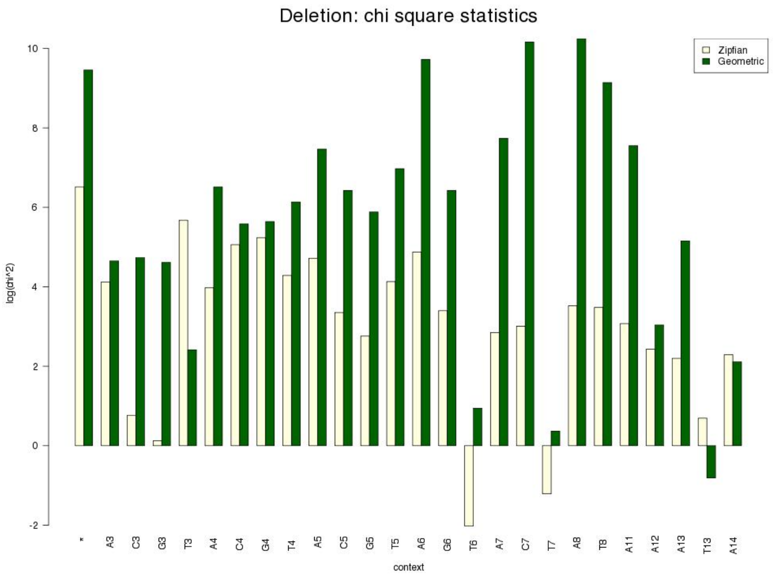

Average Pearson's residuals

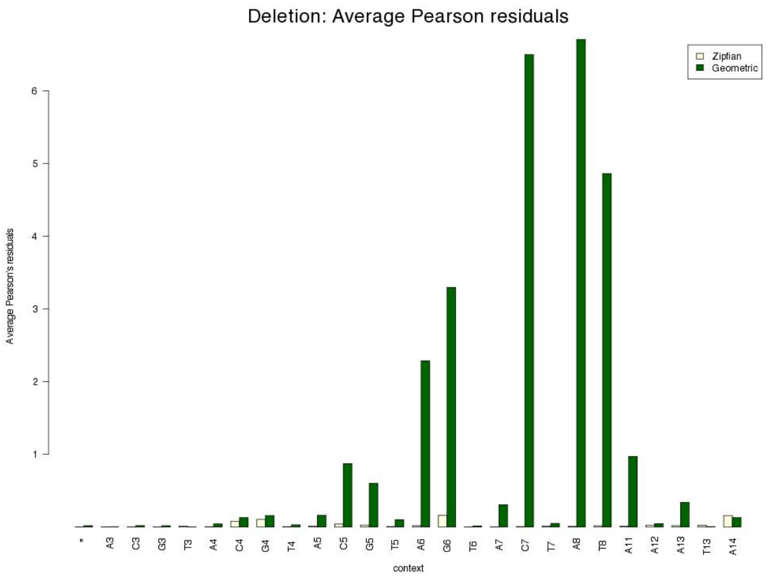
Deletion: Average Pearson residuals
Zipfian
Geometric
Average Pearson's residuals
context
* A3 C3 G3 T3 A4 C4 G4 T4 A5 C5 G5 T5 A6 G6 T6 A7 C7 T7 A8 T8 A11 A12 A13 T13 A14

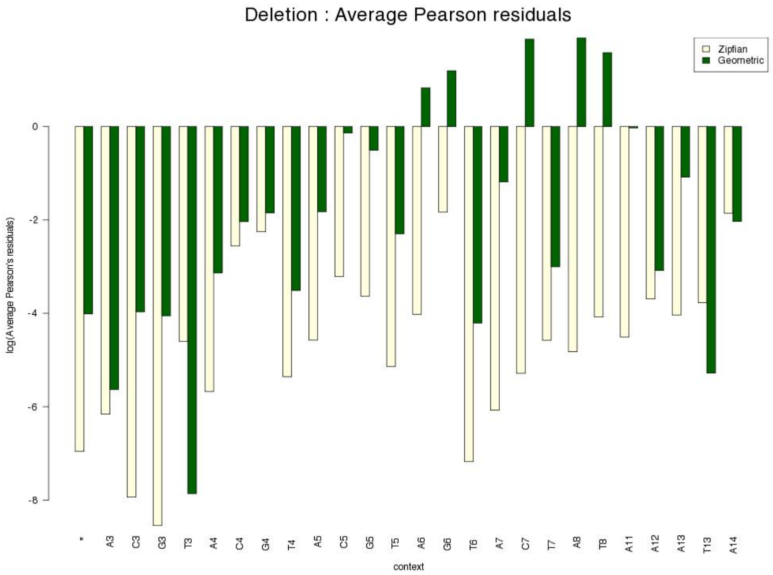
Deletion : Average Pearson residuals
Zipfian
Geometric
log(Average Pearson's residuals)
context
* A3 C3 G3 T3 A4 C4 G4 T4 A5 C5 G5 T5 A6 G6 T6 A7 C7 T7 A8 T8 A11 A12 A13 T13 A14

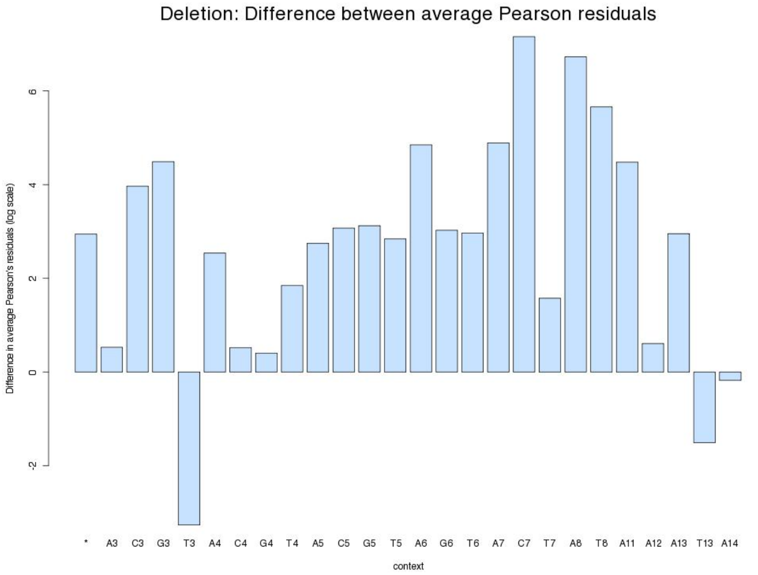
Deletion: Difference between average Pearson residuals
Difference in average Pearson's residuals (log scale)
context
* A3 C3 G3 T3 A4 C4 G4 T4 A5 C5 G5 T5 A6 G6 T6 A7 C7 T7 A8 T8 A11 A12 A13 T13 A14

Mann U Tests

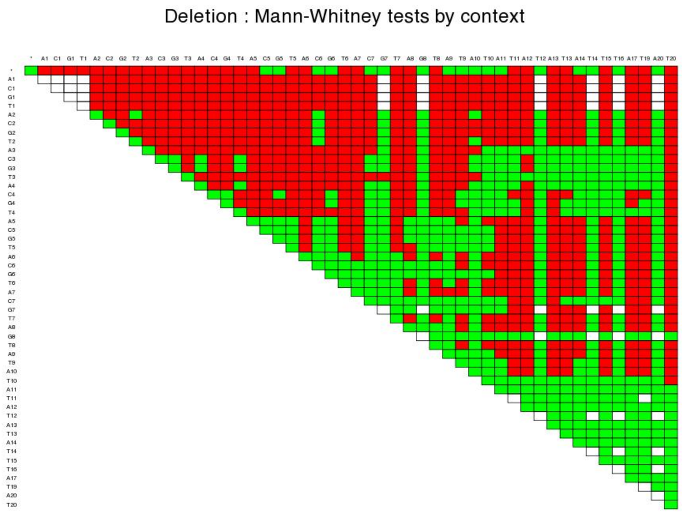

Mann U Tests by context length

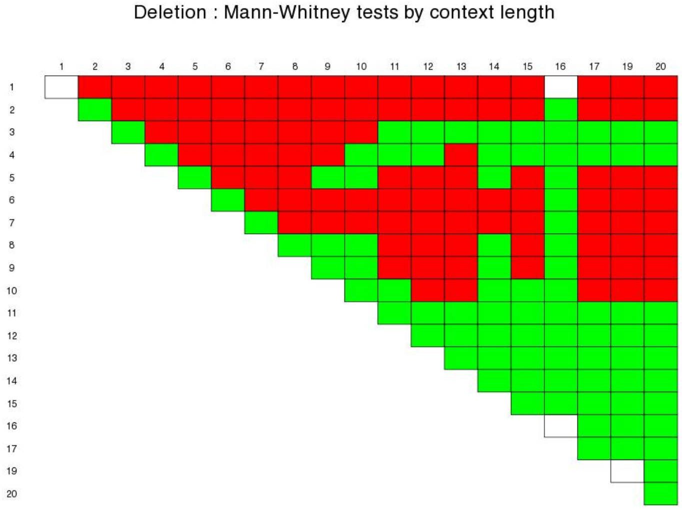

Supplement: Additional file 2 — Full results of the experiments for the Celera WGS assembler. [file 1756-0500-6-25-S2.pdf]
