# Supplementary material for: Empirical assessment of sequencing errors for high throughput pyrosequencing data
Source: BMC Res Notes. 2013 Jan 22;6:25. doi: 10.1186/1756-0500-6-25 (PMC3852801; doi:10.1186/1756-0500-6-25)

newbler Matches

HP base \ HP length	1	2	3	4	5	6	7	8	9	10	11	12	13	14	15	16	17	18	19	20	21	22	23	24
A	10112299	3780060	1394445	591882	250066	94484	27007	4569	498	61	10	9	10	4	3	7	1	2	0	1	0	0	0	1
C	9275676	1932907	270496	54226	8510	1239	175	6	0	0	0	0	0	0	0	0	0	0	0	0	0	0	0	NA
G	9325002	1918715	273077	51062	7796	1026	115	10	0	0	0	0	0	0	0	0	0	0	0	0	0	0	0	NA
T	10114600	3747686	1369516	584450	260939	101704	33958	6960	802	88	37	18	21	11	4	2	4	1	0	2	0	0	0	NA

Total HP matches: 55596277

Total match positions: 80998946

newbler Substitutions

	A	C	G	T
A	0 (0 %)	74588 (11.2 %)	63823 (9.58 %)	92787 (13.93 %)
C	26505 (3.98 %)	0 (0 %)	49970 (7.5 %)	41064 (6.17 %)
G	23442 (3.52 %)	48670 (7.31 %)	0 (0 %)	51326 (7.71 %)
T	53587 (8.05 %)	72148 (10.83 %)	68059 (10.22 %)	0 (0 %)

Total substitutions: 665969

newbler Insertion

Insertion per context

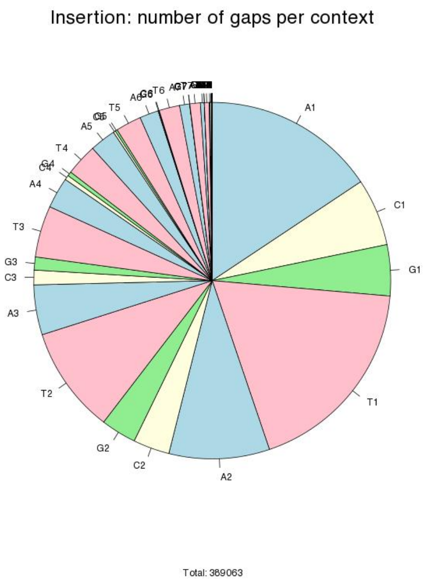

Insertion per context

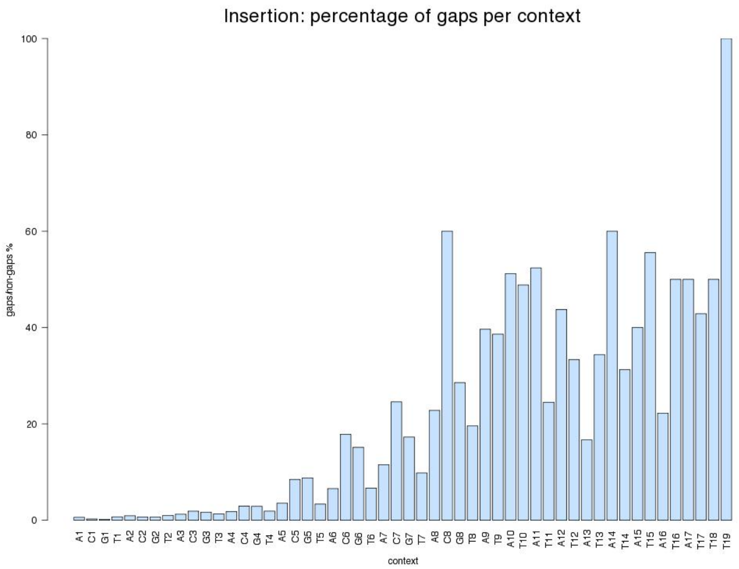

Context	#Data	Gap lengths	Zipfian fit	Geom fit
*	389077	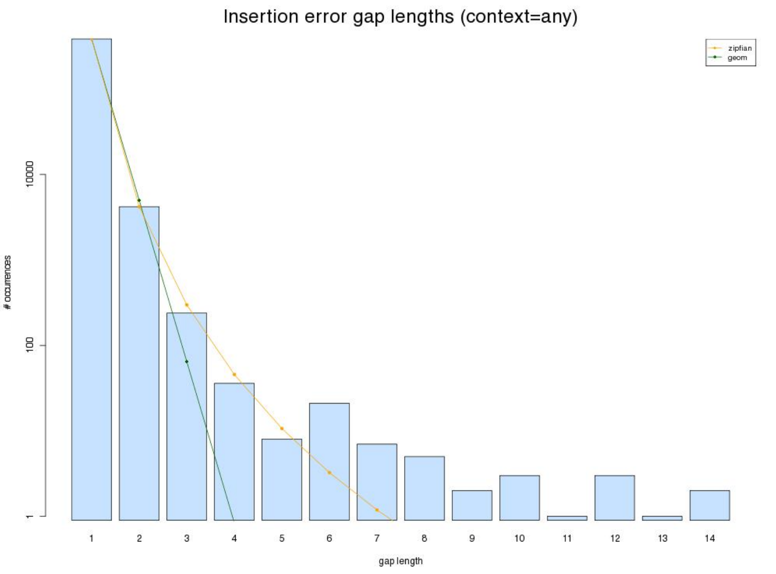	s = 6.51939809057985 X^2 = 877.746274929354 p-value = 3.44585223136766e-180	beta = 0.0129859257831130 X^2 = 35004614722711199744 p-value = 0
A1	60867	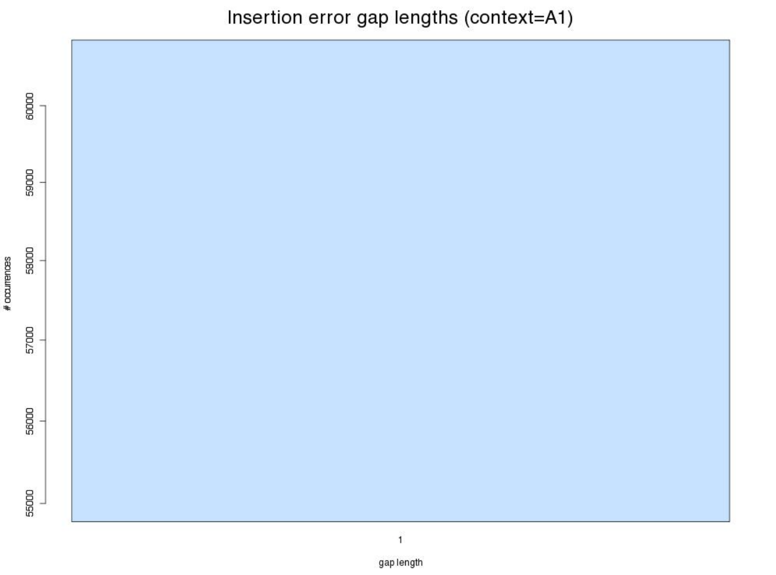	INVALID/ERROR	INVALID/ERROR

C1	23769	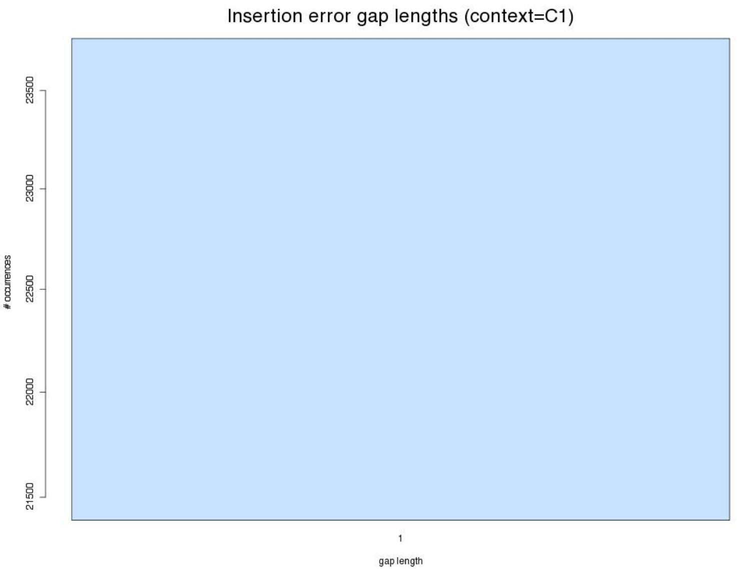	INVALID/ERROR	INVALID/ERROR
G1	18008	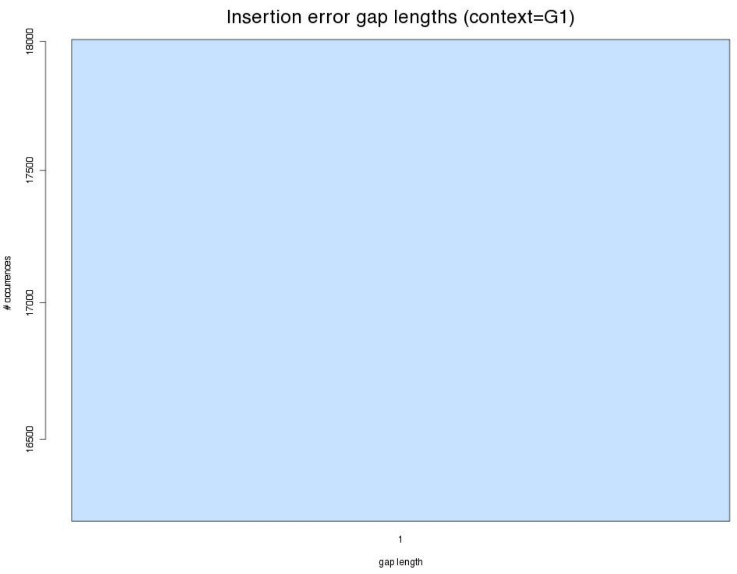	INVALID/ERROR	INVALID/ERROR
T1	71668		INVALID/ERROR	INVALID/ERROR

		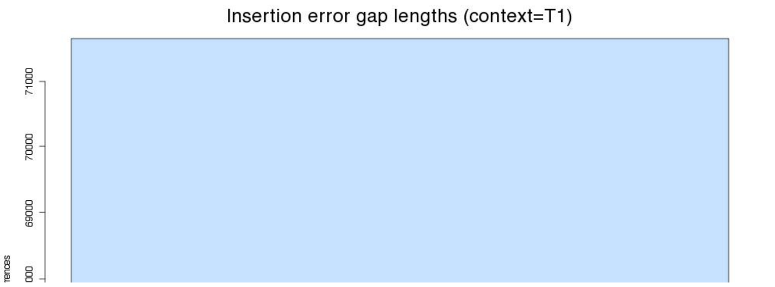		
A2	35441	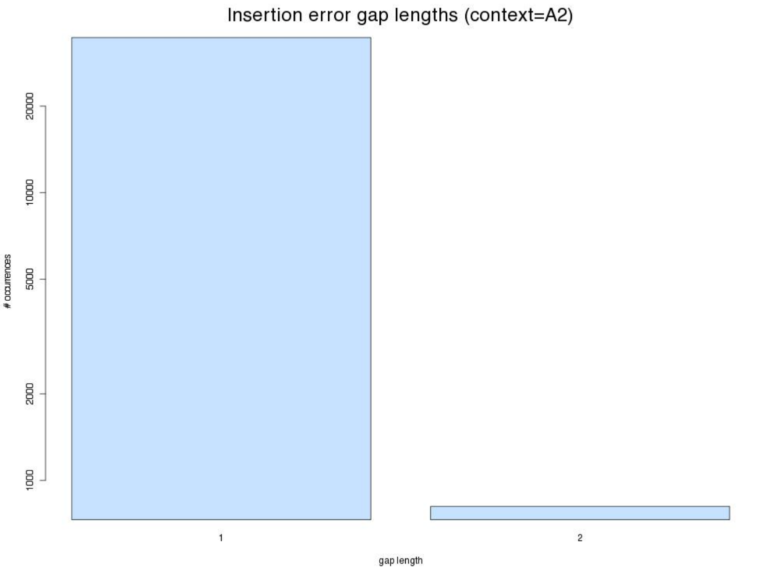	INVALID/ERROR	INVALID/ERROR
C2	12813	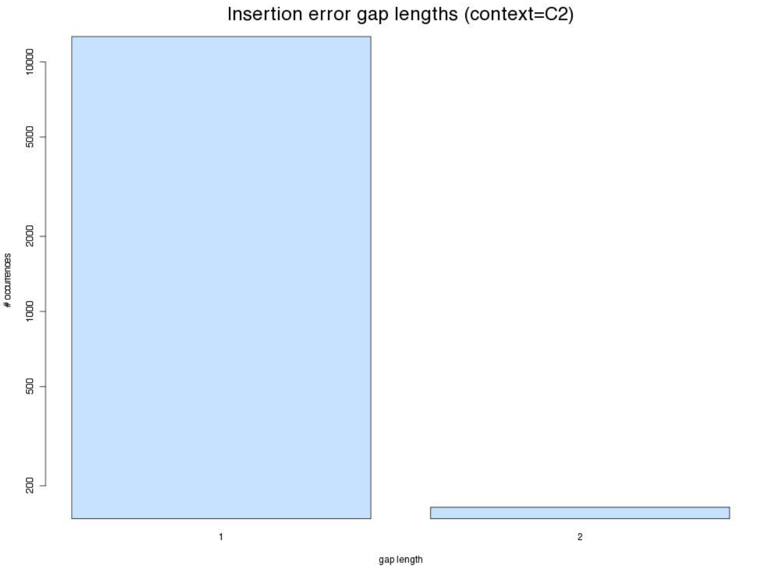	INVALID/ERROR	INVALID/ERROR

G2	12475	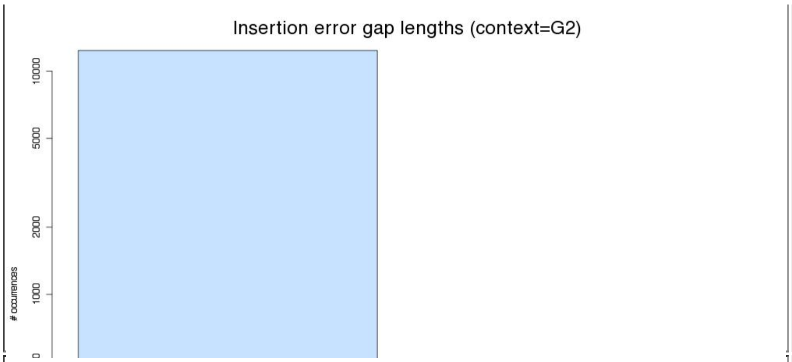	INVALID/ERROR	INVALID/ERROR
T2	37677	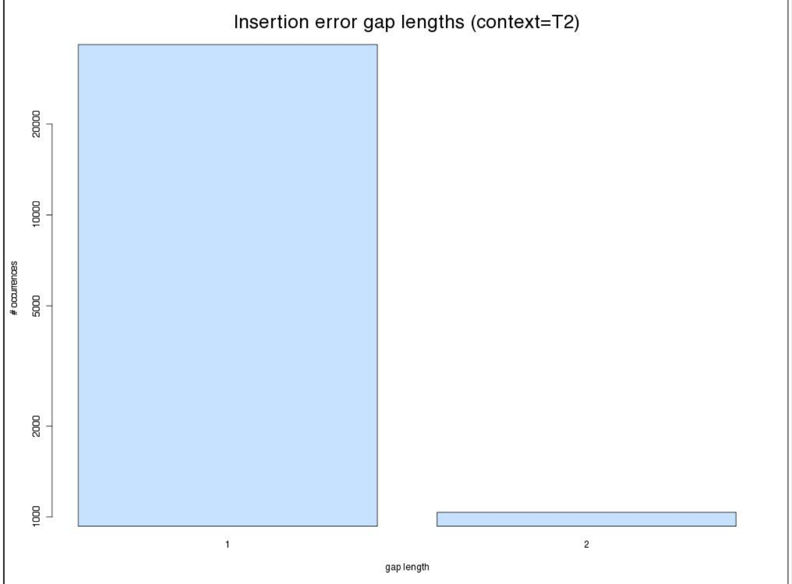	INVALID/ERROR	INVALID/ERROR
A3	17610	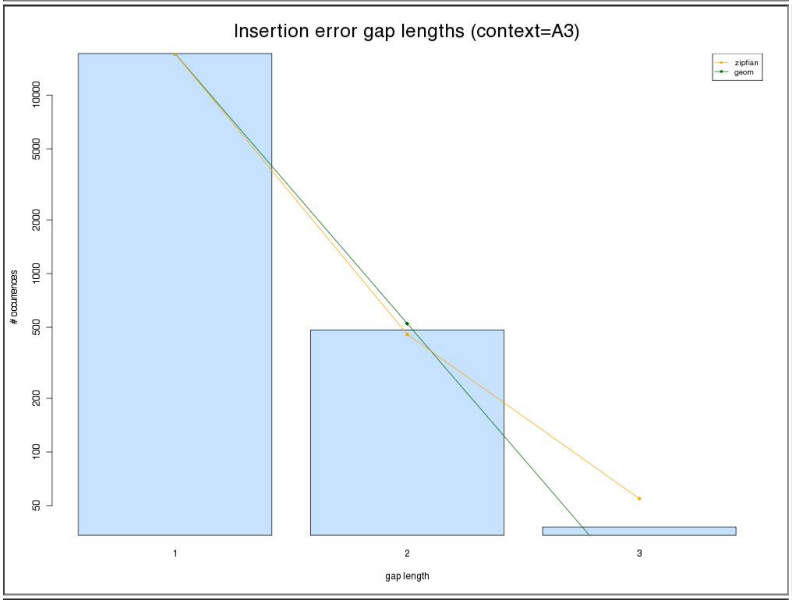	s = 5.22755075701805<br>X^2 =<br>6.71063117080714<br>p-value =<br>0.00958397966889613	beta =<br>0.0307666905168144<br>X^2 =<br>32.9376131431273<br>p-value =<br>9.51641517145777e-09
			s = 5.73916711004925	beta =

C3	5170	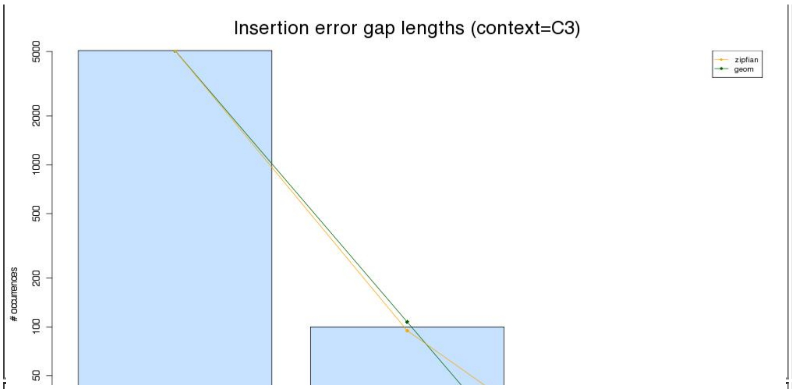	X^2 = 1.42617425706320 p-value = 0.232389817888495	0.0212040893600908 X^2 = 6.59723724054471 p-value = 0.010213713117269
G3	4599	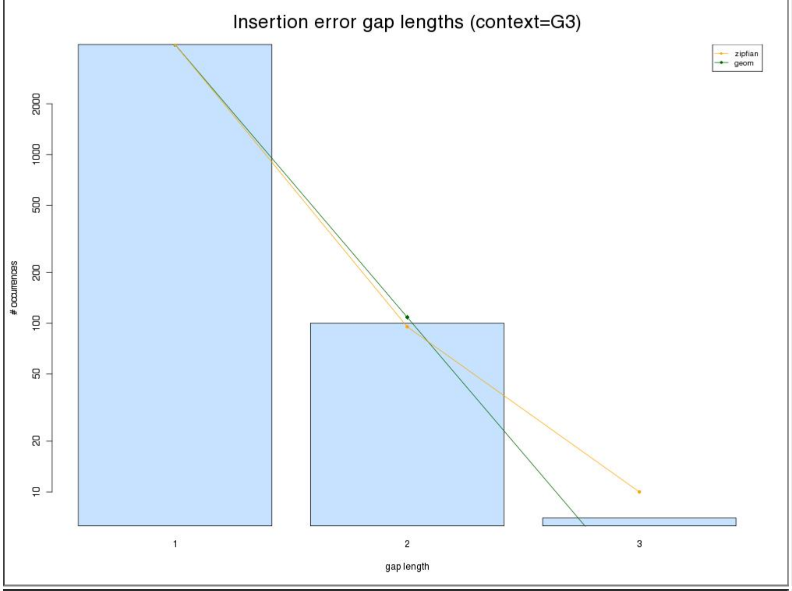	s = 5.56000151973139 X^2 = 1.13500115649519 p-value = 0.286711091735564	beta = 0.0241884150222789 X^2 = 7.96514300733158 p-value = 0.00476867233788653
T3	18172	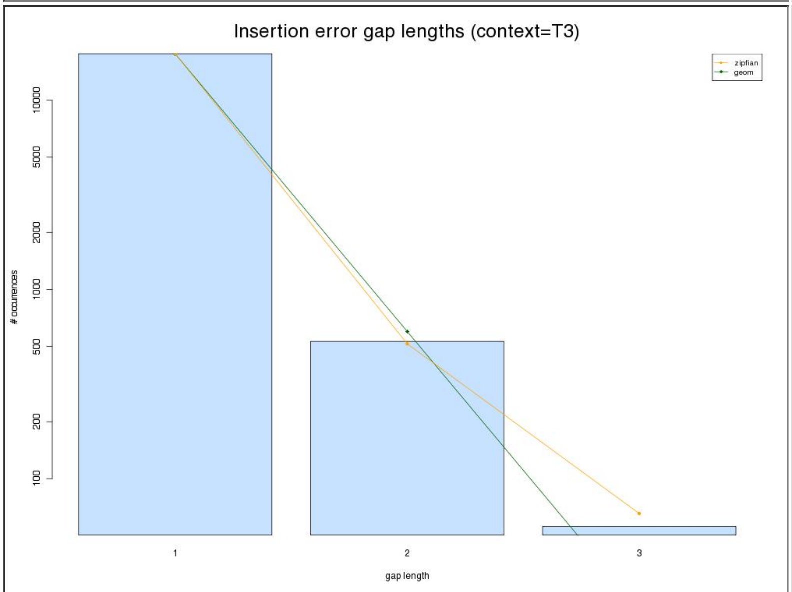	s = 5.09136183694833 X^2 = 1.81083030039386 p-value = 0.178408659637875	beta = 0.0341748604836566 X^2 = 69.4456619068442 p-value = 7.85489685251306e-17
			s = 5.88959000897921	beta =

A4	10924	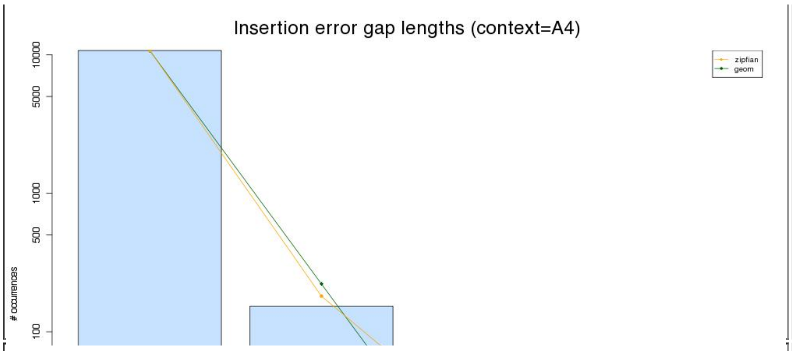	X^2 =<br>20.7852362987129<br>p-value =<br>3.06579622558528e-05	0.0207082025997312<br>X^2 =<br>357.810633349254<br>p-value =<br>2.00635687781578e-78
C4	1640	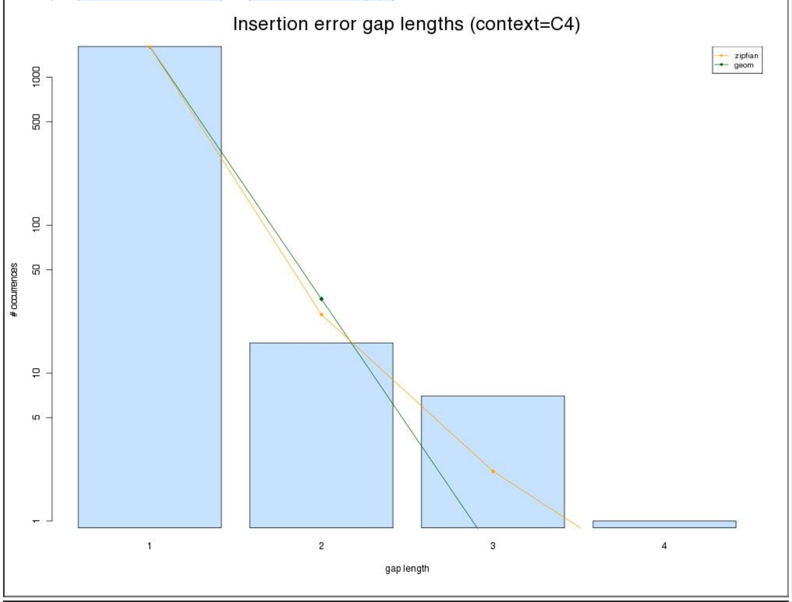	s = 6.01774467987375<br>X^2 =<br>14.9261417331973<br>p-value =<br>0.000573891119684327	beta =<br>0.0197250448296472<br>X^2 =<br>151.852576988938<br>p-value =<br>1.06079600491267e-33
G4	1516	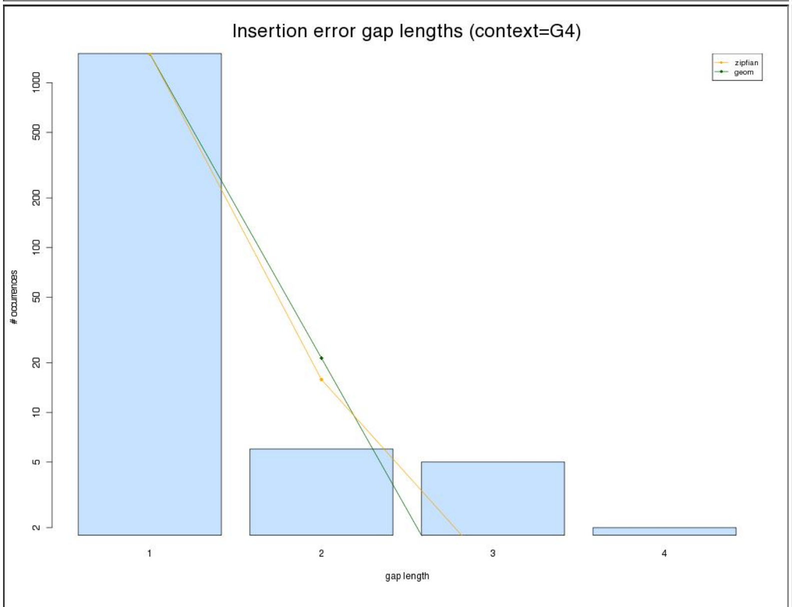	s = 6.56451405542655<br>X^2 =<br>39.9076253890933<br>p-value =<br>2.15858549207793e-09	beta =<br>0.0143042912873863<br>X^2 =<br>993.75859091435<br>p-value =<br>1.61459577227286e-216

T4	11235	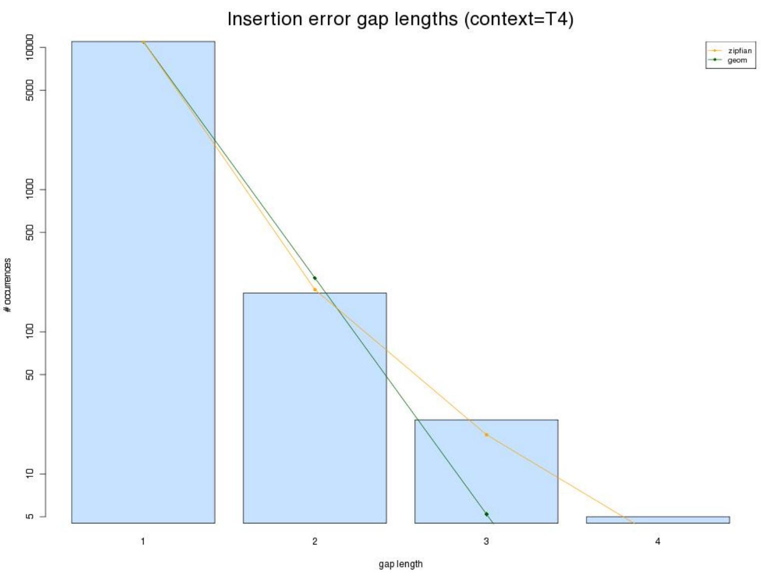	s = 5.79768276817102<br>X^2 =<br>2.59162363336367<br>p-value =<br>0.273675599707333	beta =<br>0.021767522855899<br>X^2 =<br>289.954090198064<br>p-value =<br>1.08960556043866e-63
A5	9158	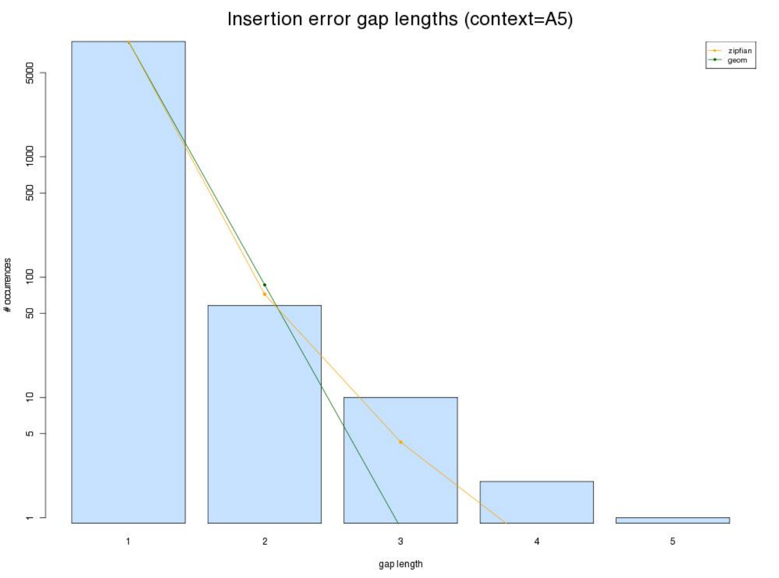	s = 6.9784881132812<br>X^2 =<br>20.5088244963975<br>p-value =<br>0.00013313238128007	beta =<br>0.00951762924507904<br>X^2 =<br>14052.3291806063<br>p-value = 0
C5	791	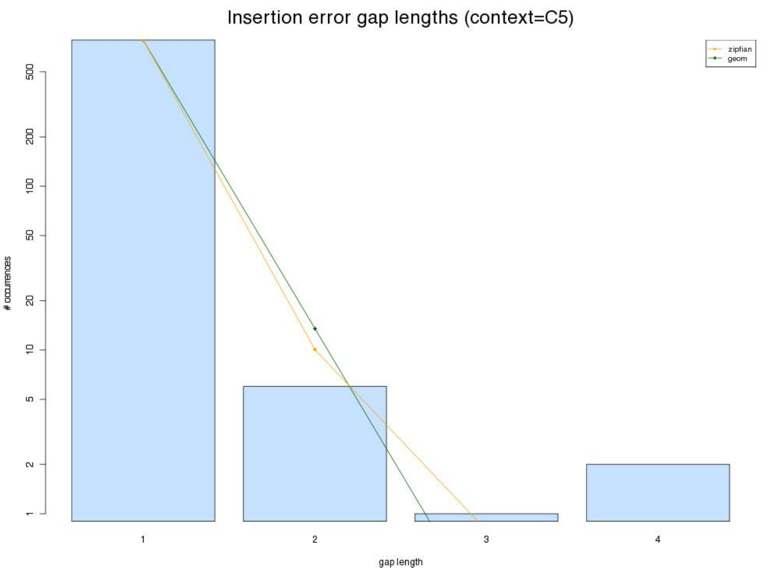	s = 6.27515053954908<br>X^2 =<br>28.5957541216234<br>p-value =<br>6.17320770478853e-07	beta =<br>0.0173913043478261<br>X^2 =<br>981.080091092807<br>p-value =<br>9.14460743624929e-<br>214

G5	756	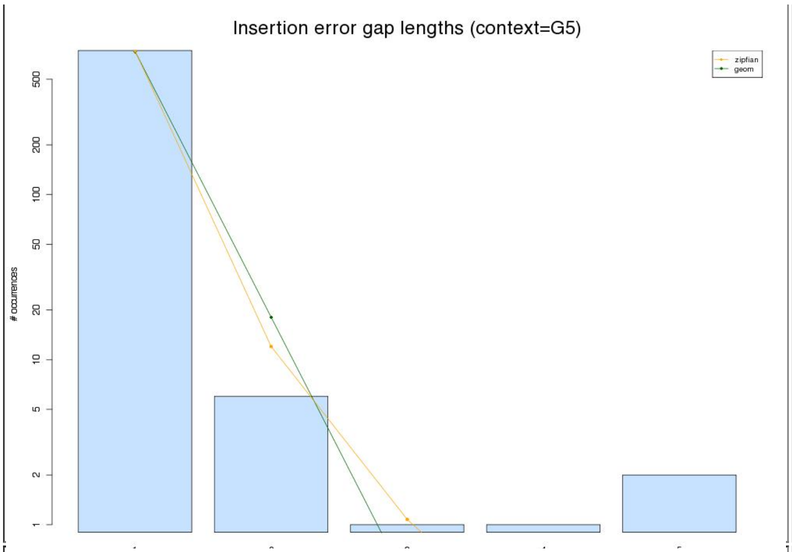	s = 5.94987448525929<br>X^2 =<br>80.0561098563668<br>p-value =<br>2.98538696130464e-17	beta =<br>0.0245161290322581<br>X^2 =<br>15109.3918488239<br>p-value = 0
T5	9036	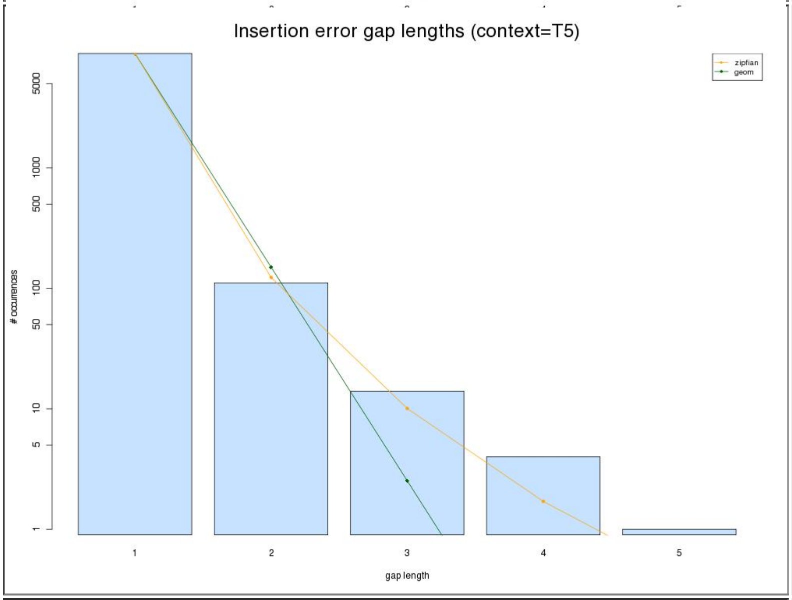	s = 6.17545065788592<br>X^2 =<br>6.58838551430183<br>p-value =<br>0.0862412169381628	beta =<br>0.0168643237950168<br>X^2 =<br>1819.43580975663<br>p-value = 0
A6	6643	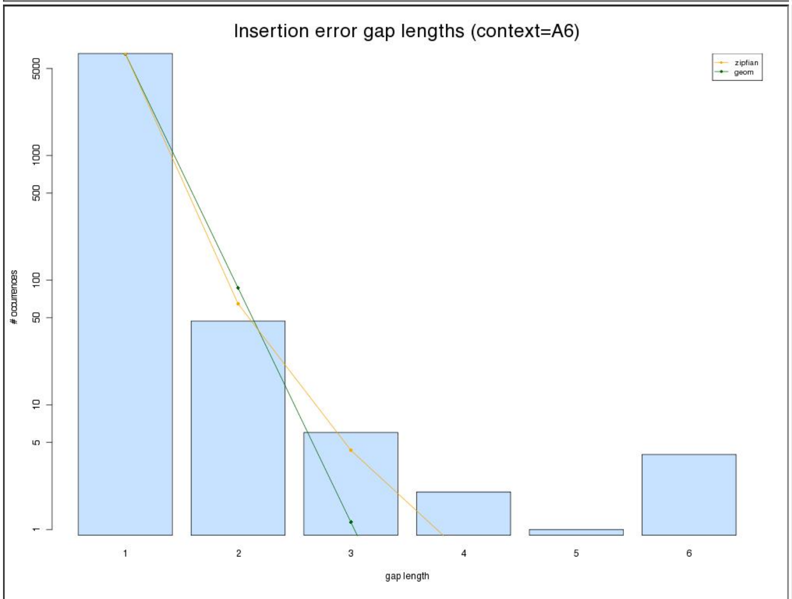	s = 6.66882616104751<br>X^2 =<br>381.995539142324<br>p-value =<br>2.1578378316663e-81	beta =<br>0.0132204396910279<br>X^2 =<br>6049027.1349865<br>p-value = 0

C6	274	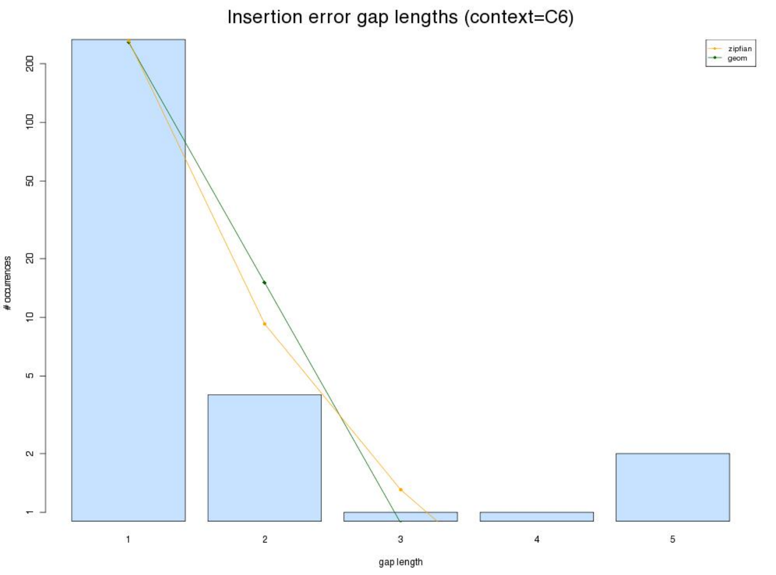	s = 4.82912365086547<br>X^2 =<br>36.695990788652<br>p-value =<br>5.33582301700687e-08	beta =<br>0.0584192439862544<br>X^2 =<br>1353.04721601992<br>p-value =<br>4.54409415520433e-<br>293
G6	187	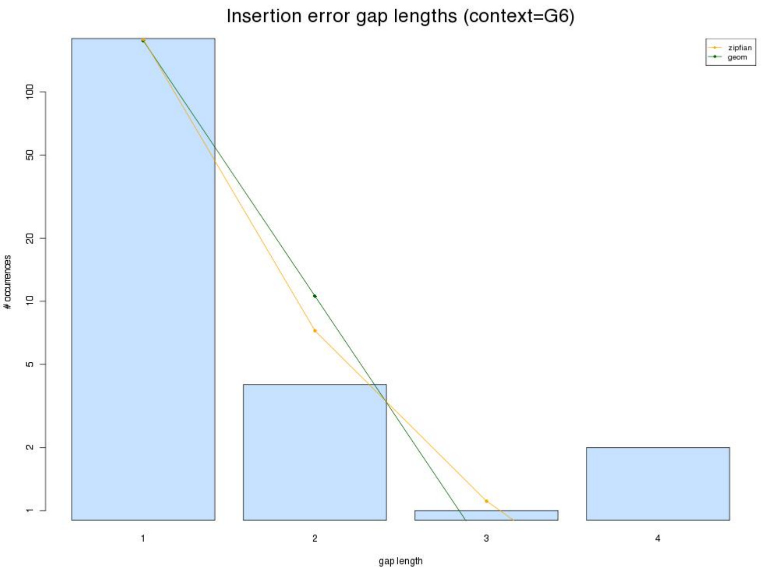	s = 4.62312722172168<br>X^2 =<br>11.3877263848567<br>p-value =<br>0.00336656213791751	beta =<br>0.0603015075376885<br>X^2 =<br>104.26439379144<br>p-value =<br>2.28704420379750e-23

T6	7257	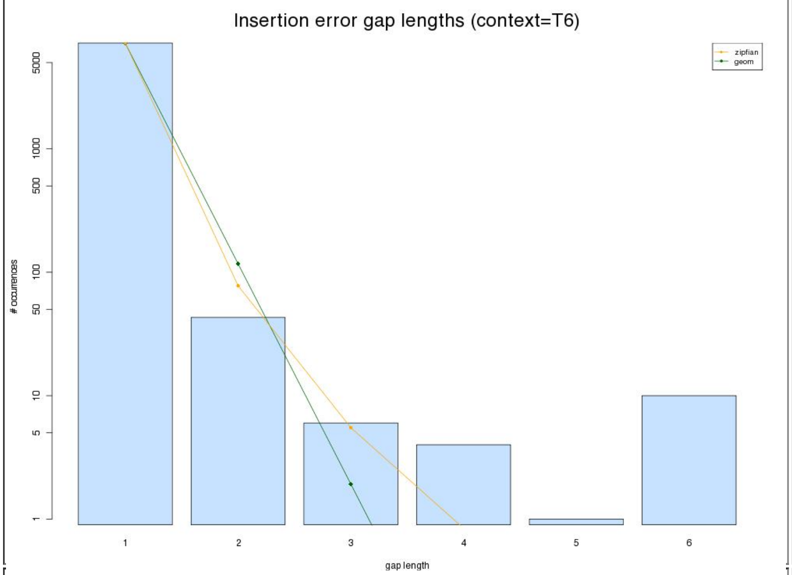	s = 6.52903164872844<br>X^2 =<br>1689.17955814413<br>p-value = 0	beta =<br>0.0164001084304689<br>X^2 =<br>11810863.9827508<br>p-value = 0
A7	3524	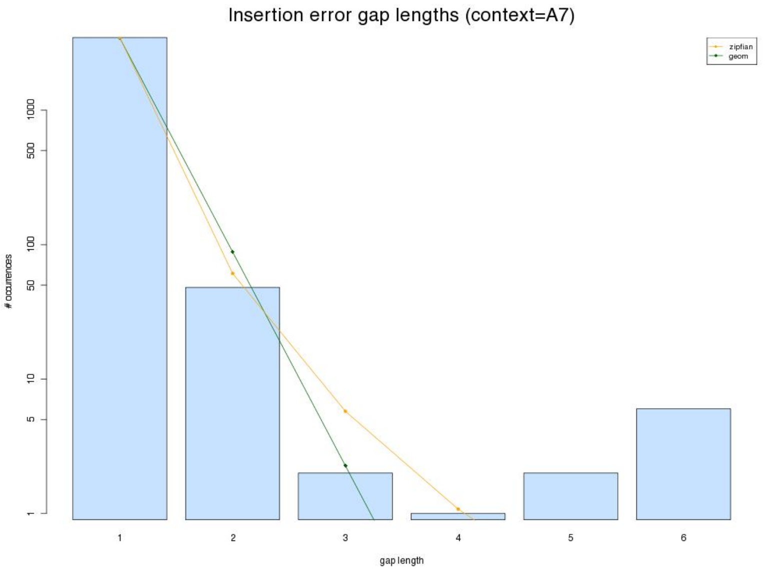	s = 5.82207914182774<br>X^2 =<br>356.63757252199<br>p-value =<br>6.46786427391924e-76	beta =<br>0.0257119159524468<br>X^2 =<br>935738.018539229<br>p-value = 0
C7	57	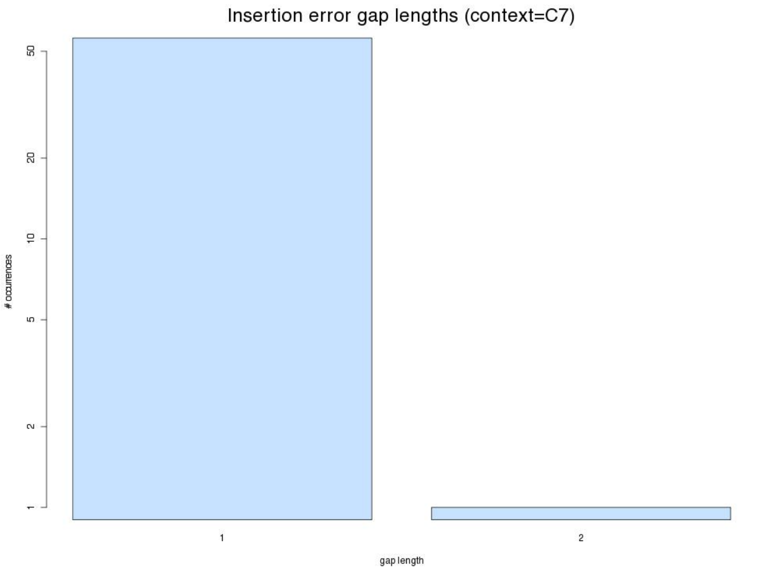	INVALID/ERROR	INVALID/ERROR

G7	24	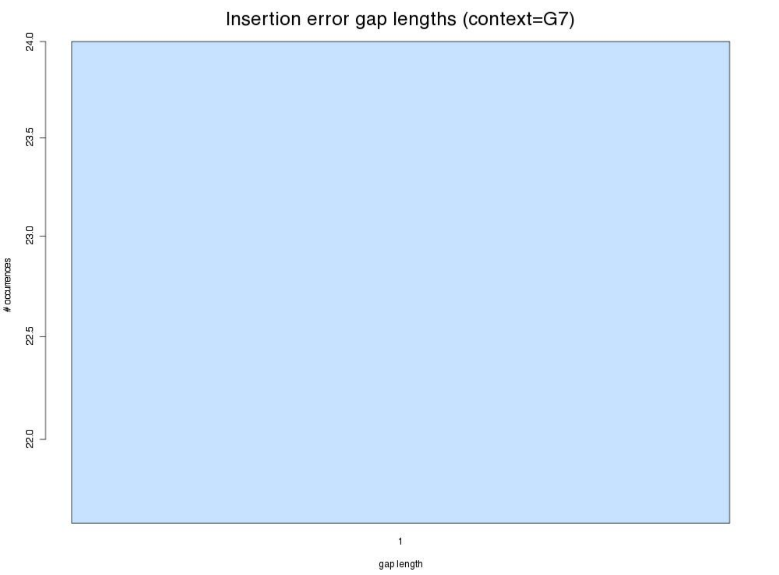	INVALID/ERROR	INVALID/ERROR
T7	3699	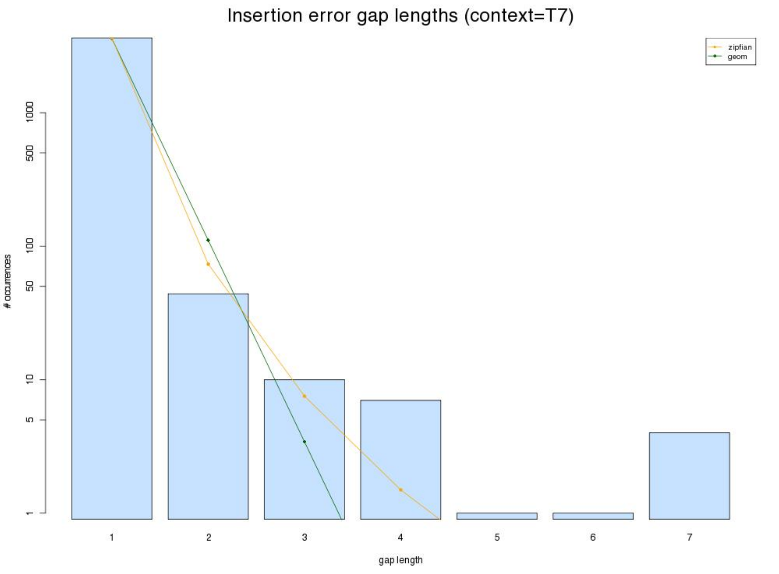	s = 5.62040209644576<br>X^2 =<br>279.256223800628<br>p-value =<br>2.87582012106314e-58	beta =<br>0.0309143306261462<br>X^2 =<br>5124130.17618897<br>p-value = 0
A8	1357	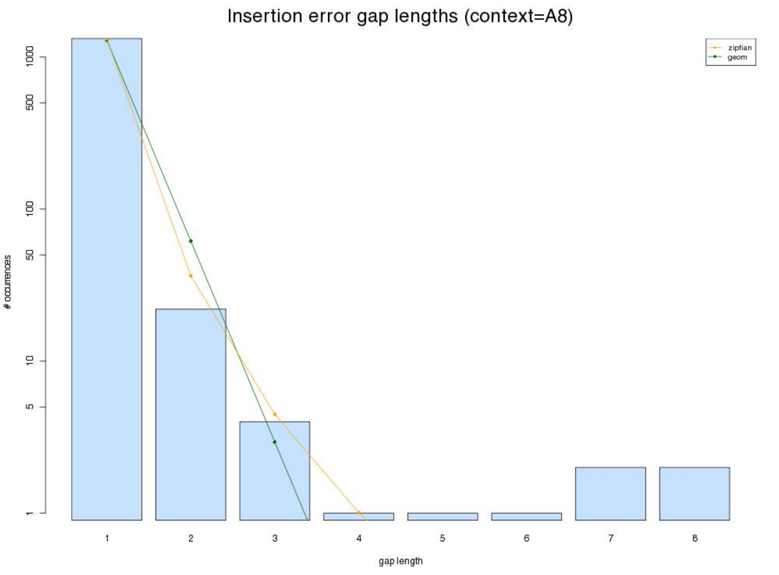	s = 5.17264169630761<br>X^2 =<br>219.903733264580<br>p-value =<br>1.09090153957858e-44	beta =<br>0.047719298245614<br>X^2 =<br>5759044.51146547<br>p-value = 0

<table>
<tr><td></td><td></td><td></td><td></td><td></td></tr>
<tr><td>C8</td><td>9</td><td>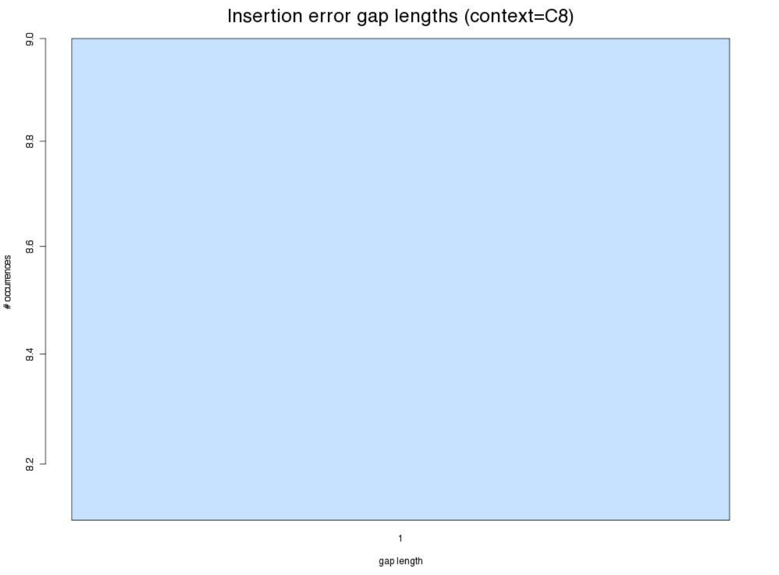
</td><td>INVALID/ERROR</td><td>INVALID/ERROR</td></tr>
<tr><td>G8</td><td>4</td><td>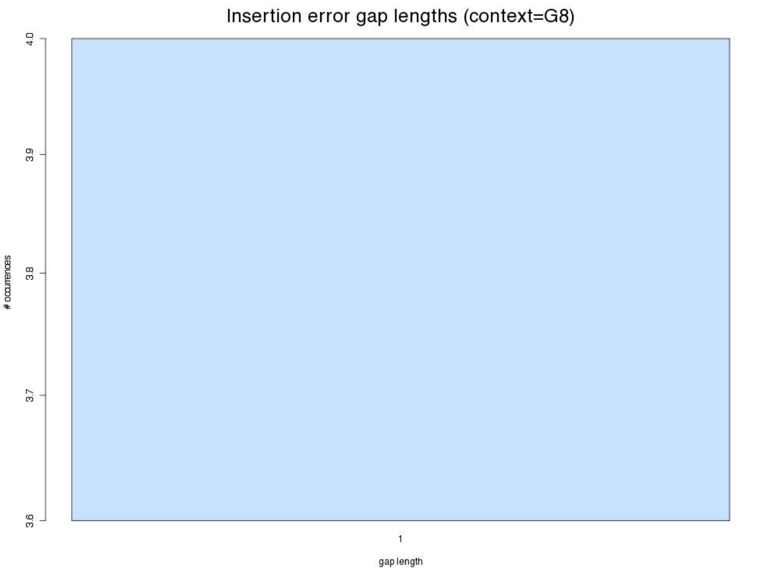
</td><td>INVALID/ERROR</td><td>INVALID/ERROR</td></tr>
<tr><td>T8</td><td>1693</td><td>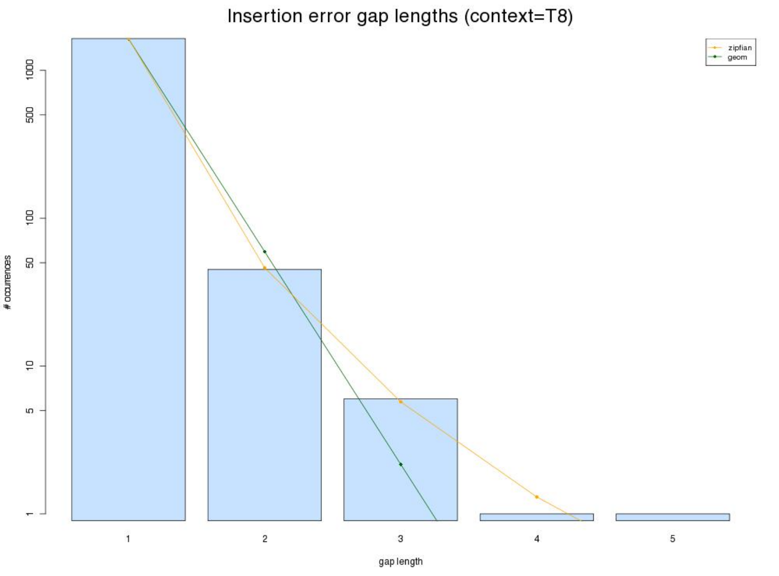
</td><td>s = 5.14929370914316
X^2 =
0.950640382989156
p-value =
0.813193053063198</td><td>beta =
0.0364257256687536
X^2 =
367.305036556666
p-value =
2.66904451545112e-79</td></tr>
<tr><td></td><td></td><td></td><td></td><td></td></tr>
</table>

A9	335	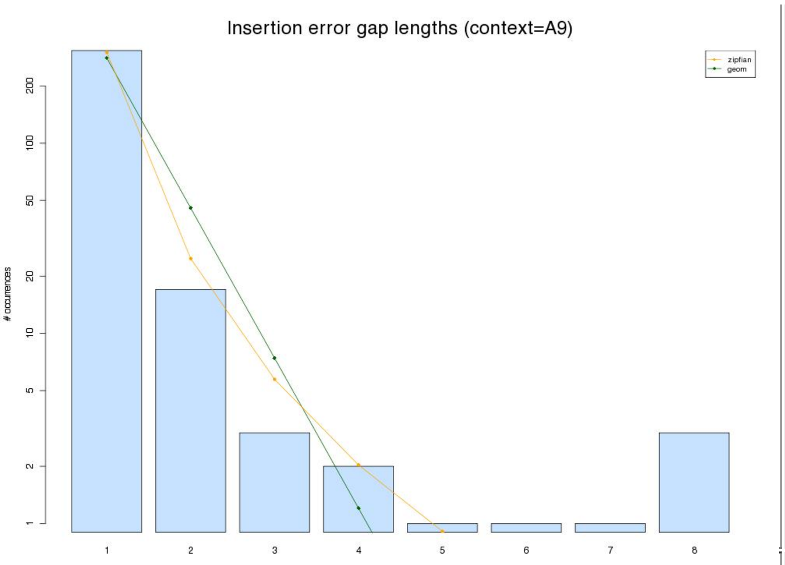	s = 3.60428937456304<br>X^2 =<br>54.4275692085391<br>p-value =<br>6.04841321290567e-10	beta = 0.1625<br>X^2 =<br>10963.0473943865<br>p-value = 0
T9	505	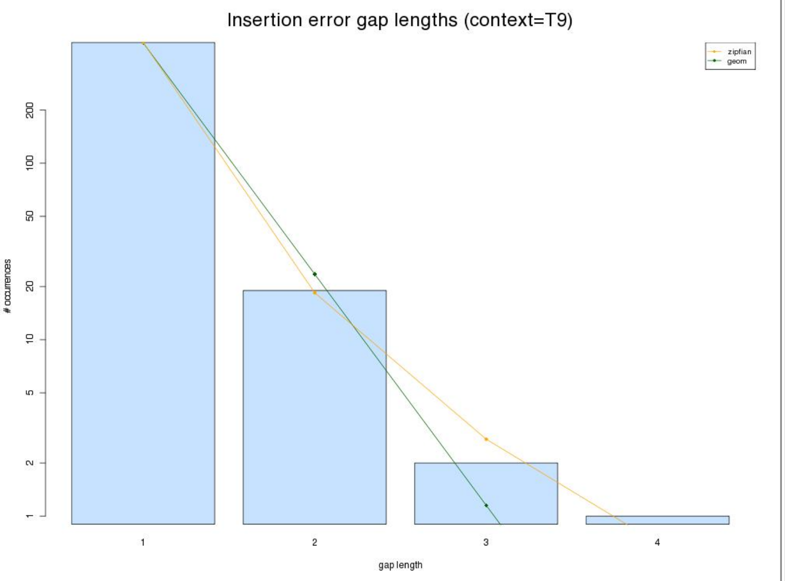	s = 4.71174780885746<br>X^2 =<br>0.336821858718806<br>p-value =<br>0.845006525339594	beta =<br>0.0489642184557439<br>X^2 =<br>17.3013082915693<br>p-value =<br>0.000175012327128963
A10	74	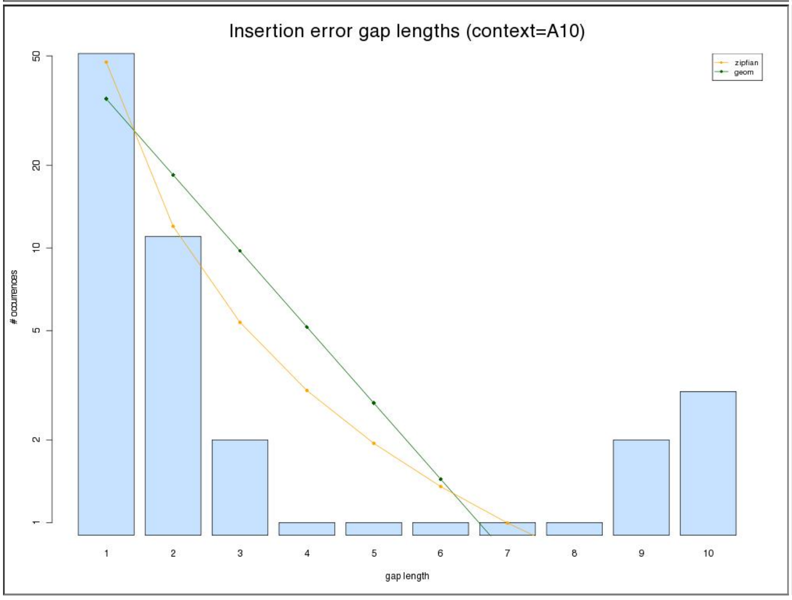	s = 1.98544444700454<br>X^2 =<br>20.4711674831067<br>p-value =<br>0.00869312636262025	beta =<br>0.528662420382166<br>X^2 =<br>111.264248688278<br>p-value =<br>2.09277970097283e-20

T10	84	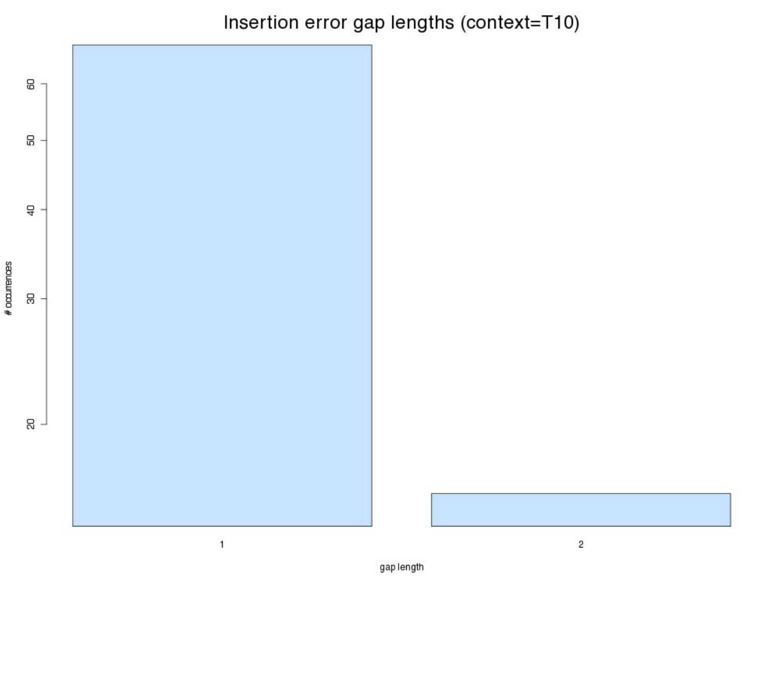	INVALID/ERROR	INVALID/ERROR
A11	11	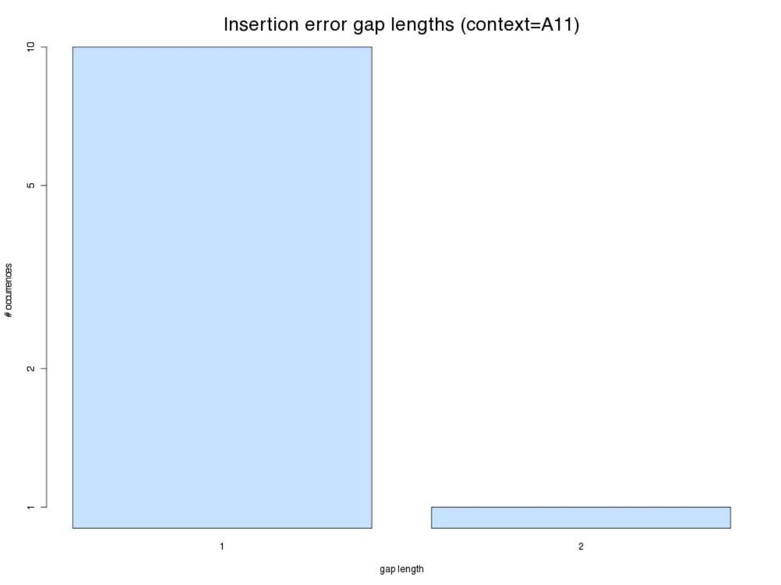	INVALID/ERROR	INVALID/ERROR
T11	12	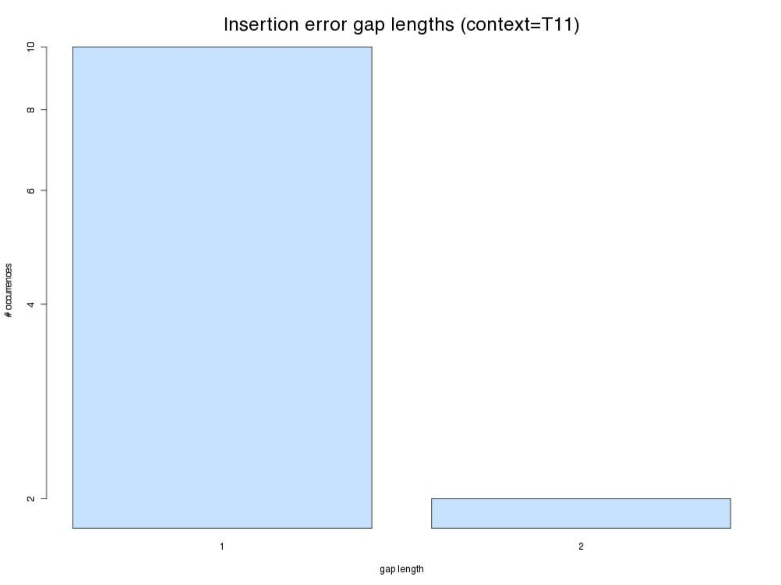	INVALID/ERROR	INVALID/ERROR

A12	19	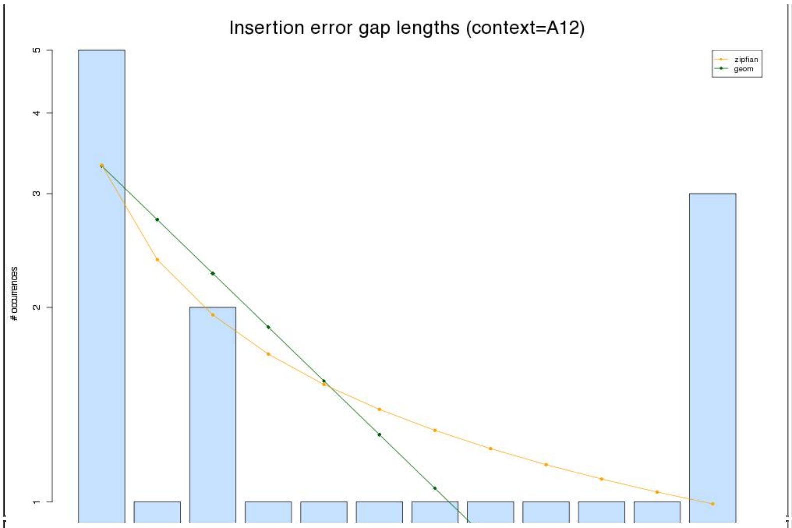	s = 0.48582025011493<br>X^2 =<br>6.39752241617023<br>p-value =<br>0.780833096790237	beta =<br>0.825688073394495<br>X^2 =<br>20.3430308947208<br>p-value =<br>0.0261701386048254
T12	9	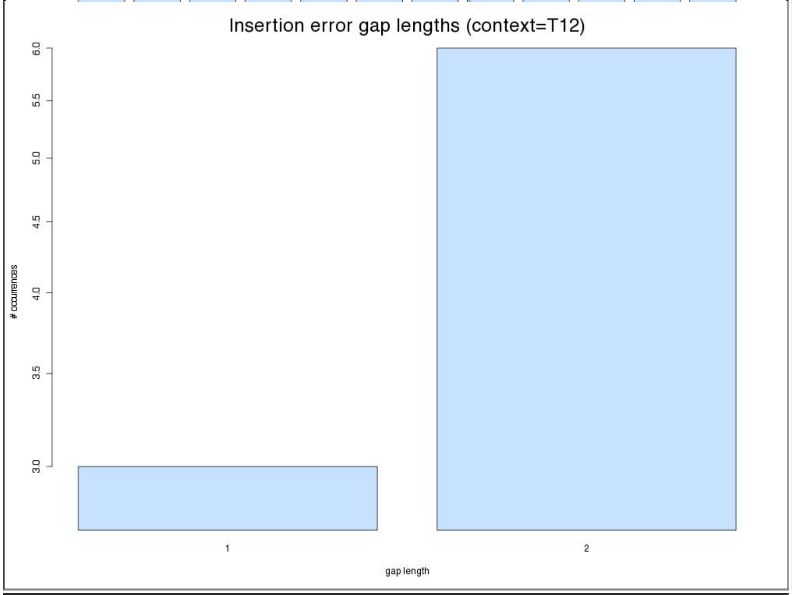	INVALID/ERROR	INVALID/ERROR
A13	6	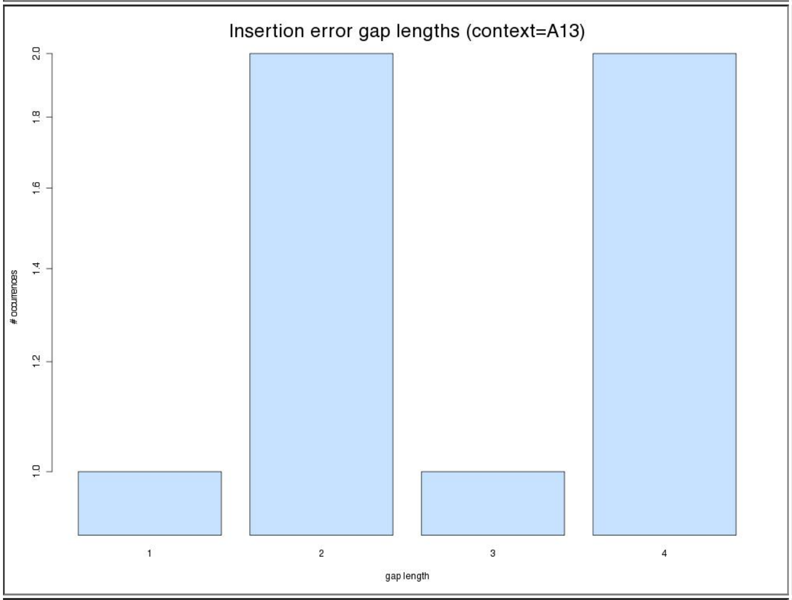	INVALID/ERROR	INVALID/ERROR
			s = 0.726652312800867	beta =

T13	17	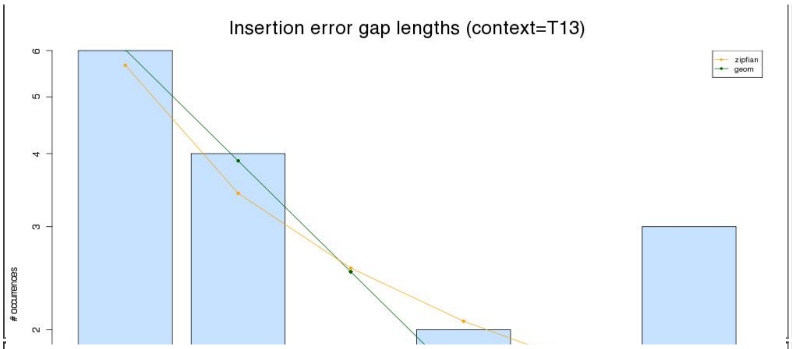	X^2 = 2.77169644219901 p-value = 0.596728220237383	0.645833333333333 X^2 = 8.98360110407515 p-value = 0.0615106849103599
A14	6	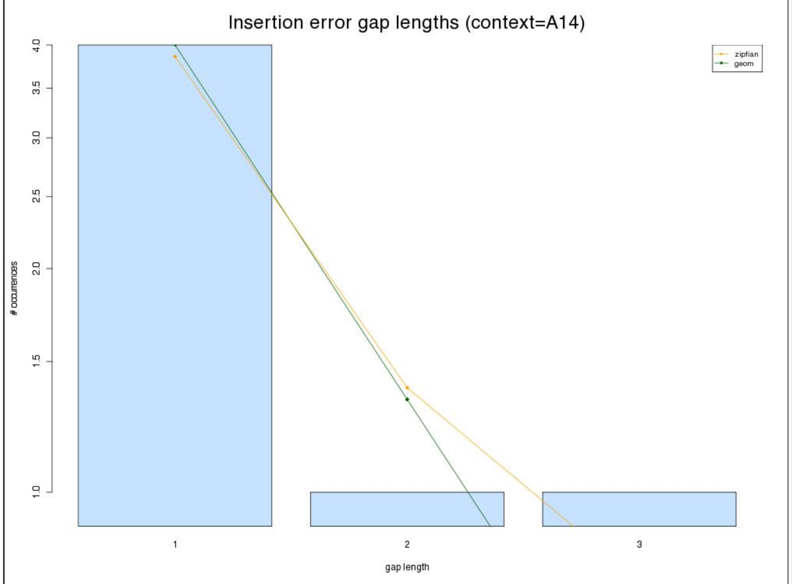	s = 1.48067490131275 X^2 = 0.187932256182633 p-value = 0.664643183539403	beta = 0.333333333333333 X^2 = 0.777777777777777 p-value = 0.377821637100064
T14	19	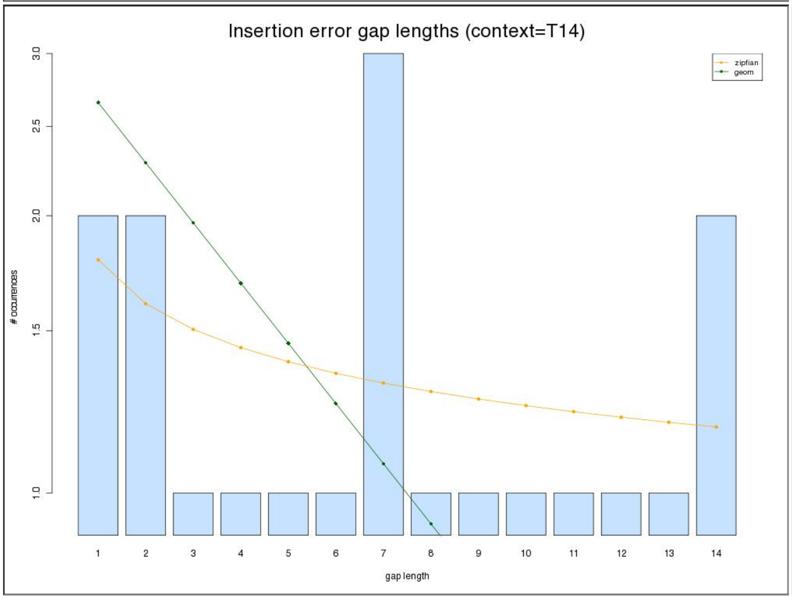	s = 0.158340782125942 X^2 = 3.62440875567043 p-value = 0.98930084112182	beta = 0.860294117647059 X^2 = 13.3133502327496 p-value = 0.346682418843568

A15	8	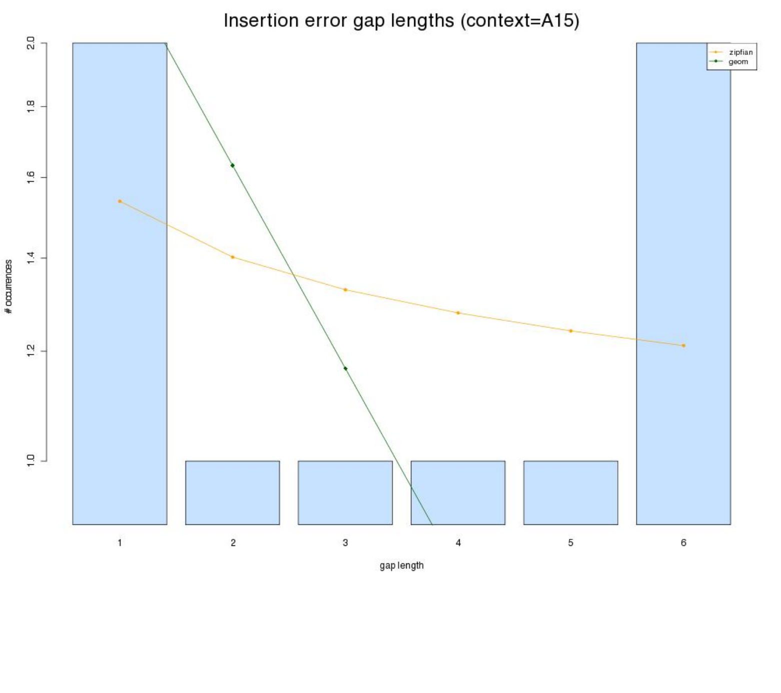	s = 0.133607967762624<br>X^2 =<br>0.956544519940105<br>p-value =<br>0.916312105293699	beta =<br>0.714285714285714<br>X^2 =<br>6.45063753096074<br>p-value =<br>0.167927311967506
T15	13	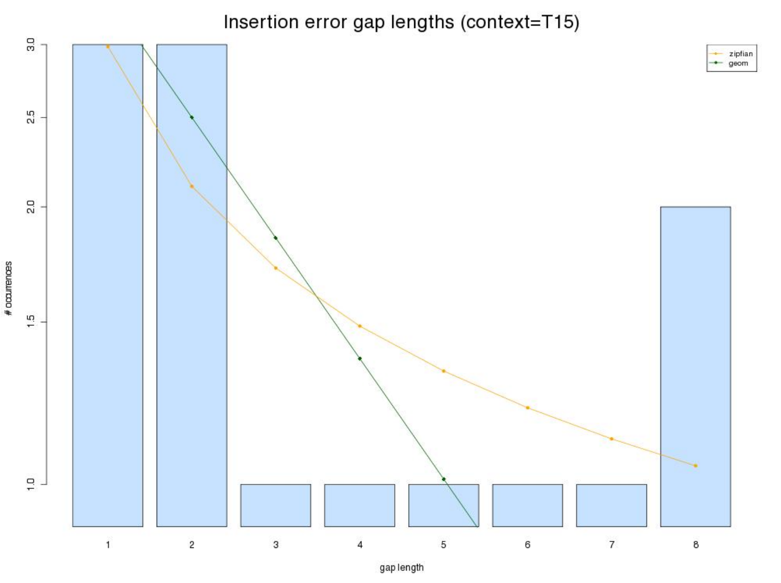	s = 0.503487237546934<br>X^2 =<br>1.83378665329945<br>p-value =<br>0.93433281140829	beta = 0.74<br>X^2 =<br>7.22325554211319<br>p-value =<br>0.30069337583502
A16	7	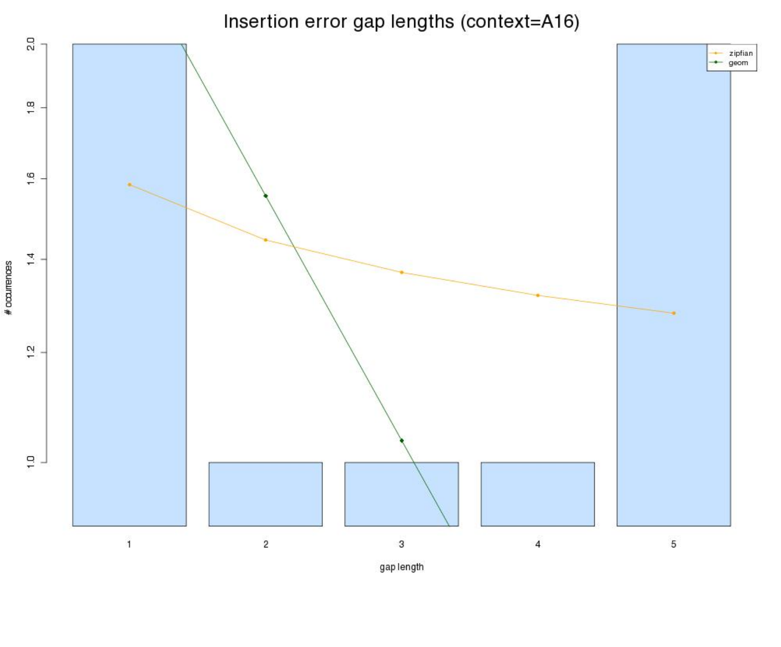	s = 0.132318446655207<br>X^2 =<br>0.827590945557859<br>p-value =<br>0.842856893927945	beta =<br>0.666666666666667<br>X^2 =<br>5.52461787184009<br>p-value =<br>0.137173588026781

T16	2	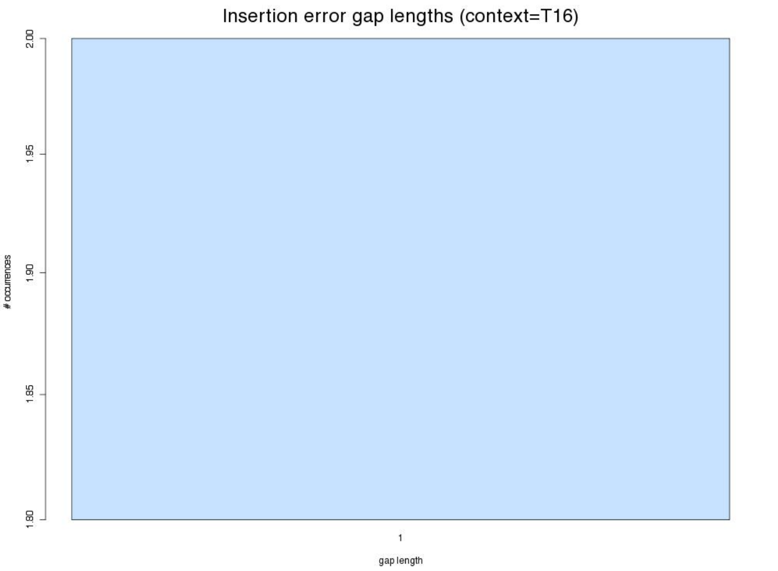	INVALID/ERROR	INVALID/ERROR
A17	1	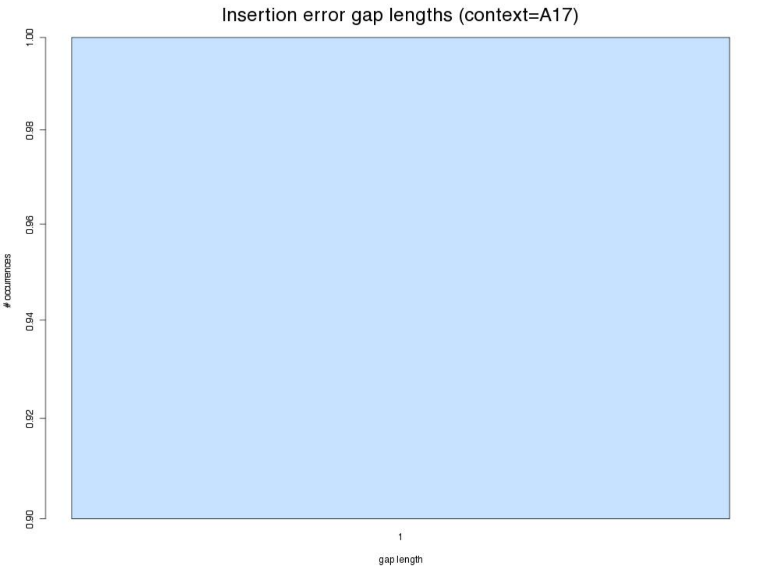	INVALID/ERROR	INVALID/ERROR

T17	5	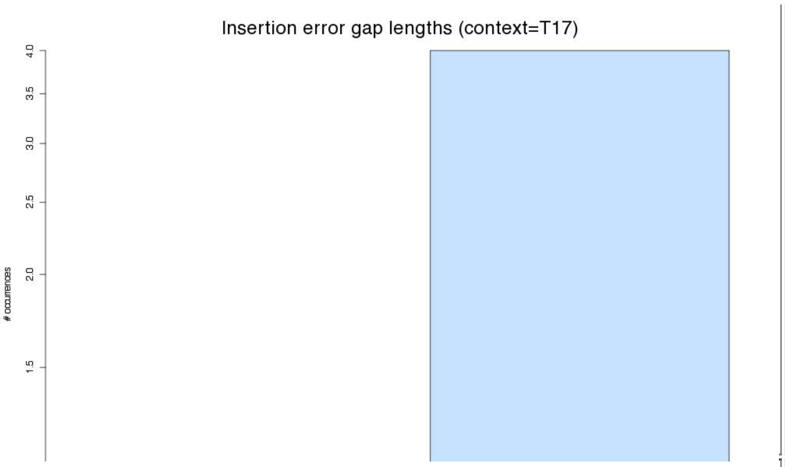	INVALID/ERROR	INVALID/ERROR
T18	4	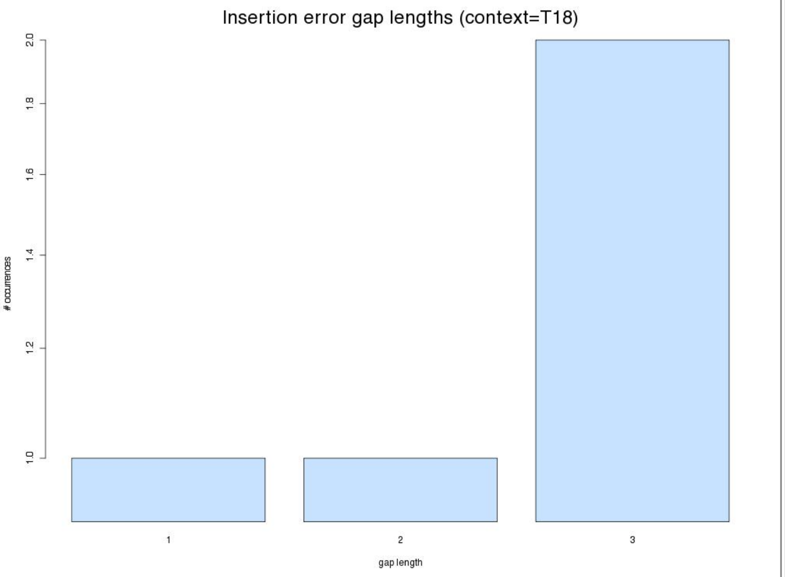	INVALID/ERROR	INVALID/ERROR
T19	5		s = 0.0926542887493549<br>X^2 = 0.395050902693468<br>p-value = 0.52965630969129	beta = 0.5<br>X^2 = 3.175<br>p-value = 0.0747732118999497

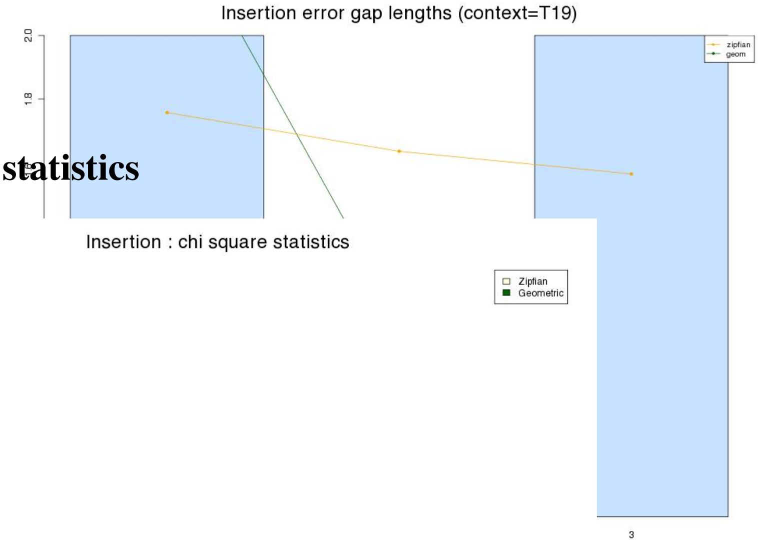

Chi square statistics

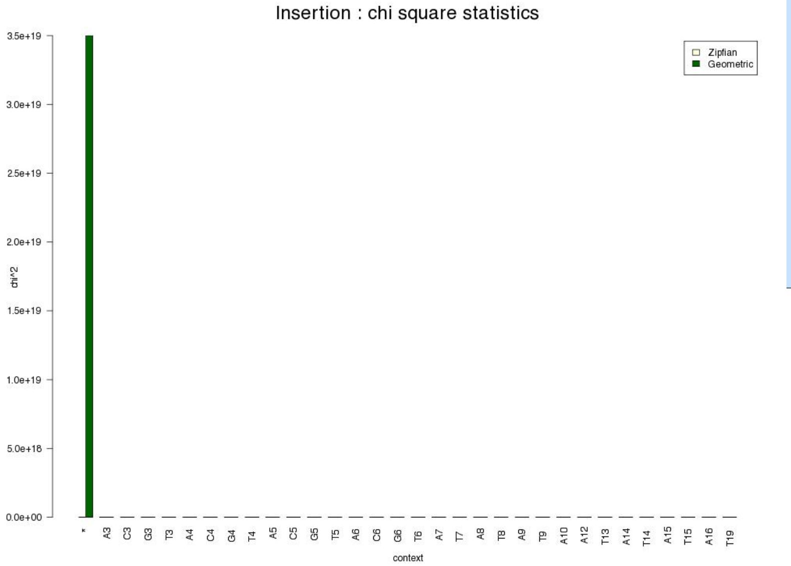

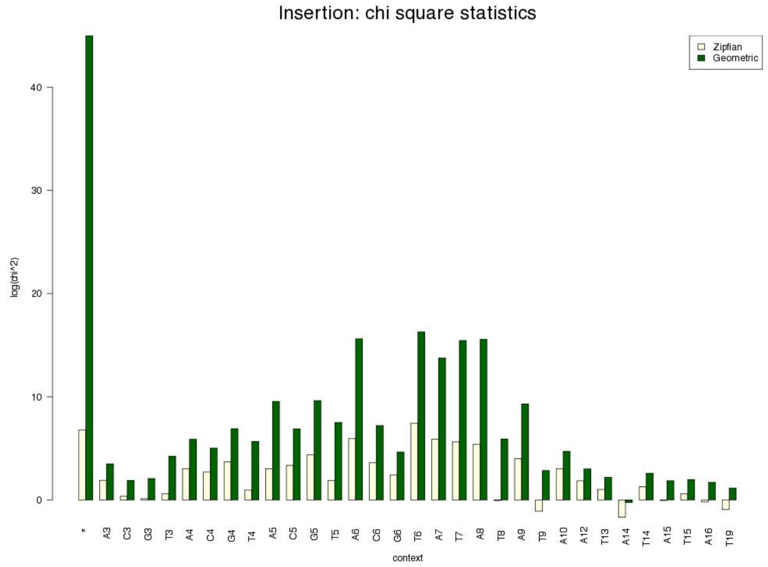

Average Pearson's residuals

Insertion: Average Pearson residuals

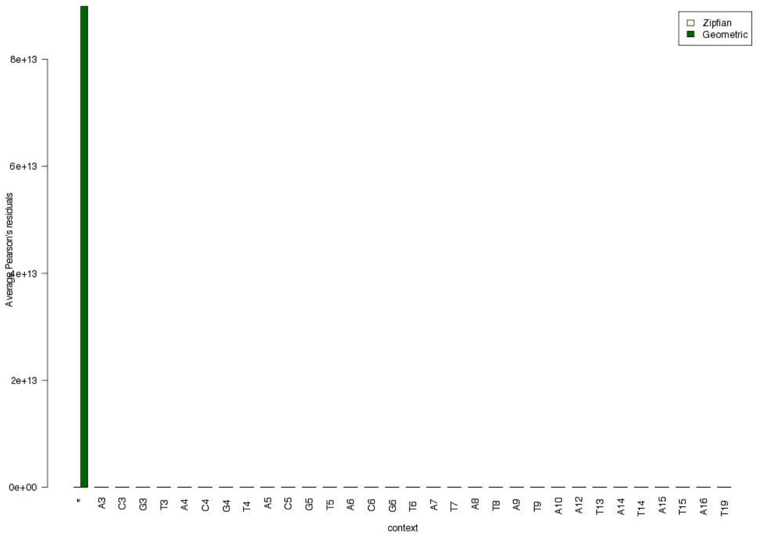

Insertion : Average Pearson residuals

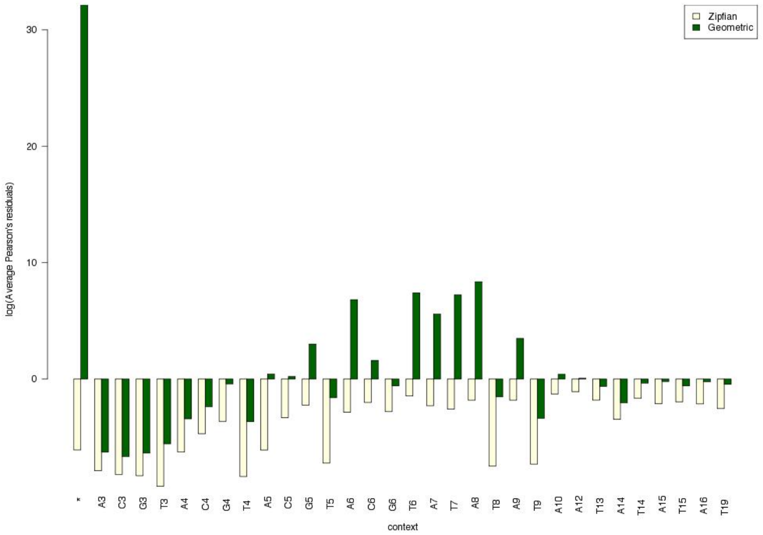

Insertion: Difference between average Pearson residuals

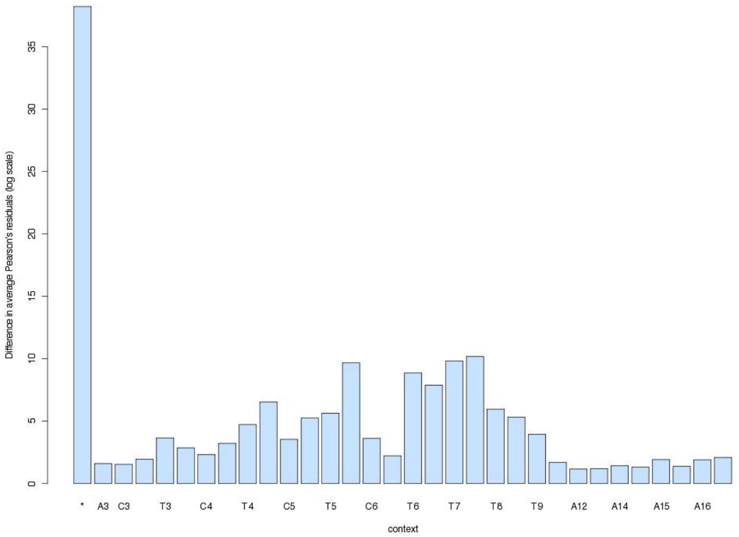

Mann U Tests

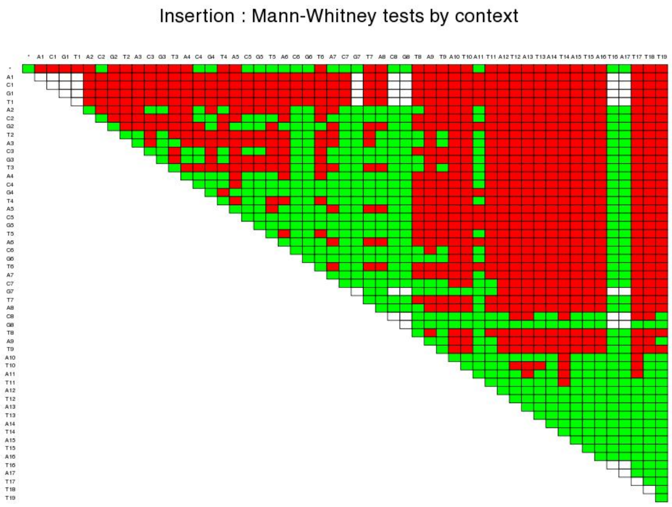

Mann U Tests by context length

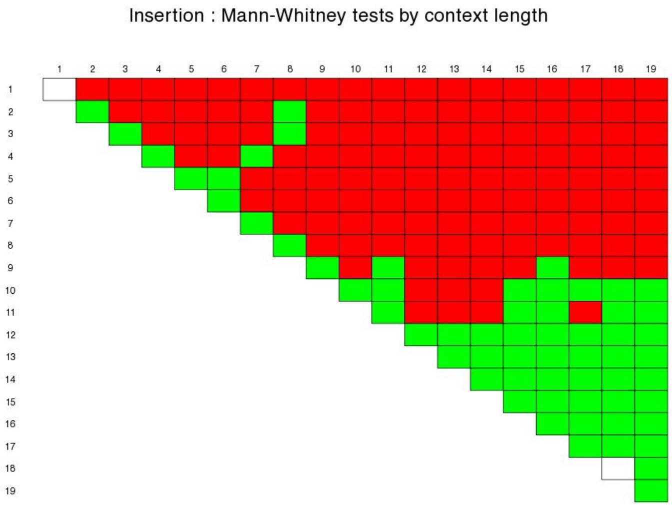

newbler Deletion

Deletion per context

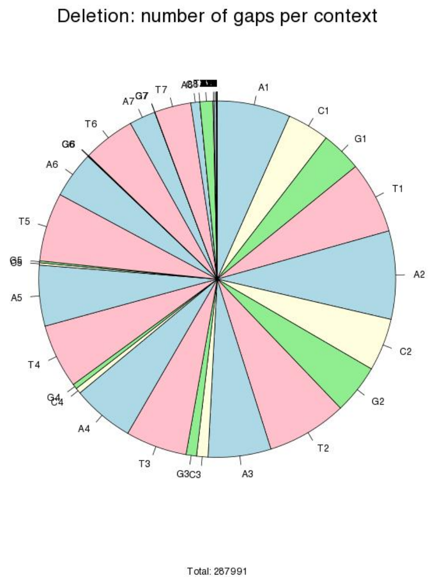

Deletion per context

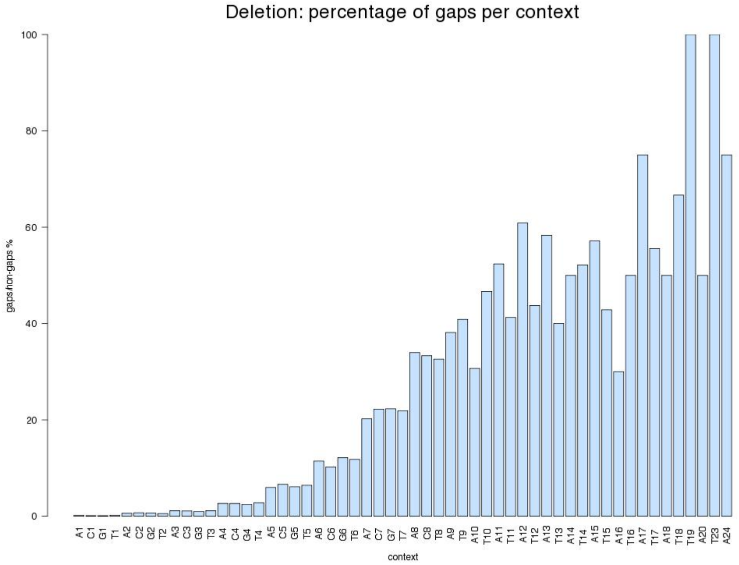

Context	#Data	Gap lengths	Zipfian fit	Geom fit
*	287991	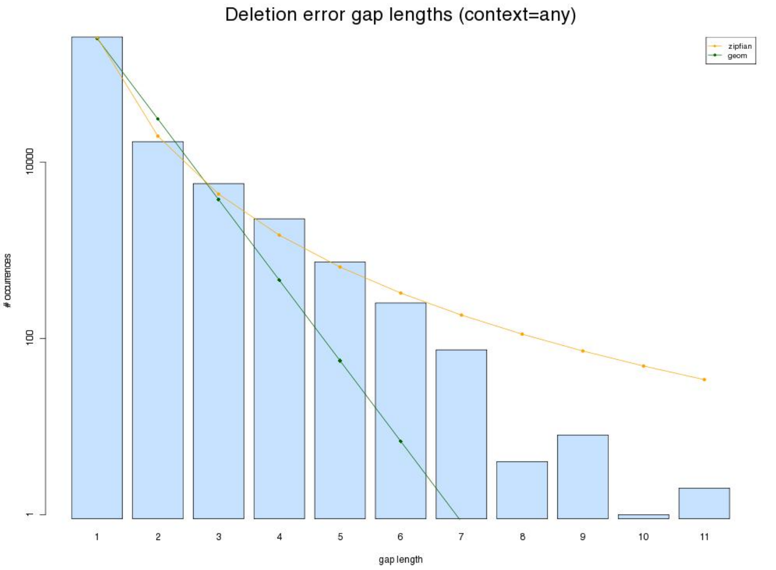	s = 3.7303818124779 X^2 = 1525.89089781184 p-value = 4.94065645841247e-323	beta = 0.121906376439524 X^2 = 65921.9611596078 p-value = 0

A1	19138	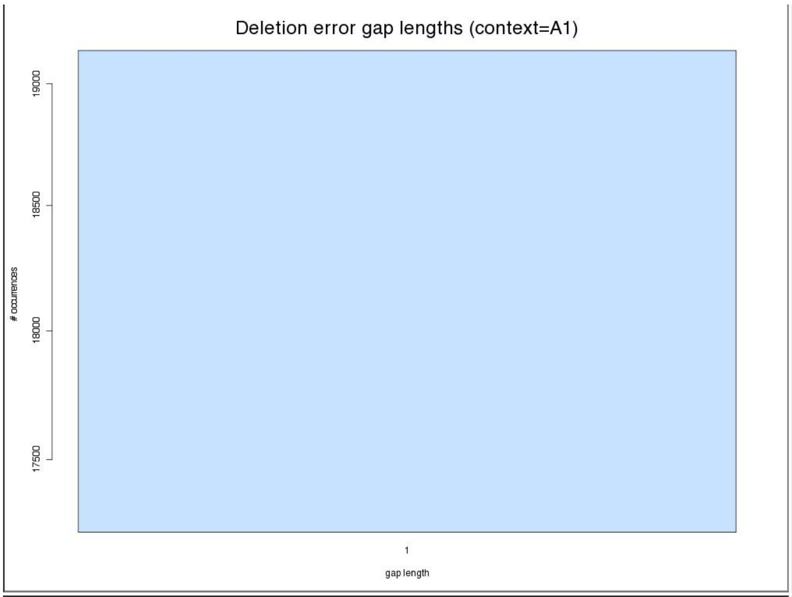	INVALID/ERROR	INVALID/ERROR
C1	10940	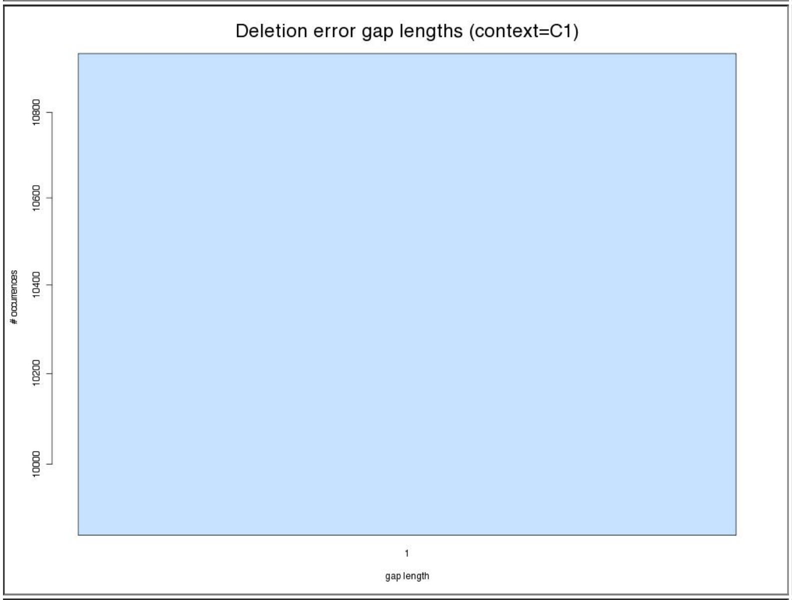	INVALID/ERROR	INVALID/ERROR
G1	10591	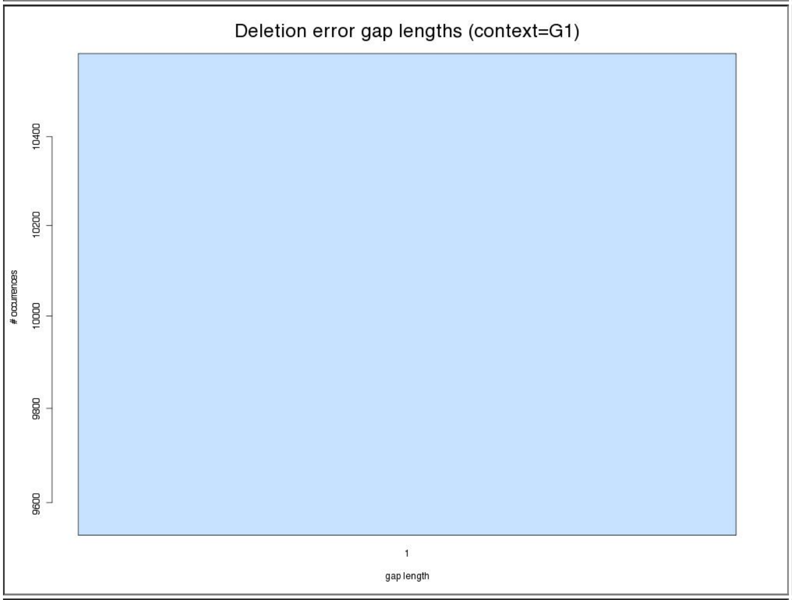	INVALID/ERROR	INVALID/ERROR

T1	18759	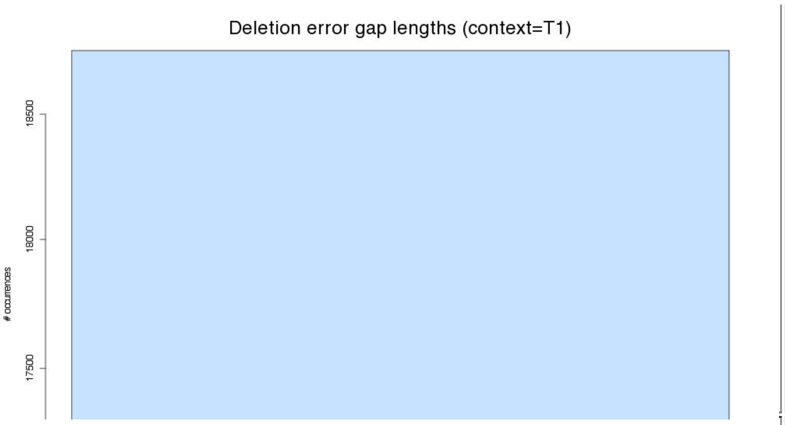	INVALID/ERROR	INVALID/ERROR
A2	23020	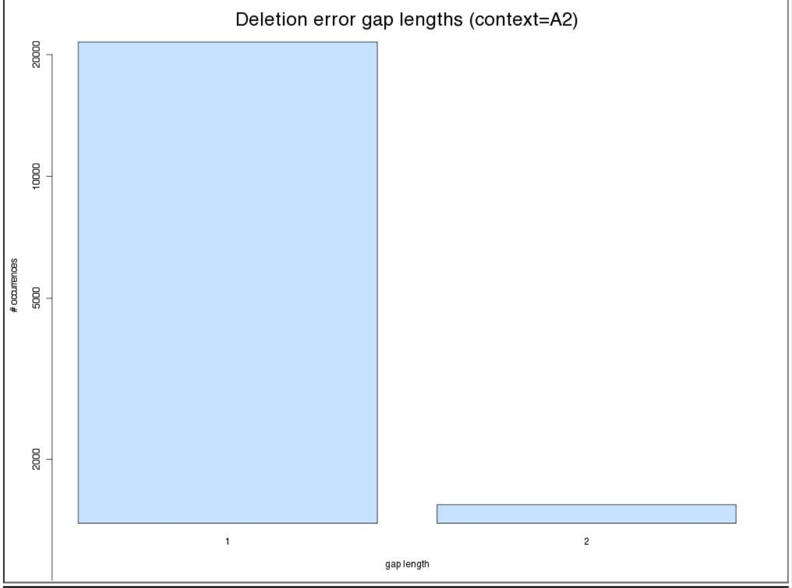	INVALID/ERROR	INVALID/ERROR
C2	13752	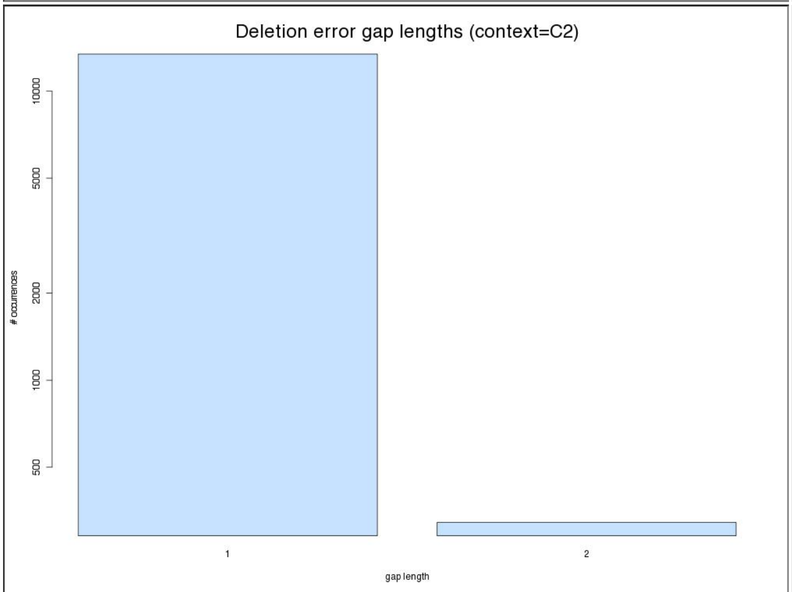	INVALID/ERROR	INVALID/ERROR

G2	12874	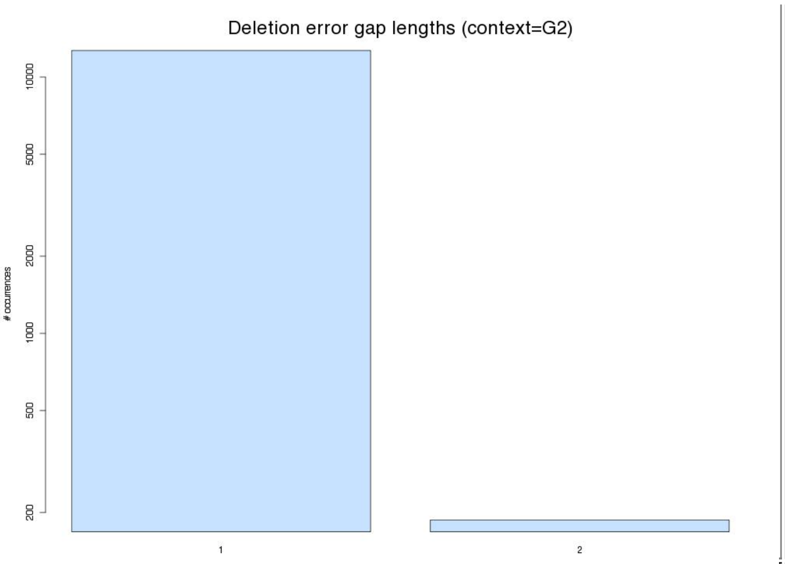	INVALID/ERROR	INVALID/ERROR
T2	20908	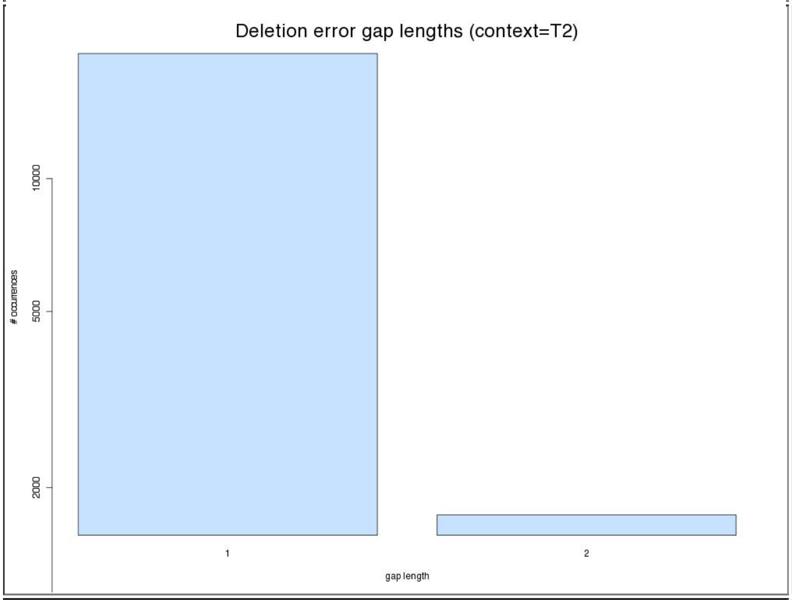	INVALID/ERROR	INVALID/ERROR
A3	16382	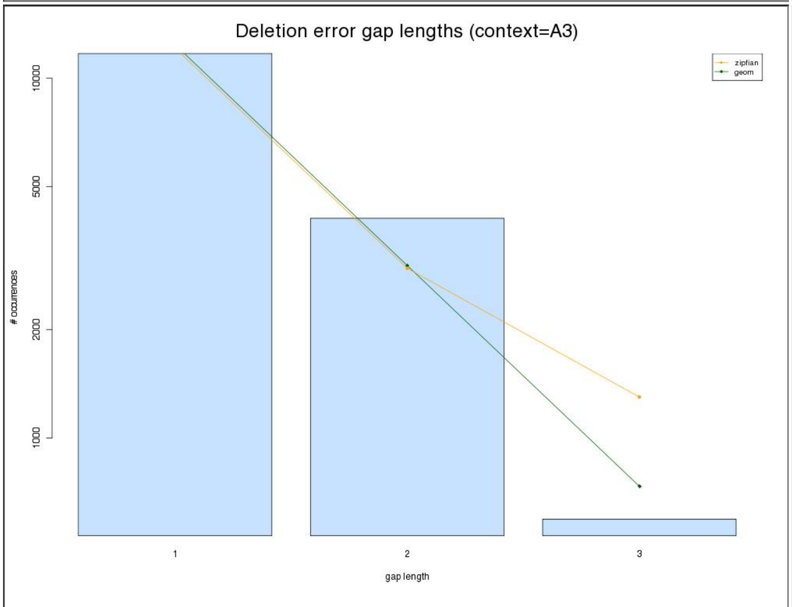	s = 2.03189175918716<br>X^2 = 817.976271148564<br>p-value = 6.66422943205951e-180	beta = 0.243430471528195<br>X^2 = 439.83414022268<br>p-value = 1.17636938323754e-97

C3	2976	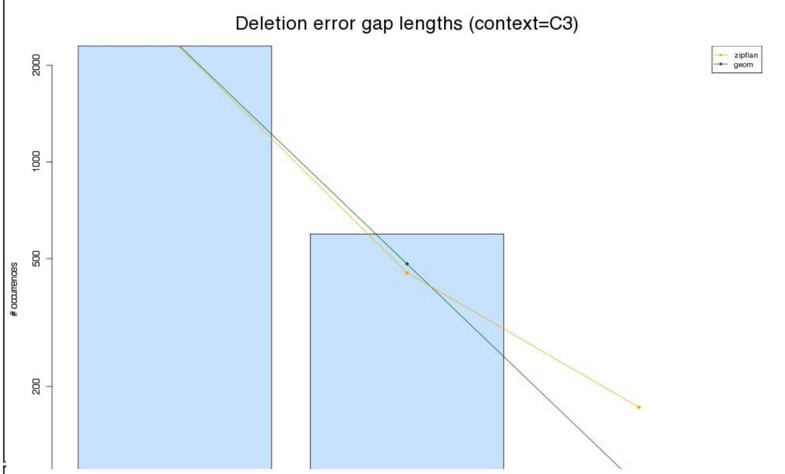	s = 2.37858007008878<br>X^2 =<br>96.0306422172164<br>p-value =<br>1.13119058398999e-22	beta =<br>0.203212851405623<br>X^2 =<br>32.6954845870646<br>p-value =<br>1.07787328561388e-08
G3	2722	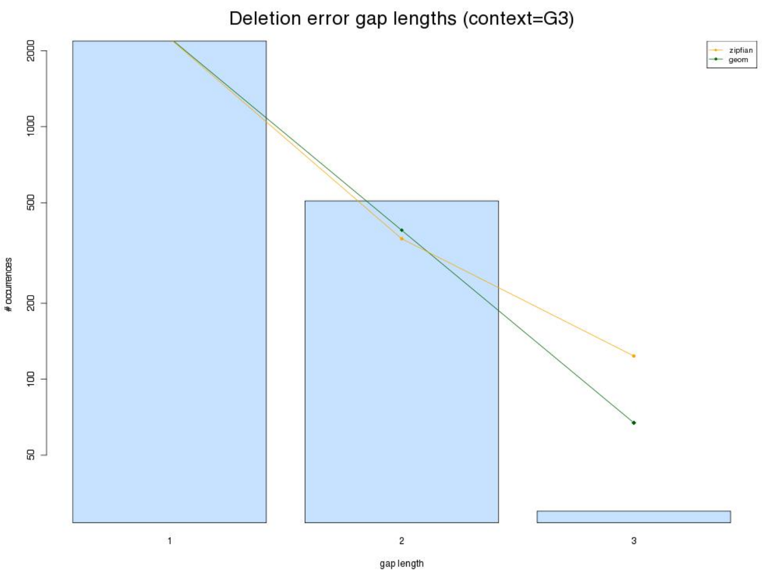	s = 2.63735018691134<br>X^2 =<br>133.154604880583<br>p-value =<br>8.36381461255204e-31	beta =<br>0.172644376899696<br>X^2 =<br>59.1306357588239<br>p-value =<br>1.47544170844702e-14
T3	16011	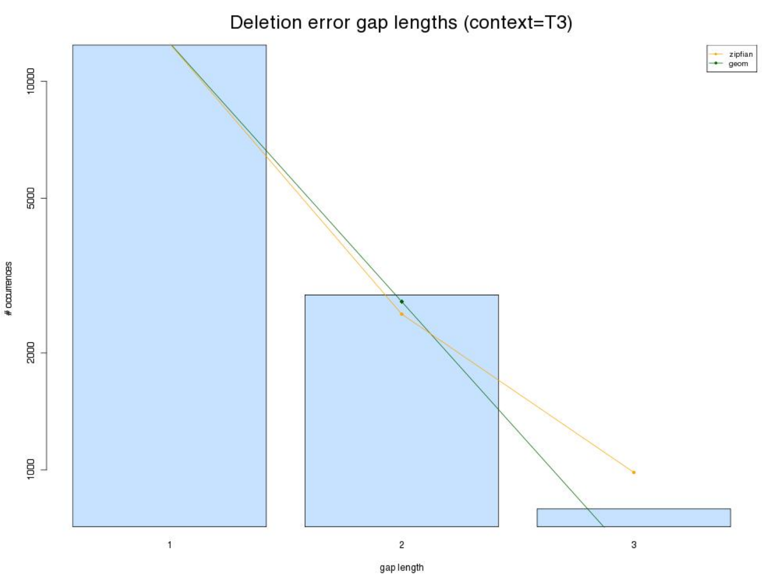	s = 2.31285133884244<br>X^2 = 74.84817906128<br>p-value =<br>5.0833925638005e-18	beta =<br>0.215915768854065<br>X^2 =<br>80.9326996652077<br>p-value =<br>2.33537137181142e-19
			s = 2.56270880377719<br>X^2 =<br>881.606024178574	beta =<br>0.237194604282615

A4	16173	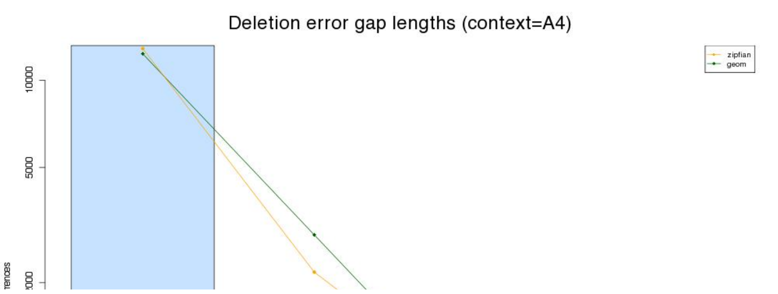	p-value = 3.64488846510436e-192	X^2 = 1818.25405369029<br>p-value = 0
C4	1477	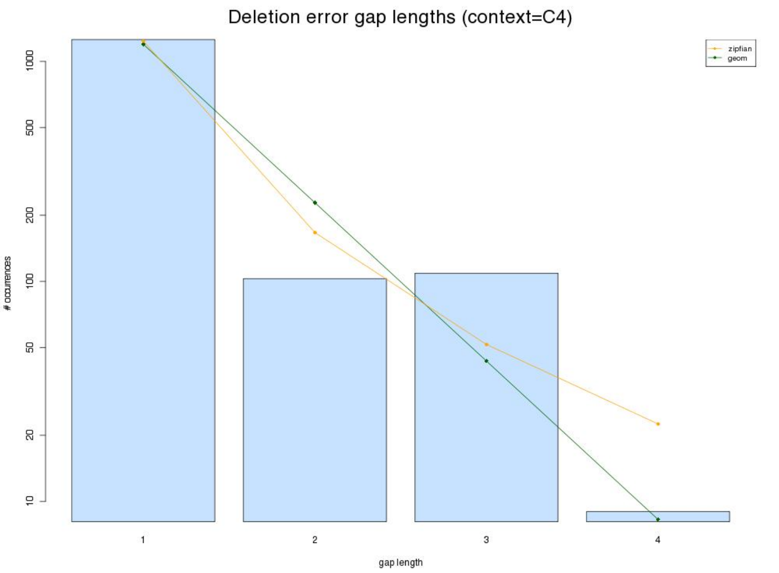	s = 2.88939322208711<br>X^2 = 96.3777553423416<br>p-value = 1.17987592153131e-21	beta = 0.190684931506849<br>X^2 = 170.434040899389<br>p-value = 9.78854754134094e-38
G4	1283	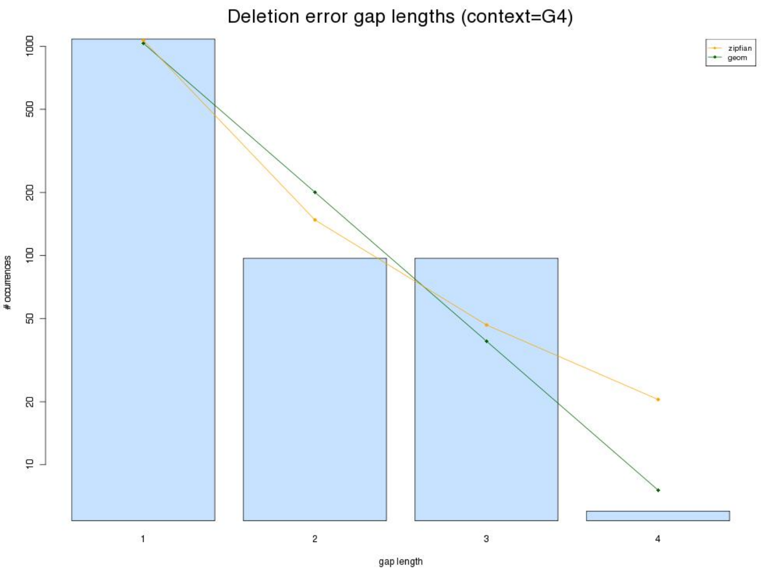	s = 2.85162448699112<br>X^2 = 82.6663128330726<br>p-value = 1.12005216191009e-18	beta = 0.194095477386935<br>X^2 = 142.720211396448<br>p-value = 1.02023417531867e-31

T4	16759	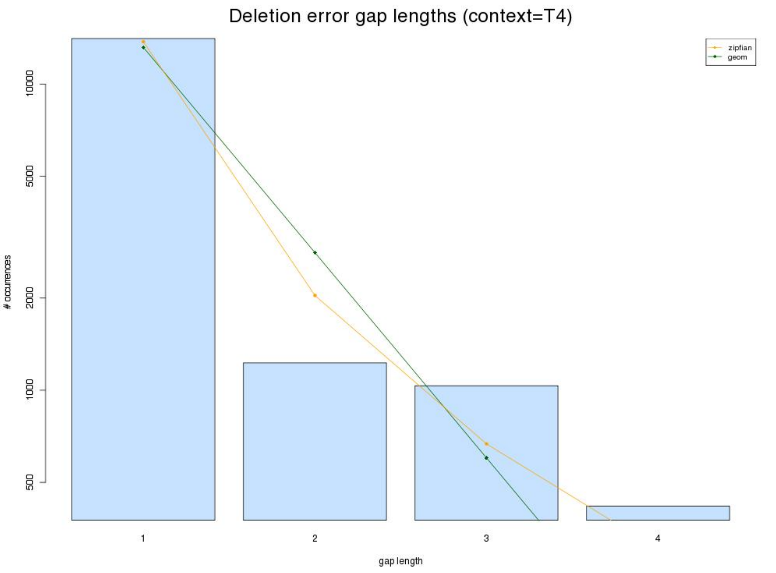	s = 2.75317464623204<br>X^2 =<br>575.549475856226<br>p-value =<br>1.04958903901607e-<br>125	beta =<br>0.213524801726970<br>X^2 =<br>1921.50787640793<br>p-value = 0
A5	15857	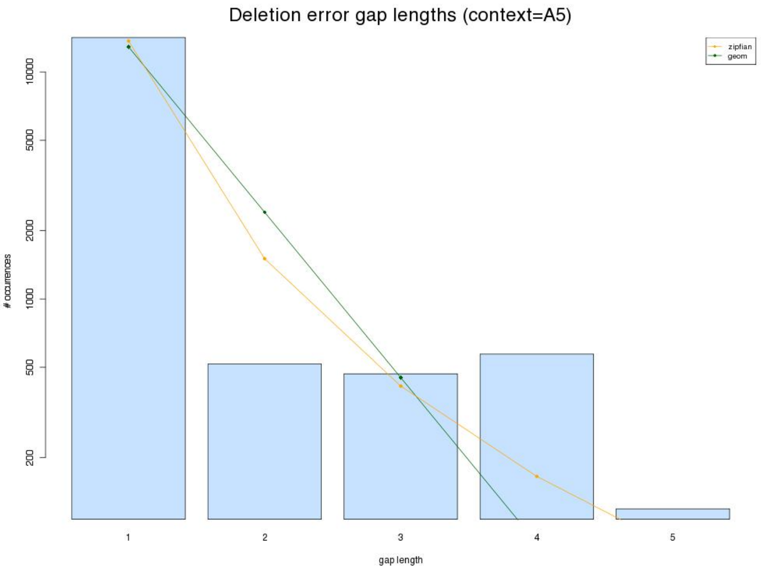	s = 3.18594408676657<br>X^2 =<br>1690.76087031724<br>p-value = 0	beta =<br>0.186903907291560<br>X^2 =<br>5120.60527690562<br>p-value = 0
C5	602		s = 3.22114903235939<br>X^2 =<br>33.9044961109349<br>p-value =<br>4.34242387382752e-08	beta =<br>0.162726008344923<br>X^2 =<br>123.301006163010<br>p-value =<br>1.68084127891389e-<br>27

		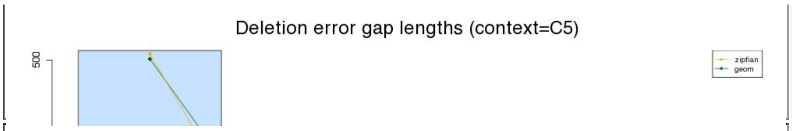		
G5	507	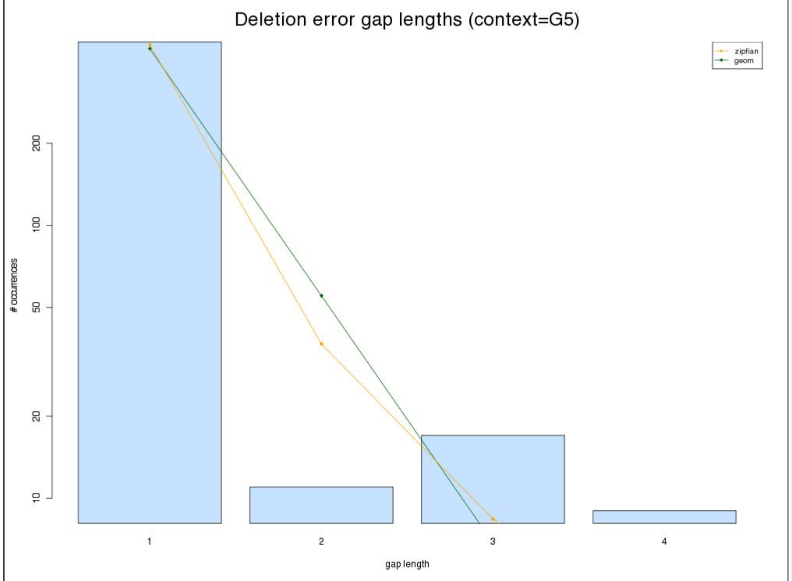	s = 3.64224010188977 X^2 = 39.6002660625332 p-value = 2.51716383567049e-09	beta = 0.124352331606218 X^2 = 129.624900788309 p-value = 7.11722073259331e-29
T5	17910	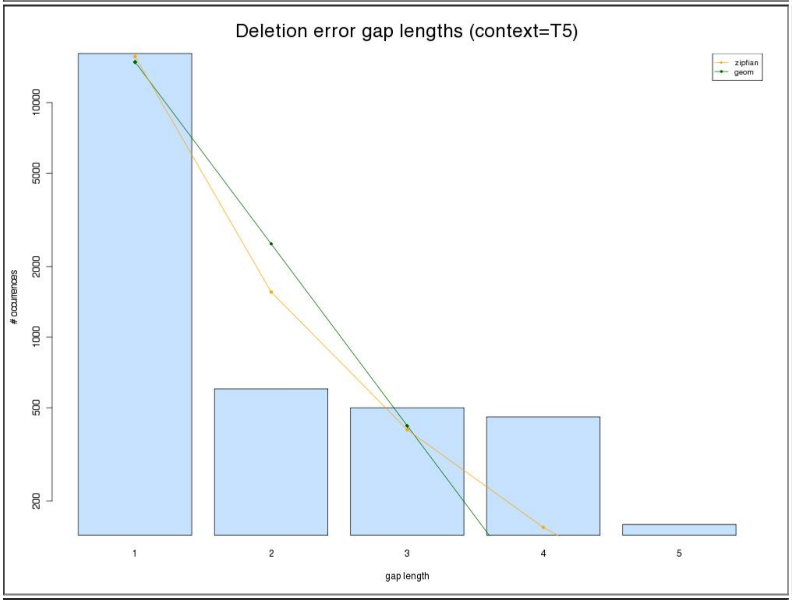	s = 3.33412681446039 X^2 = 1316.02203584982 p-value = 4.91300443257319e-285	beta = 0.167712254286909 X^2 = 5531.27562958112 p-value = 0

A6	12208	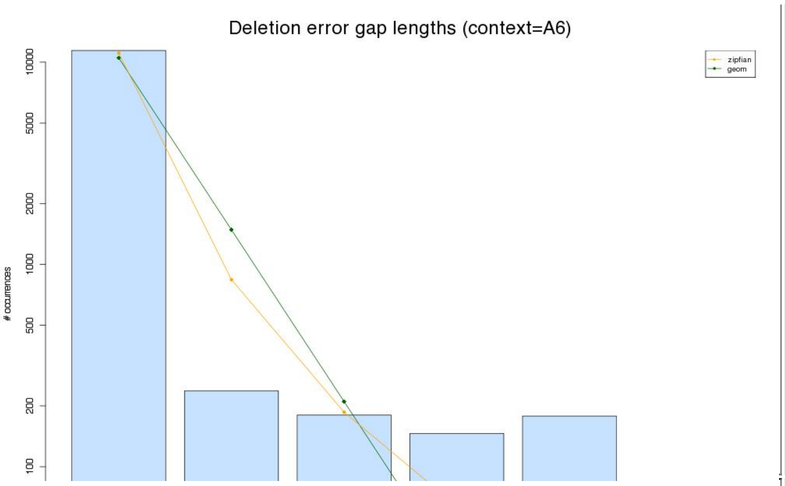	s = 3.71940721430899 X^2 = 1466.98118513617 p-value = 2.06571000652441e-316	beta = 0.141490857946554 X^2 = 13401.8102381151 p-value = 0
C6	141	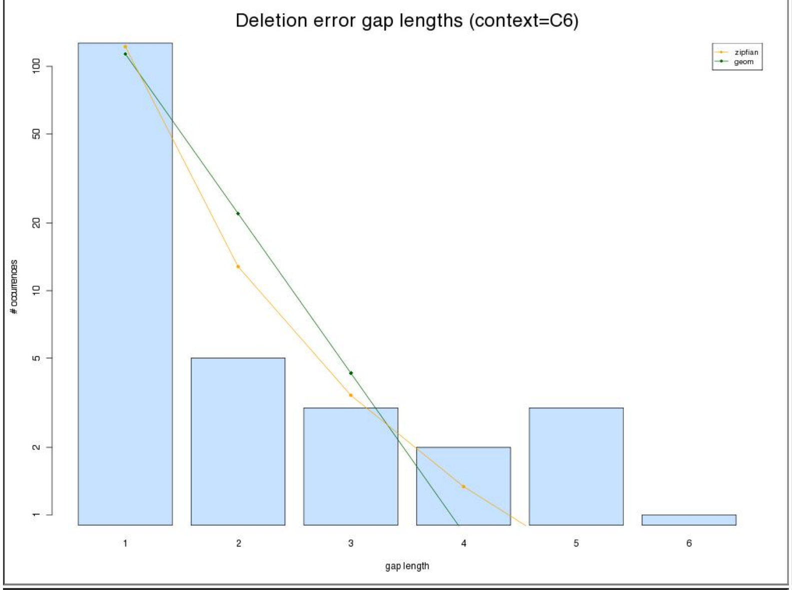	s = 3.25837579750473 X^2 = 15.0286023977881 p-value = 0.00464226003314742	beta = 0.194285714285714 X^2 = 96.3965818339653 p-value = 5.75040236184136e-20
G6	147	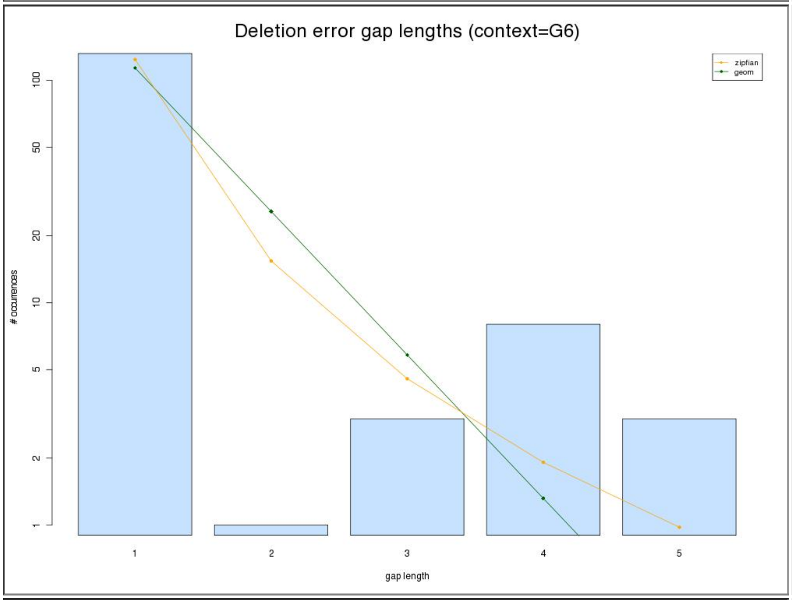	s = 3.00975917382199 X^2 = 38.0424107624465 p-value = 2.76863772356009e-08	beta = 0.226315789473684 X^2 = 86.4104282104048 p-value = 1.29234522536602e-18
			s = 3.68526340601055 X^2 = 1326.62932782919	beta = 0.143838205645161

T6	13589	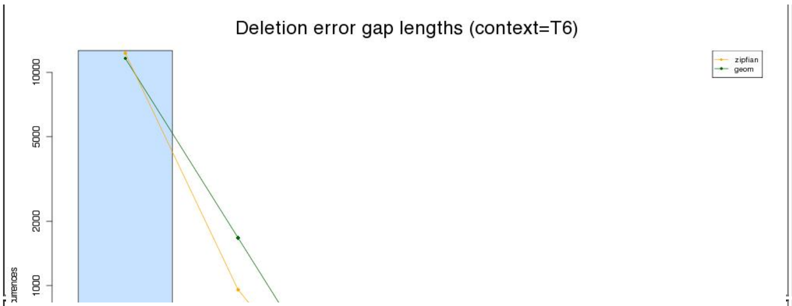	p-value = 5.60370849810637e-286	X^2 = 15006.5288421020 p-value = 0
A7	6845	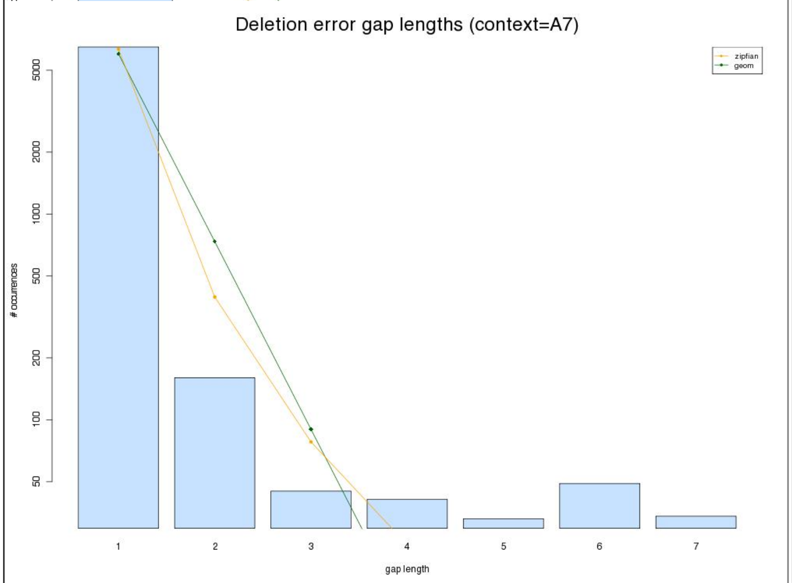	s = 4.00062180678743 X^2 = 993.184452956652 p-value = 1.79643340644771e-212	beta = 0.12232337479164 X^2 = 73200.6856035933 p-value = 0
C7	50	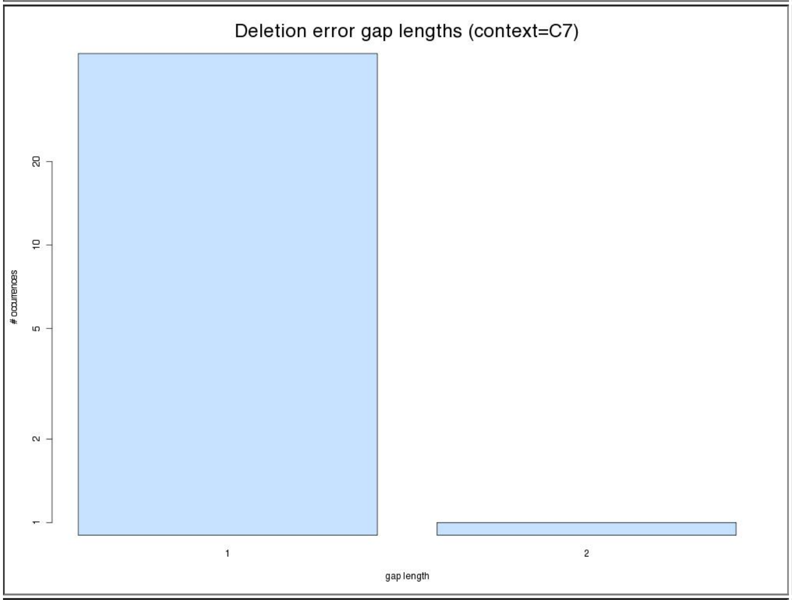	INVALID/ERROR	INVALID/ERROR
			s = 2.70175083232825	beta =

G7	33	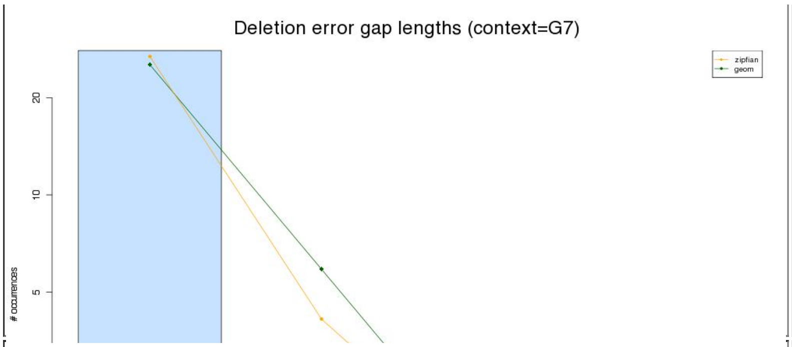	X^2 = 4.18906574896551 p-value = 0.123127746334631	0.232558139534884 X^2 = 11.8271425166488 p-value = 0.00270251824227842
T7	9512	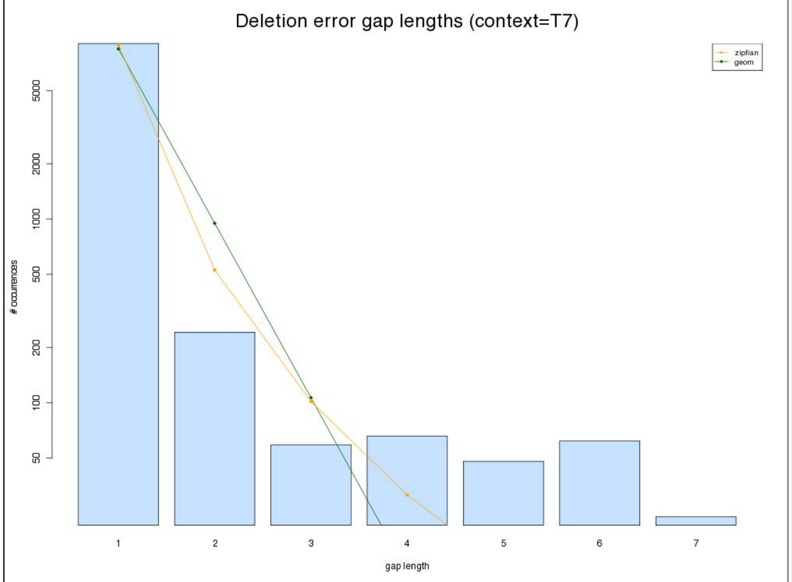	s = 4.06371799446981 X^2 = 958.696932683944 p-value = 5.25176491050475e-205	beta = 0.112355356476297 X^2 = 61614.5294143215 p-value = 0
A8	2359	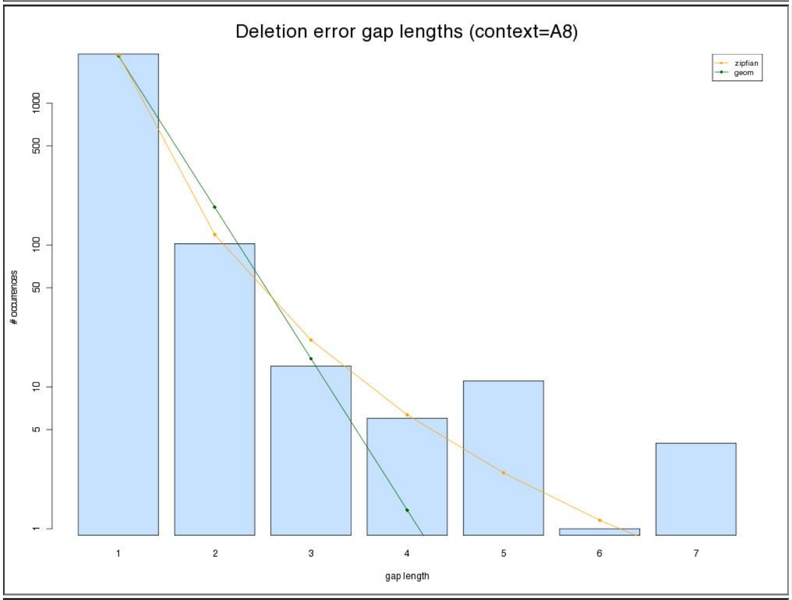	s = 4.22020049664641 X^2 = 53.552910330594 p-value = 2.58953003815933e-10	beta = 0.0856589147286821 X^2 = 19943.6924692809 p-value = 0

C8	3	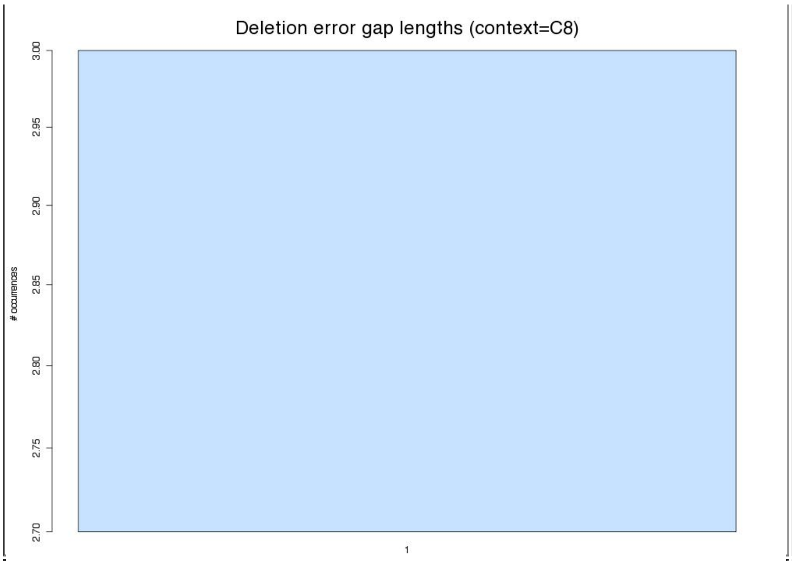	INVALID/ERROR	INVALID/ERROR
T8	3368	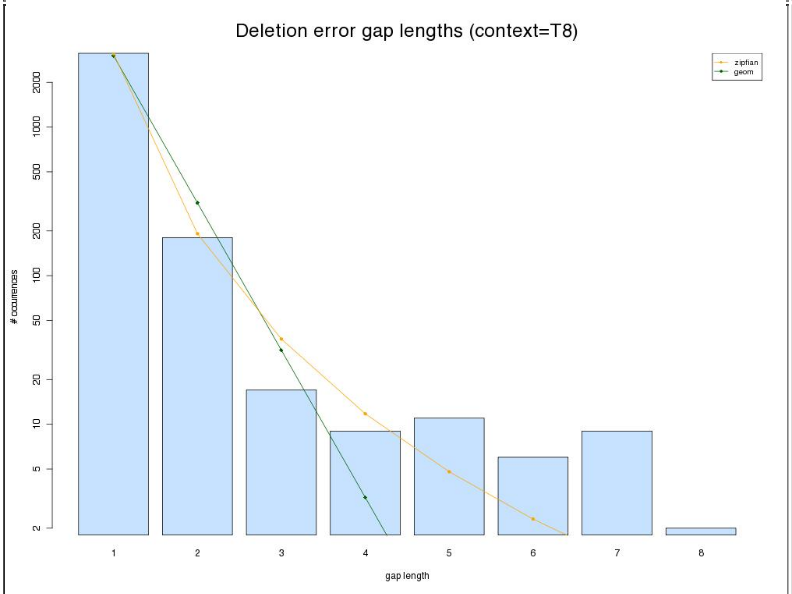	s = 4.02454690142885<br>X^2 =<br>77.4980669818713<br>p-value =<br>1.17329308708217e-14	beta =<br>0.102106105038656<br>X^2 =<br>36527.5187618464<br>p-value = 0
A9	316	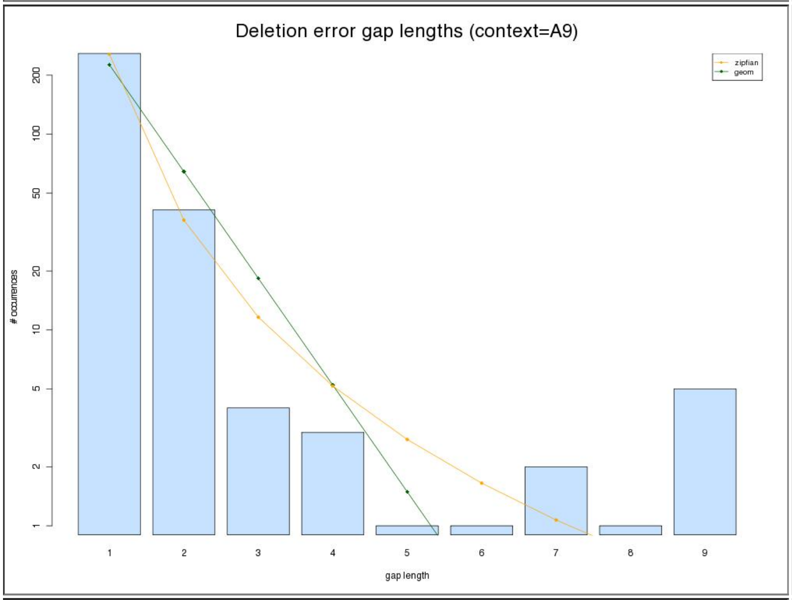	s = 2.81494311138736<br>X^2 =<br>46.7120990855964<br>p-value =<br>6.352231534372e-08	beta =<br>0.285067873303167<br>X^2 =<br>2609.75024885114<br>p-value = 0

T9	563	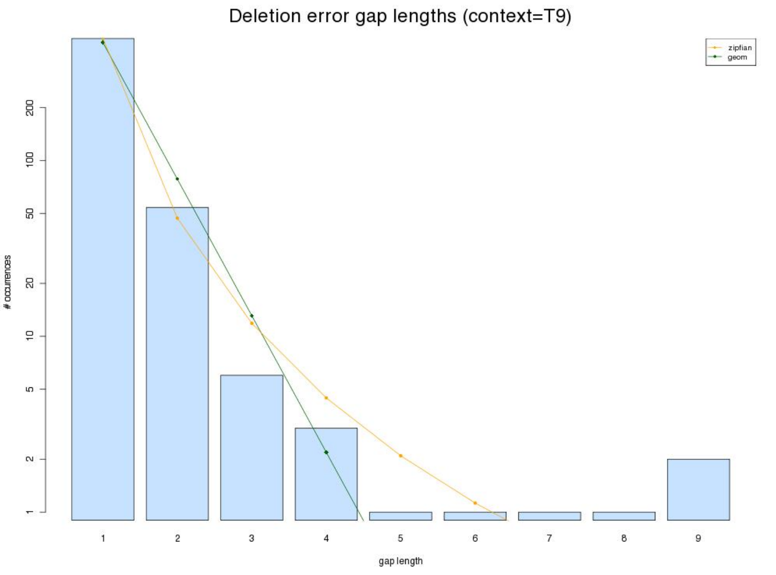	s = 3.3964052043 X^2 = 16.3078188800407 p-value = 0.0224481556328011	beta = 0.167159763313609 X^2 = 14696.9523797269 p-value = 0
A10	31	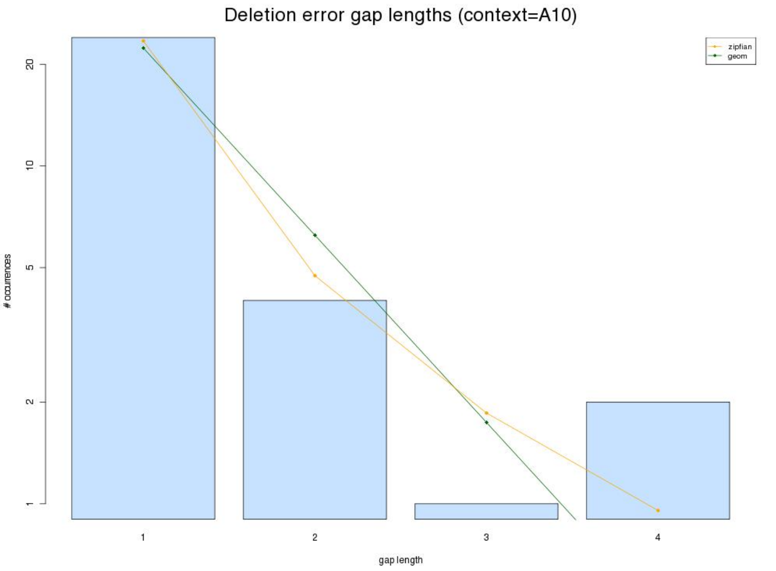	s = 2.30905454569390 X^2 = 1.66397177236885 p-value = 0.435184201332572	beta = 0.279069767441861 X^2 = 5.96010988258343 p-value = 0.0507900433176548
T10	87		s = 2.06543356561978 X^2 = 18.2537902642195 p-value = 0.0194022189545671	beta = 0.491228070175439 X^2 = 98.3144949910199 p-value = 9.43306790447387e-18

		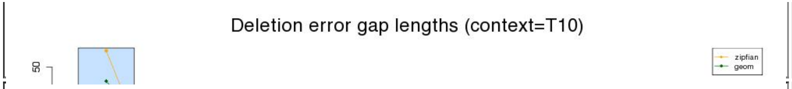		
A11	22	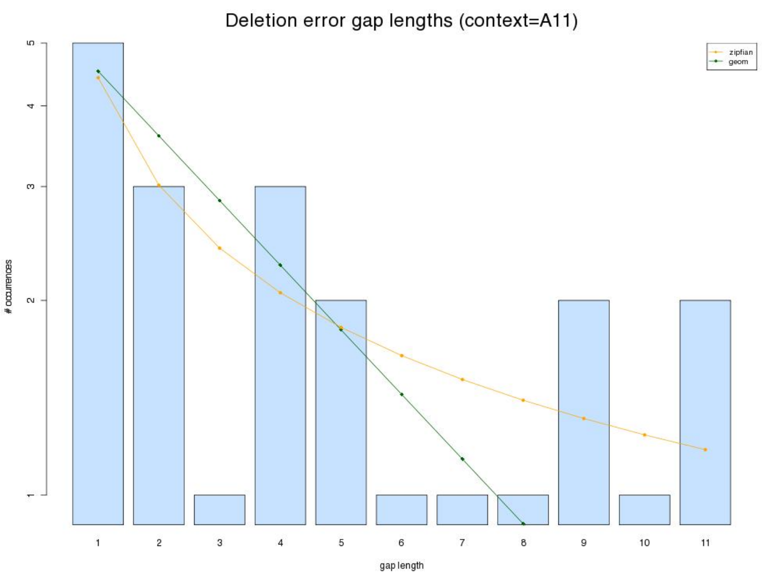	s = 0.552331016783963<br>X^2 =<br>2.87449044007541<br>p-value =<br>0.9690636100729	beta =<br>0.794392523364486<br>X^2 =<br>9.67462916531639<br>p-value =<br>0.377462119929868
T11	26	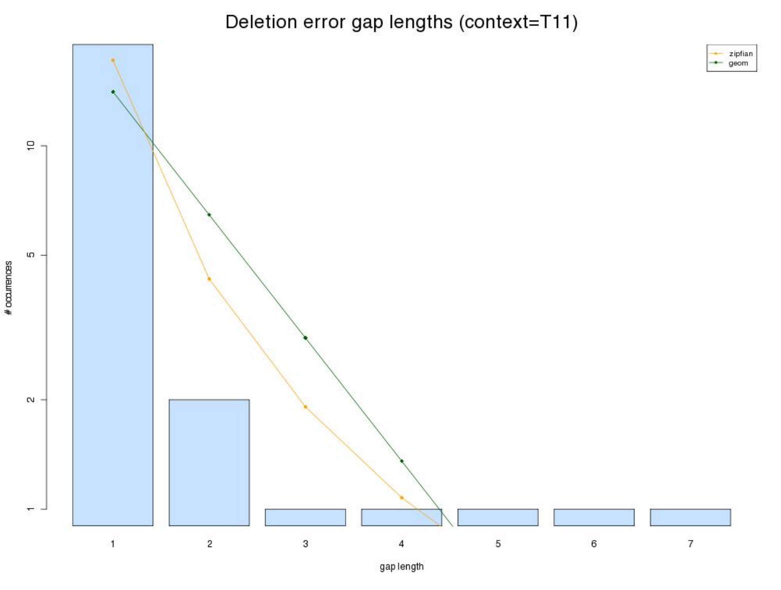	s = 1.99967253983709<br>X^2 =<br>3.76959805998939<br>p-value =<br>0.583041651292252	beta =<br>0.458333333333333<br>X^2 =<br>13.9971376515854<br>p-value =<br>0.0156276083406878
A12	14	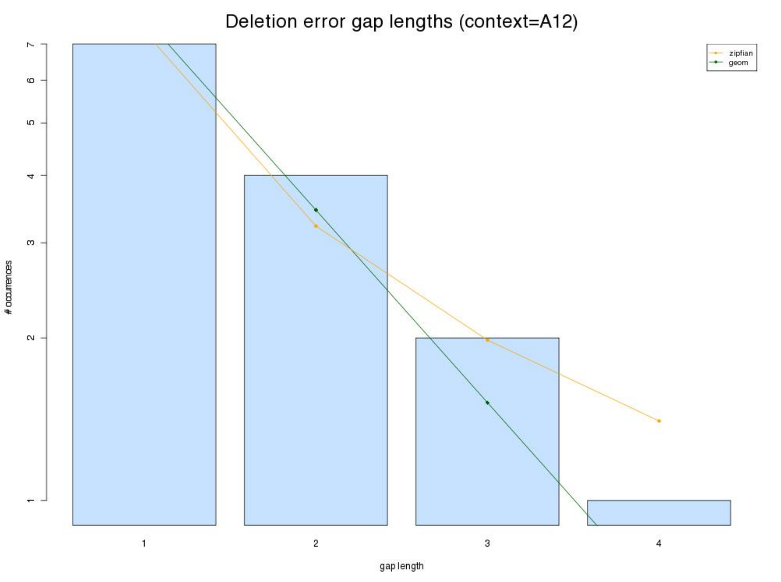	s = 1.19842587491795<br>X^2 =<br>0.325326281349461<br>p-value =<br>0.84987742933783	beta = 0.44<br>X^2 =<br>0.4961960437394<br>p-value =<br>0.780283454686194

T12	25	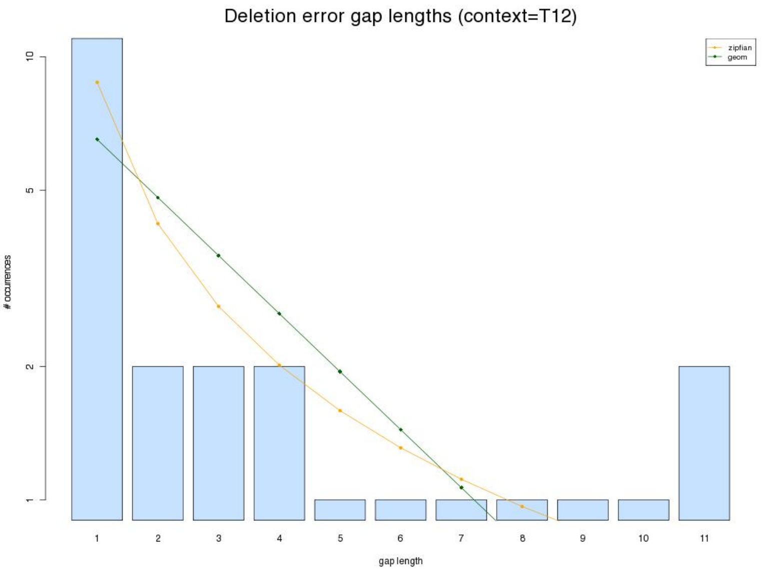	s = 1.06023118132422 X^2 = 4.81844157250669 p-value = 0.849839203471597	beta = 0.739583333333333 X^2 = 16.1523816623622 p-value = 0.0637668476621548
A13	20	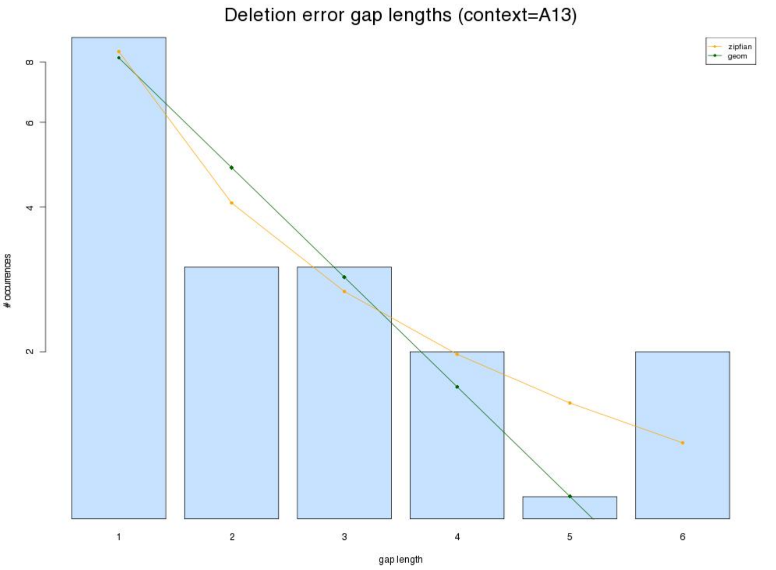	s = 1.04470658640519 X^2 = 0.956281509675145 p-value = 0.916351088336651	beta = 0.591836734693878 X^2 = 4.18374006134246 p-value = 0.381710071034104
T13	14		s = 1.30820790518368 X^2 = 0.825761971586434 p-value = 0.661741034725801	beta = 0.44 X^2 = 2.89580352008832 p-value = 0.235062989566924

		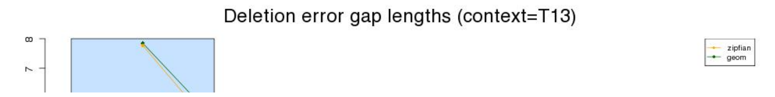		
A14	4	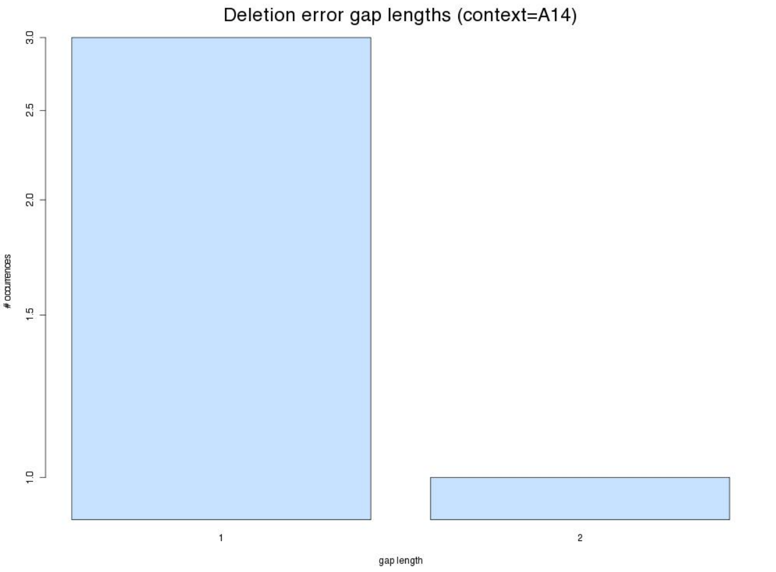	INVALID/ERROR	INVALID/ERROR
T14	12	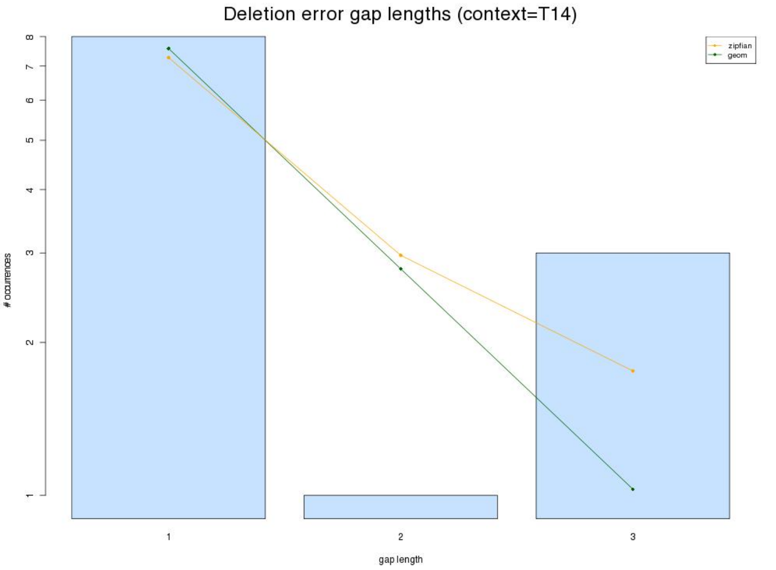	s = 1.29262575813854 X^2 = 2.25613272129751 p-value = 0.133086042964734	beta = 0.368421052631579 X^2 = 4.95121637856678 p-value = 0.0260723183721473
A15	11		s = 0.243359866092211 X^2 = 0.884776682504825 p-value = 0.97131395173215	beta = 0.73170731707317 X^2 = 5.99803332543462 p-value = 0.306410331443849

		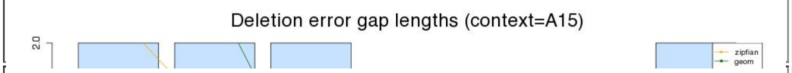		
T15	3	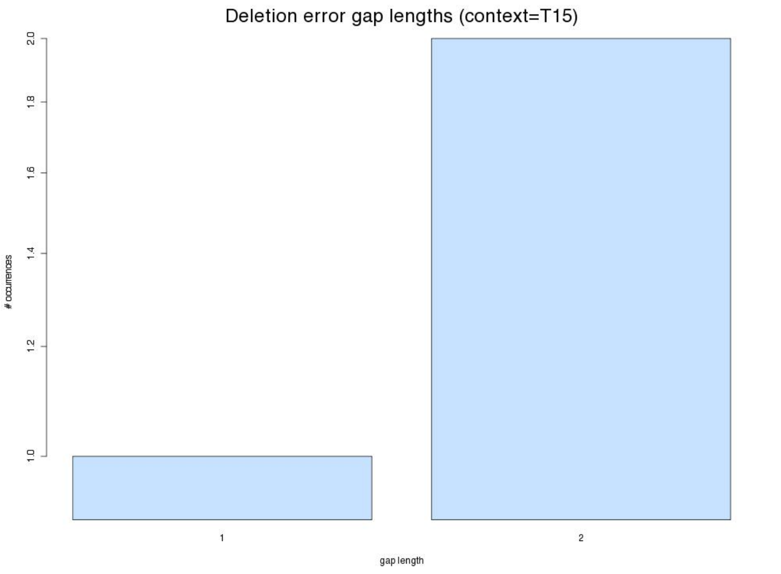	INVALID/ERROR	INVALID/ERROR
A16	12	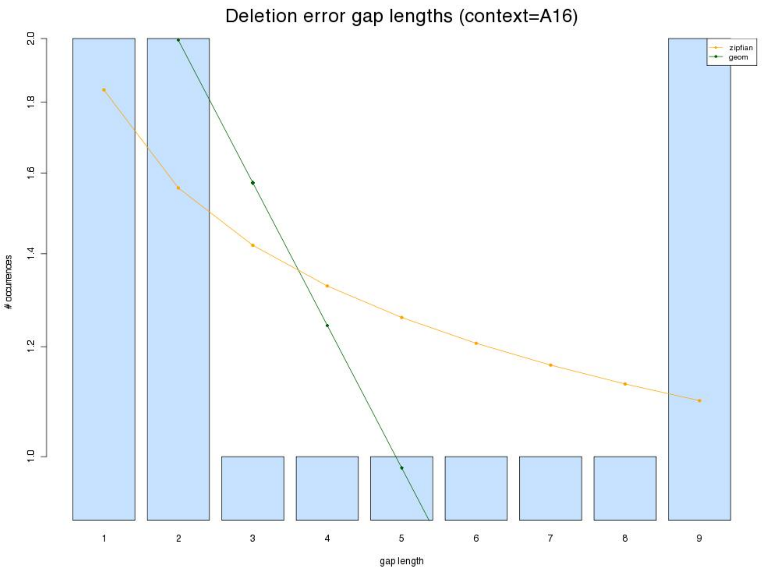	s = 0.234428984065603 X^2 = 1.21155639204441 p-value = 0.99065837569117	beta = 0.789473684210526 X^2 = 8.10652266288131 p-value = 0.323294687645877
T16	6	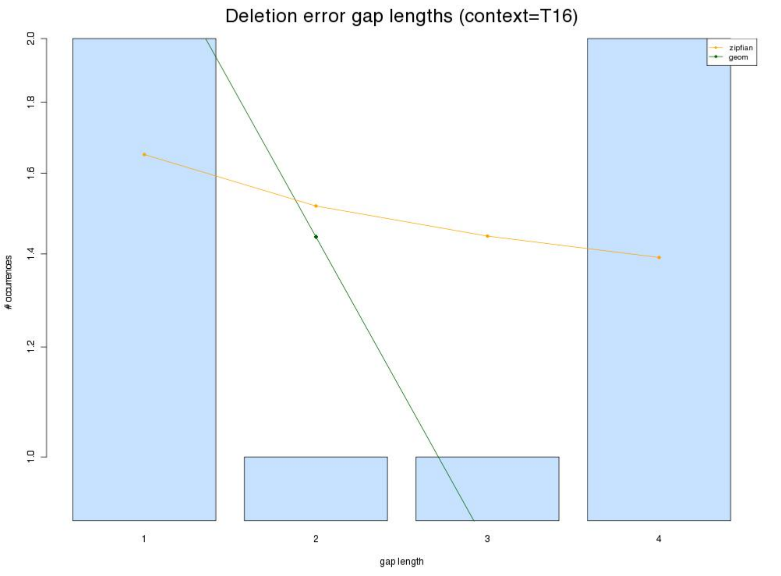	s = 0.122967886301763 X^2 = 0.650635373179079 p-value = 0.722297852847837	beta = 0.6 X^2 = 4.45696790123457 p-value = 0.107691572180377

A17	8	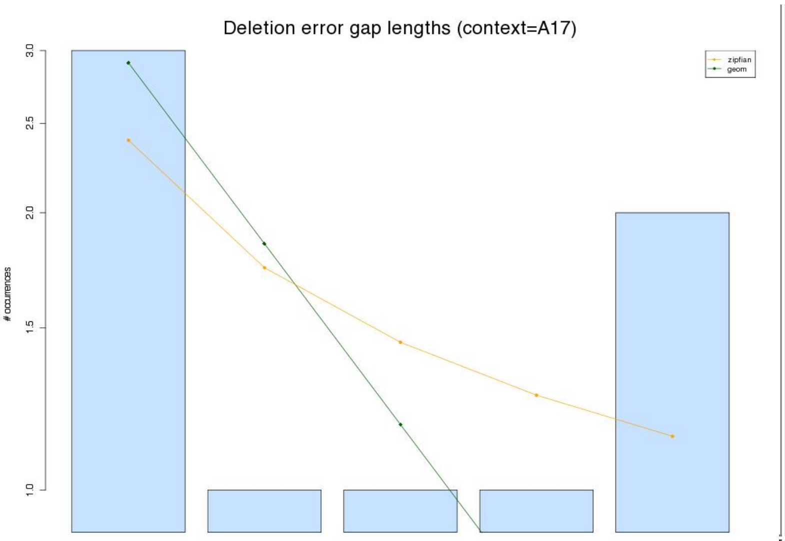	s = 0.459721443557304<br>X^2 =<br>1.30317506141628<br>p-value =<br>0.728379264884578	beta =<br>0.636363636363636<br>X^2 =<br>5.36640432990757<br>p-value =<br>0.146851087521449
T17	11	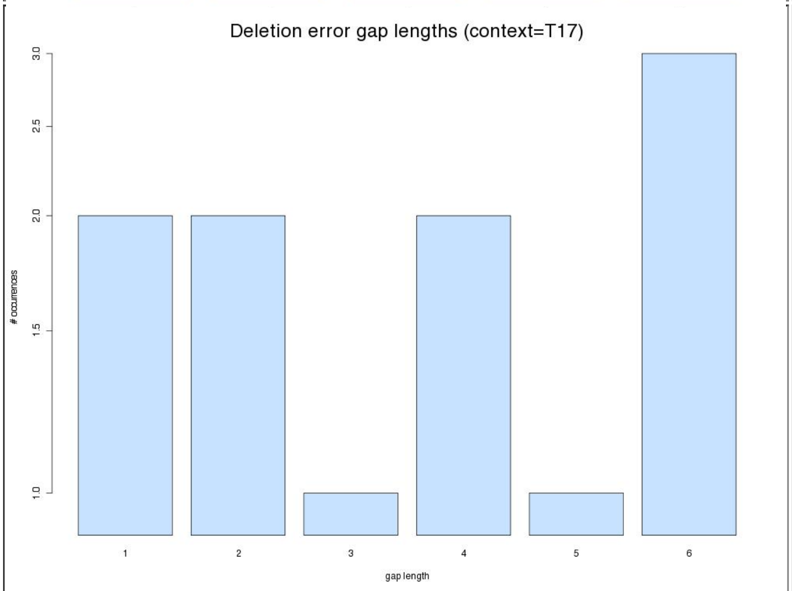	INVALID/ERROR	INVALID/ERROR
A18	6		s = 0.122967886301763<br>X^2 =<br>0.650635373179079	beta = 0.6<br>X^2 =<br>4.45696790123457

		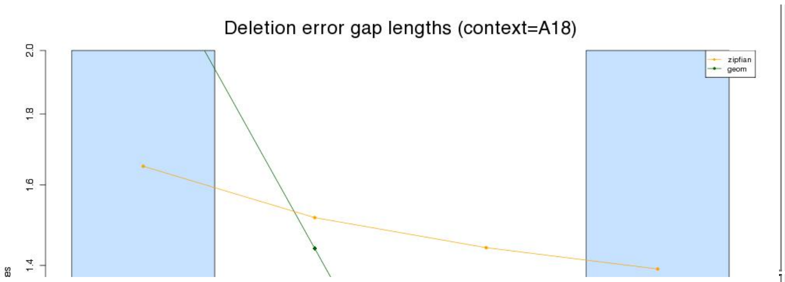	p-value = 0.722297852847837	p-value = 0.107691572180377
T18	2	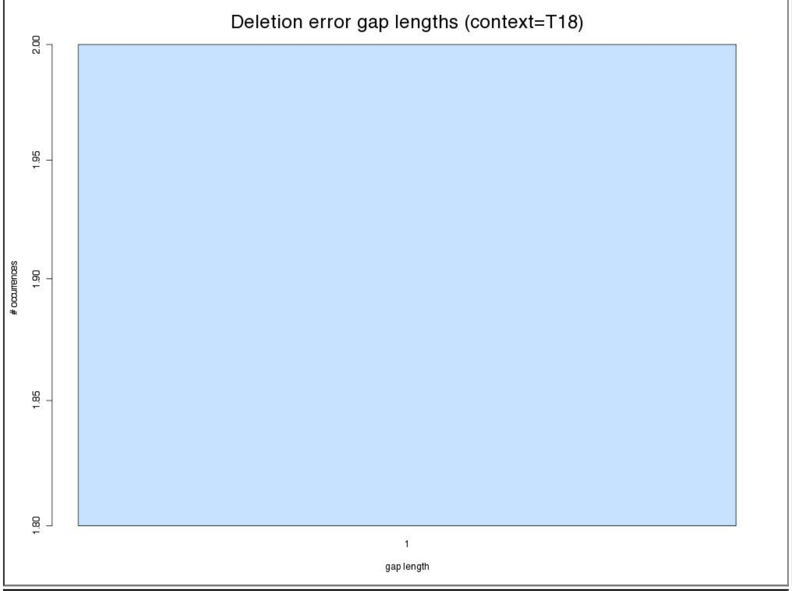	INVALID/ERROR	INVALID/ERROR
T19	13	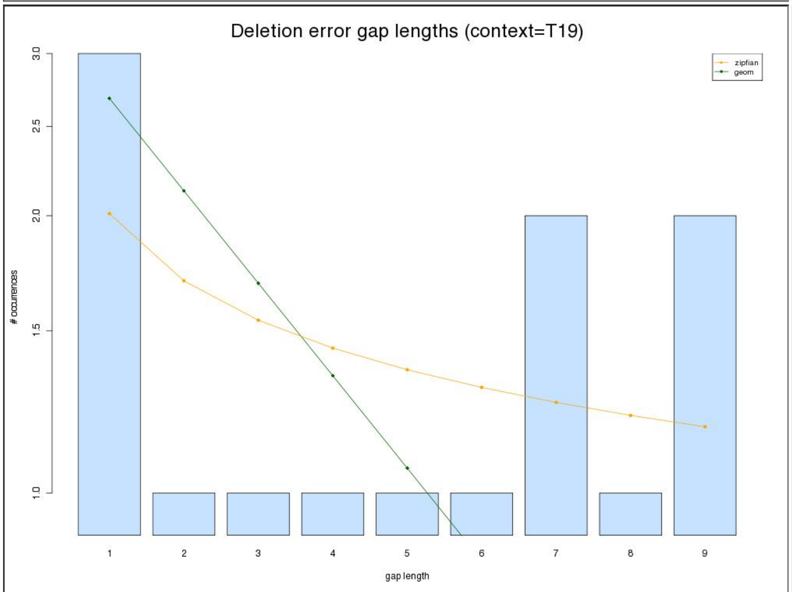	s = 0.242575021243014<br>X^2 = 2.31339813280911<br>p-value = 0.94048125810666	beta = 0.793650793650794<br>X^2 = 9.98070049857975<br>p-value = 0.189669794087238
A20	3		INVALID/ERROR	INVALID/ERROR

		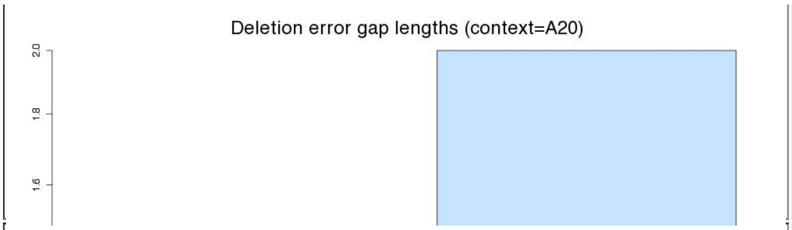		
T23	4	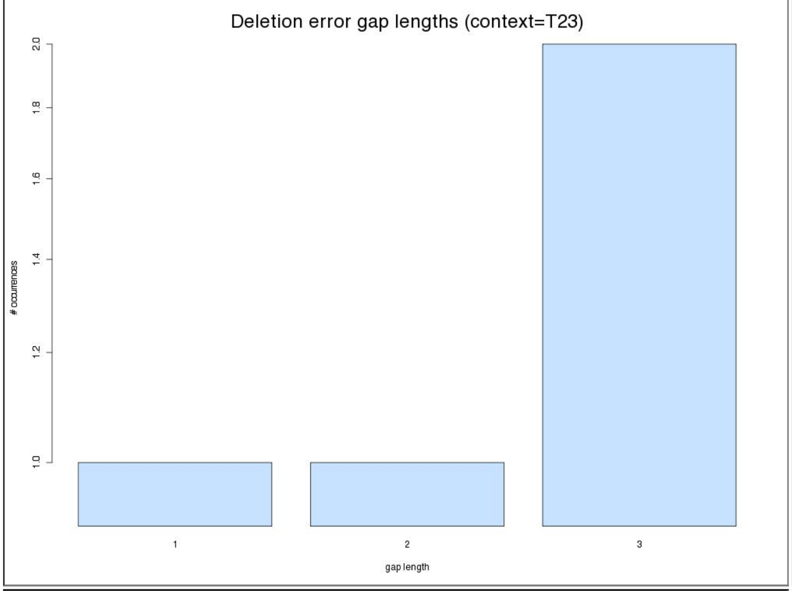	INVALID/ERROR	INVALID/ERROR
A24	8	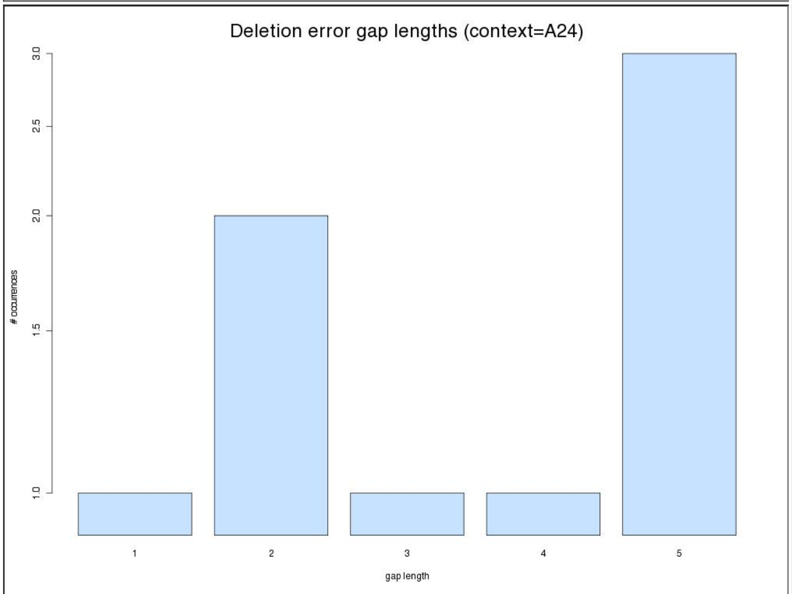	INVALID/ERROR	INVALID/ERROR

Chi square statistics

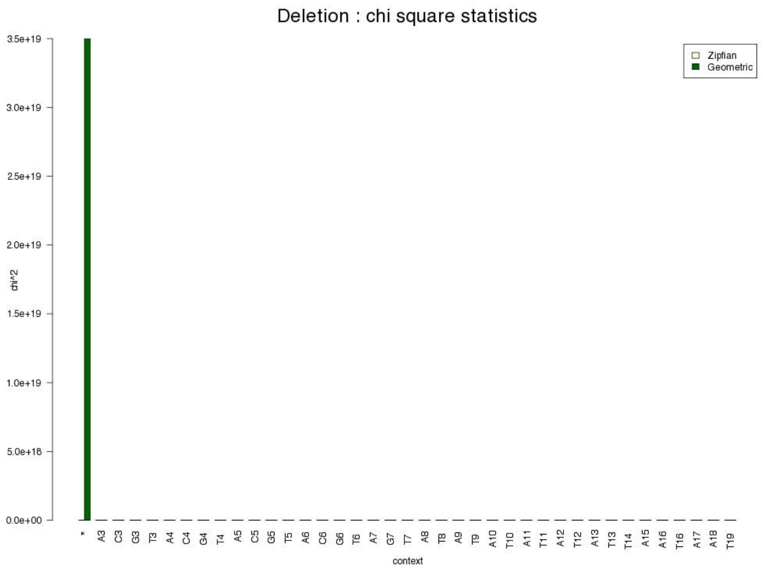

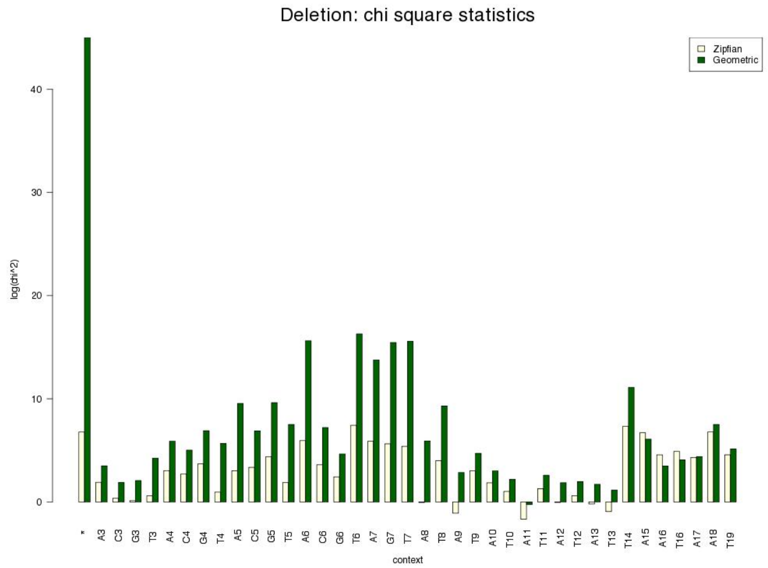

Average Pearson's residuals

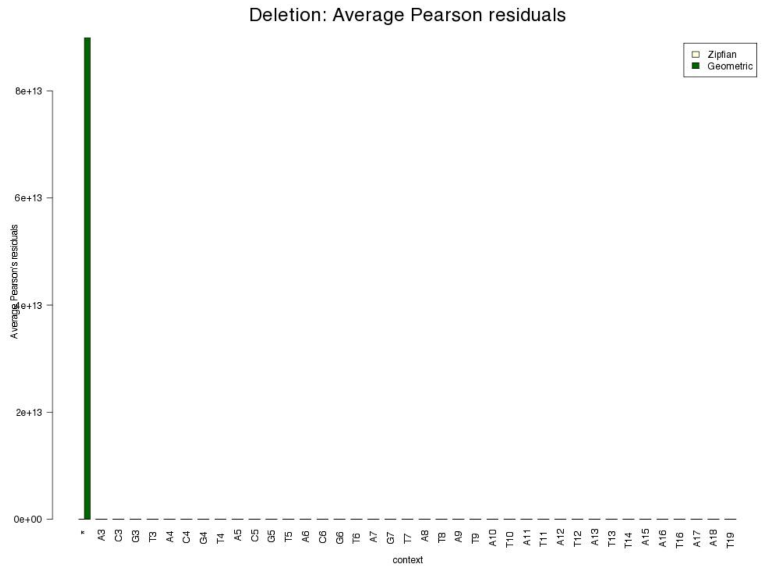

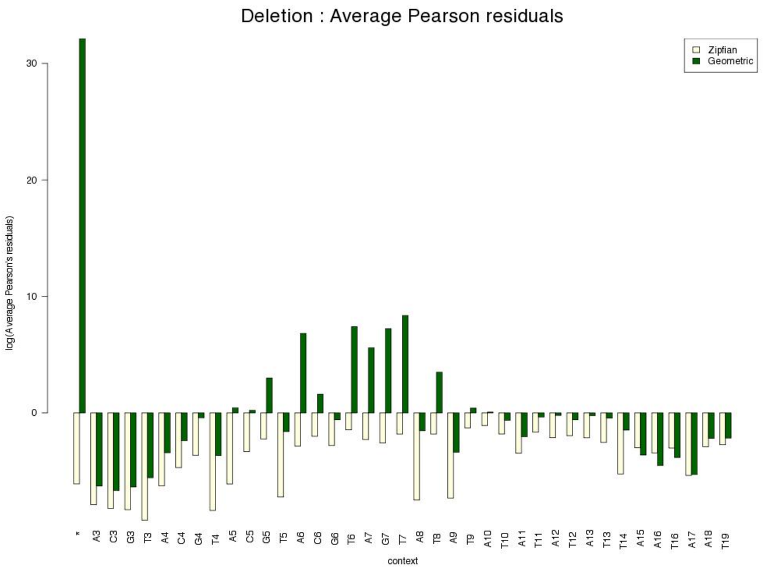

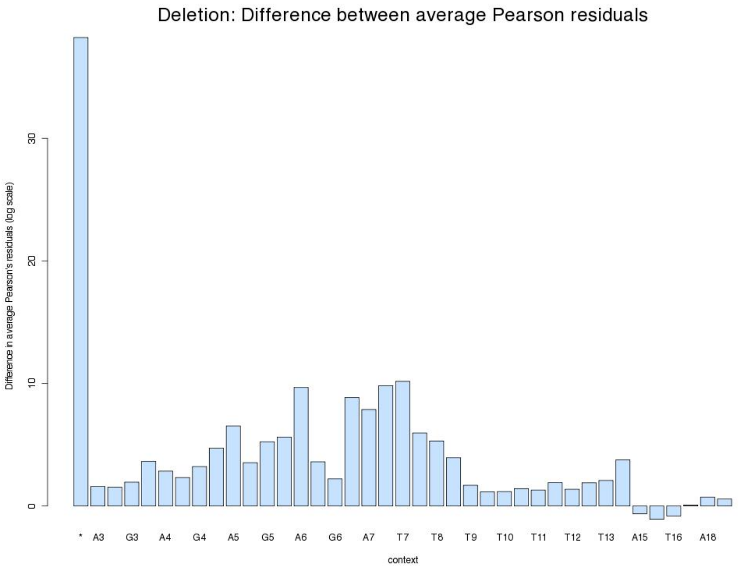

Mann U Tests

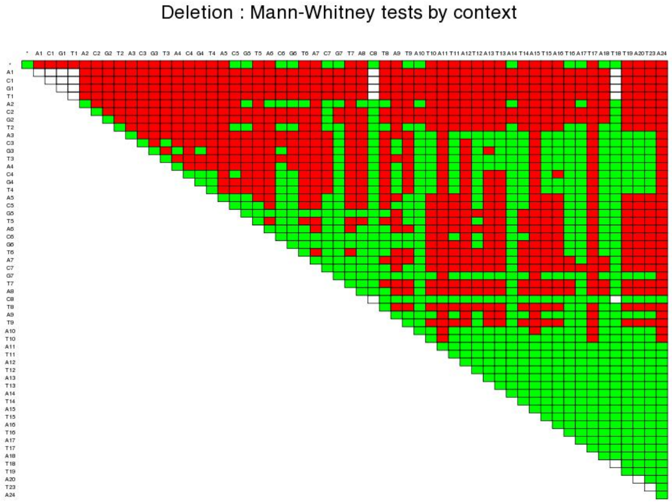

Mann U Tests by context length

Deletion : Mann-Whitney tests by context length

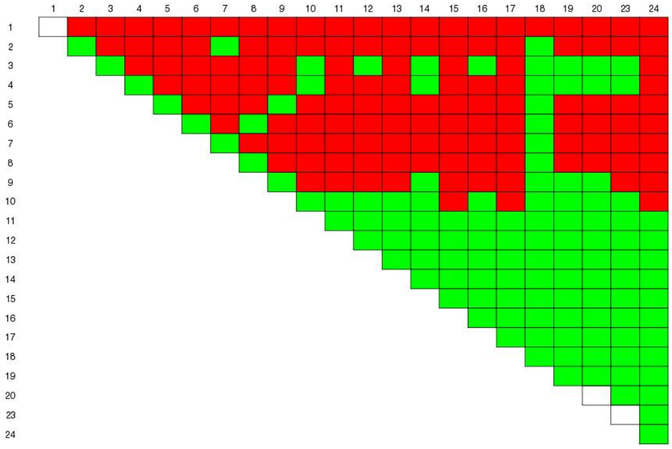

Supplement: Additional file 3 — Full results of the experiments for the Newbler assembler. [file 1756-0500-6-25-S3.pdf]
